# Terpenoid Glucosides from *Gentiana macrophylla* That Attenuate TNF-α Induced Pulmonary Inflammation in A549 Cells

**DOI:** 10.3390/molecules28186613

**Published:** 2023-09-14

**Authors:** Pei-Qi Huang, Yong-Xin Luo, Yu-Jia Zhang, Zhi-Xuan Li, Yan Wen, Kun Zhang, Dong-Li Li, Jing-Wei Jin, Ri-Hui Wu, Li-She Gan

**Affiliations:** 1 School of Biotechnology and Health Sciences, International Healthcare Innovation Institute, Wuyi University, Jiangmen 529020, China; huangpeiqi04@163.com (P.-Q.H.); lyx13556930460@163.com (Y.-X.L.); april_yhyj@163.com (Y.-J.Z.); lizhixuan202103@163.com (Z.-X.L.); wy3478044835@163.com (Y.W.); kzhang@gdut.edu.cn (K.Z.); wyuchemldl@126.com (D.-L.L.); wyuchemjjw@126.com (J.-W.J.); 2 College of Pharmaceutical Sciences, Zhejiang University, 866 Yuhangtang Road, Hangzhou 310058, China

**Keywords:** *Gentiana macrophylla* (Gentianaceae), terpenoid glucosides, pulmonary inflammation, ELISA

## Abstract

Four previously undescribed terpenoid glucosides, including one sesquiterpenoid di-glucoside (**1**), two new iridoid glucosides (**2**, **3**), and a new triterpenoid tri-glucoside (**4**), were isolated from a 70% ethanol extract of the root of *Gentiana macrophylla* (Gentianaceae), along with eight known terpenoids. Their structures were determined by spectroscopic techniques, including 1D, 2D NMR, and HRMS (ESI), as well as chemical methods. The absolute configuration of compound **1** was determined by quantum chemical calculation of its theoretical electronic circular dichroism (ECD) spectrum. The sugar moieties of all the new compounds were confirmed to be D-glucose by GC analysis after acid hydrolysis and acetylation. Anti-pulmonary inflammation activity of the iridoids were evaluated on a TNF-α induced inflammation model in A549 cells. Compound **2** could significantly alleviate the release of proinflammatory cytokines IL-1β and IL-8 and increase the expression of anti-inflammatory cytokine IL-10.

## 1. Introduction

The dry root of *Gentiana macrophylla* (Qin Jiao), a perennial herb that widely distributed in western and northern China, Mongolia, and Russia, was applied in traditional Chinese medicine for the treatment of rheumatic arthralgia and jaundice about two thousand years ago [1]. Modern pharmacological studies of the extracts demonstrated inhibitory effects on rat model of rheumatoid arthritis [2], anti-inflammatory and analgesic effects [3], as well as alleviating systemic lupus erythematosus [4], osteoporosis [5], and liver injury [6].

Phytochemical studies of *G. macrophylla* [7] demonstrated the existence of iridoids [8,9,10,11], flavonoids [12,13], lignans [14], triterpenoids [15,16], and other constituents [8,17,18]. Among these constituents, the main component iridoid gentiopicroside exhibited anti-rheumatic arthritis effect [19,20] and anti-inflammation in rat articular chondrocyte [21], as well as protective effects on liver injury [22], gastric mucosal injury [23], and neuron injury [24]. These important therapeutic effects have attracted the study of biosynthesis of the iridoids in *G. macrophylla* [25,26]. Furthermore, one of the flavonoids and luteoloside could induce G0/G1 arrest and pro-death autophagy in human non-small cell lung cancer cell lines [27].

**Figure 1:**
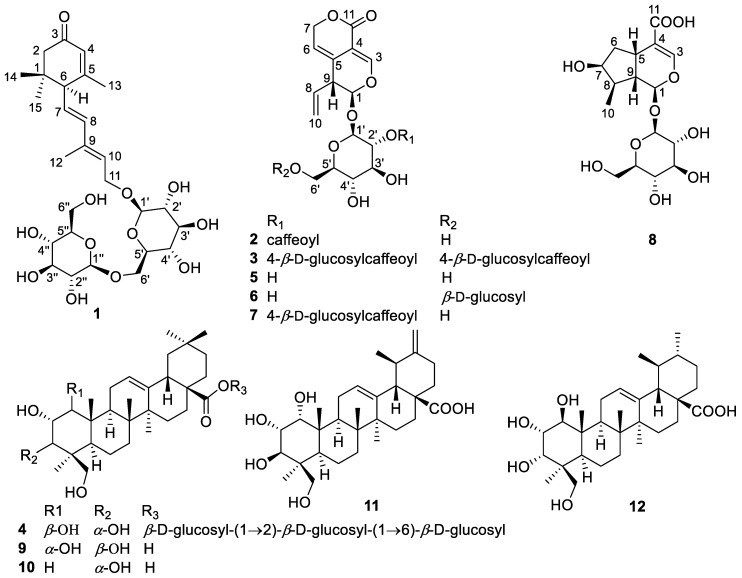
Chemical structures of compounds **1**–**12**.

In the current research, a rarely occurring sesquiterpenoid di-glucoside (**1**), two new iridoids (**2**, **3**), and a new triterpenoid tri-glucoside (**4**), together with eight known terpenoids, were isolated from the root of *G. macrophylla* (Figure 1). Their structures were determined by spectroscopic methods, chemical methods, and quantum chemical calculations. All of the iridoids (**2**, **3**, **5**–**8**) were subjected to anti-inflammatory activity evaluation on TNF-α induced pulmonary inflammation in A549 cells.

## 2. Results and Discussion

### 2.1. Identification of New Compounds

**Figure 2:**
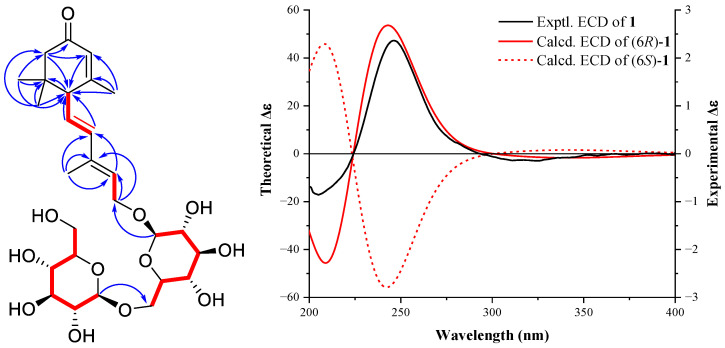
^1^H-^1^H COSY correlations (red bold lines), key HMBC correlations (blue arrows), and experimental and theoretical ECD spectra of **1**.

Compound **1** was isolated as a colorless gum, which had a molecular formula of C_27_H_42_O_12_ as deduced from an HRMS (ESI) pseudo ion peak at 581.2564 [M + Na]^+^ (calcd. for C_35_H_58_O_9_Na, 581.2569). The IR spectrum showed absorption bands for carbonyl (1656 cm^−1^) and double bond (1635 cm^−1^) functional groups. The ^1^H-NMR spectrum (Table 1) of **1** exhibited two methyl singlets linked to aliphatic carbons at δ_H_ 0.98 and 1.04, and two methyl singlets linked to double bonds at δ_H_ 1.82 and 1.93, as well as two anomeric hydrogens of glycosides at δ_H_ 4.31 (1H, d, *J* = 8.1 Hz) and 4.39 (1H, d, *J* = 7.8 Hz). Two intercoupled olefinic protons at δ_H_ 6.28 (1H, d, *J* = 15.5 Hz) and 5.64 (1H, dd, *J* = 15.5, 9.5 Hz) indicated a trans C-CH=CH-CH group. Two other olefinic hydrogens resonated at δ_H_ 5.69 (1H, dd, *J* = 7.3, 6.4 Hz) and 5.90 (1H, s) indicating the existence of two double bonds. The ^13^C-NMR (Table 1) and DEPT (135) spectra revealed a carbonyl group at δ_C_ 202.1, four olefinic methine at δ_C_ 139.3, 128.5, 127.3, and 126.0, four methyls at δ_C_ 28.1, 27.5, 23.8, and 13.1, as well as two anomeric carbons at δ_C_ 104.9 and 103.4. The above information revealed a sesquiterpenoid di-glycoside structure for **1**, similar to that of (*R*)-dehydroxyabscisic alcohol *β*-d-apiofuranosyl-(1″→6′)-*β*-d-glucopyranoside [28]. On the HMBC spectrum, correlations from the geminal dimethyl H_3_-14 and H_3_-15 to C-1, C-2, and C-6, from H_3_-13 to C-5, C-4, and C-6, from H_2_-2 to C-3, C-4, and C-6, and from H-4 to C-6 revealed the trimethyl *α*,*β*-unsaturated cyclohexanone moiety. HMBC correlations from H-7 and H-8 to C-6, from H_3_-12 to C-9, C-8, and C-10, from H-10 to C-8, and from H_2_-11 to C-9 and C-10 then indicated the conjugated double bonds sidechain linked to C-6. For the di-hexose moiety, ^1^H-^1^H COSY first allowed the confirmation of two independent glucoside chain and key HMBC correlations of H-1″/CH_2_-6′ and H-1′/CH_2_-11 then identified the connectivity as shown in Figure 2. NOESY correlations of H_3_-12/H_2_-11 and H-8/H-10 revealed the trans-configuration of C-9−C-10 double bond. Furthermore, acid hydrolysis of **1** and subsequent acetylation and GC analysis showed that the hexose was identical to an authentic sample of D-glucose. The absolute configuration at C-6 was identified by quantum chemical calculation of the theoretical ECD spectrum and compared with the experimental one. As shown in Figure 2, both the theoretical ECD spectrum for 6*R*-**1** and the experimental ECD spectrum showed the first small negative Cotton effect around 300–350 nm and a positive one around 250 nm, while the Cotton effects of the theoretical ECD spectra for 6*S*-**1** showed opposite signs to the experimental data, which indicated a 6*R* configuration for **1**. Therefore, the structure of compound **1** was deduced as ®-dehydroxyabscisic alcohol-*β*-d-glucosyl(1→6)-*β*-d-glucopyranoside.

**Figure 3:**
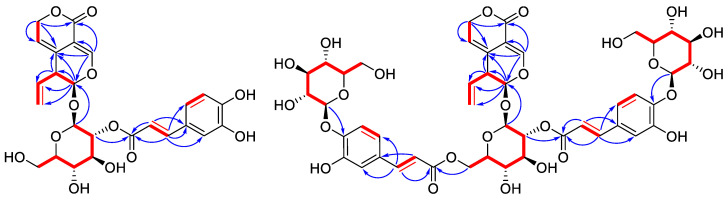
^1^H-^1^H COSY (red bold lines) and key HMBC (blue arrows) correlations of **2** and **3**.

Compound **2** exhibited a molecular formula of C_25_H_26_O_12_ based on its HRMS (ESI) data, indicating 13 indices of hydrogen deficiency. The IR spectrum presented the existence of carbonyl and double bonds. The ^1^H NMR spectrum (Table 1) of **2** showed typical signals for an iridoid acetal proton at δ_H_ 5.67 (1H, d, *J* = 2.0 Hz, H-1), an oxygen-bearing olefinic methine proton at δ_H_ 7.32 (1H, s, H-3), and three intercoupling olefinic hydrogen signals for a vinyl group at δ_H_ 5.71 (1H, ddd, *J* = 17.1, 10.3, 6.4 Hz, H-8), 5.19 (1H, br d, *J* = 17.1 Hz, H-10b), and 5.16 (1H, br d, *J* = 10.3 Hz, H-10a), which indicated a 7,8-seco-iridoid structure [8]. Two trans-double bond hydrogen signals at δ_H_ 7.51 (1H, d, *J* = 15.8 Hz) and 6.13 (1H, d, *J* = 15.8 Hz), as well as three aromatic hydrogen signals at δ_H_ 6.94 (1H, br d, *J* = 8.0 Hz), 6.78 (1H, d, *J* = 8.0 Hz), and 7.04 (1H, br s), indicated a caffeoyl moiety. On the ^13^C-NMR and DEPT (135) spectra, a typical olefinic methylene signal at *δ*_C_ 118.3 (CH_2_-10) confirmed the vinyl group. Two acetal methine at *δ*_C_ 97.9 (CH-1) and 97.5 (C-1′) confirmed the iridoid structure and showed a glycoside anomeric carbon, respectively. The above information suggested a caffeoyl-gentiopicroside structure [29]. HSQC and HMBC spectra further enabled us to elucidate the detailed structure of **2** (Figure 3). Key HMBC correlations from H-3 to C-1, C-5, and C-11, from H_2_-7 to C-11, C-6, and C-5, and from H-1 to C-5, C-8, and C-9, as well as from the anomeric H-1′ to C-1 confirmed the basic gentiopicroside structure. The H-2′ hydrogen showed a HMBC correlation with the caffeoyl ester carbonyl carbon, which is located in the caffeoyl group at C-2. Acid hydrolysis, acetylation, and GC analysis confirmed the hexose as D-glucose. Therefore, the structure of compound **2** was determined as 2′-*O*-caffeoylgentiopicroside.

Compound **3** was isolated as a brown amorphous powder. Its molecular formula was established as C_46_H_52_O_15_ by HRMS (ESI) (*m*/*z* 1027.2686 [M + Na]^+^, calcd for C_46_H_52_O_15_Na, 1027.2690). The 1D NMR data (Table 1) of **3** showed similar but more complicated signals than those of **1**. Besides the resonances for the gentiopicroside core, there are two pairs of glucosyl and caffeoyl moieties, including two pairs of trans-double bond hydrogens at δ_H_ 7.62 (1H, d, *J* = 16.0 Hz), 6.42 (1H, d, *J* = 16.0 Hz), 7.40 (1H, d, *J* = 15.9 Hz), and 6.05 (1H, d, *J* = 15.9 Hz), two pairs of caffeoyl carbonyls at *δ*_C_ 168.4, 167.7, and two pairs of glucosyl anomeric carbon signals at *δ*_C_ 103.9, 103.0. Detailed interpretation of HSQC, ^1^H-^1^H COSY, and HMBC spectra allowed the assignment of connections between these substituents (Figure 3). HMBC correlations from glucosyl anomeric hydrogens at δ_H_ 4.88 and 4.89 to two oxygen-bearing aromatic carbons at *δ*_C_ 148.7 and 149.2 (C-4 of caffeoyl) showed two 4-*O*-glucosylcaffeoyl moieties, respectively. Correlations from the H-2′ (δ_H_ 4.76) and H_2_-6′ (δ_H_ 4.40, 4.57) hydrogens to the two caffeoyl carbonyls at *δ*_C_ 167.7 and 168.4 revealed their connections via ester bonds, respectively. The hexose in compound **3** was also confirmed as D-glucose by GC analysis. The structure of compound **3** was finally elucidated as 2′,6′-bis-*O*-(4-*O*-glucosylcaffeoyl)-gentiopicroside.

**Figure 4:**
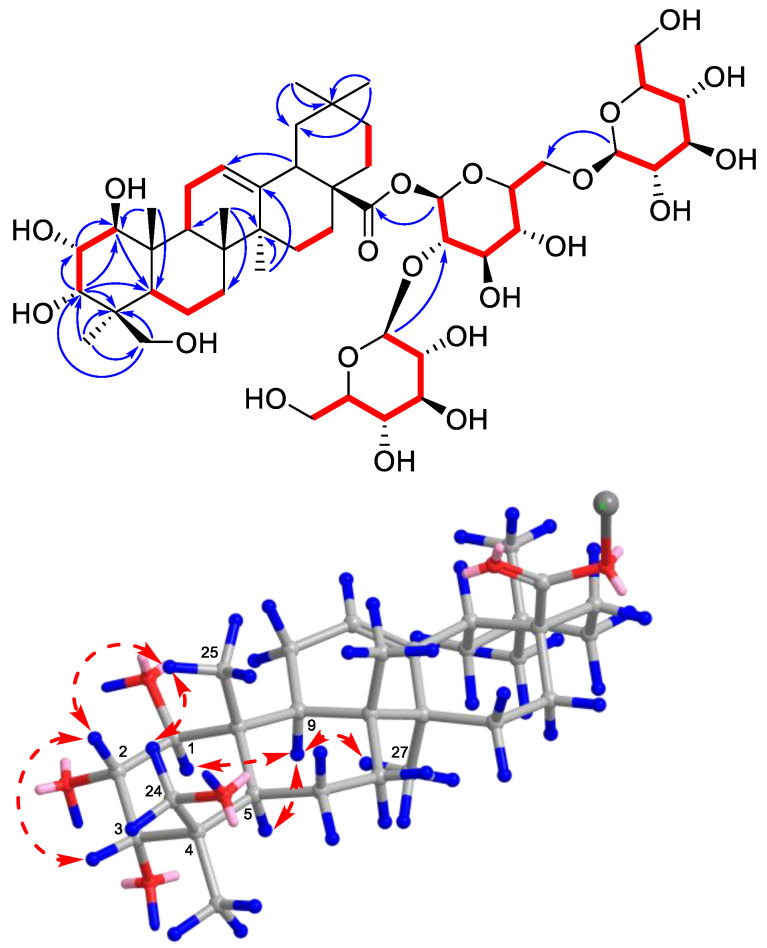
Key HMBC (blue arrows) and NOESY (red dotted double arrows) correlations of **4**.

Compound **4** was obtained as a white amorphous powder and the molecular formula was determined as C_48_H_78_O_21_ by HRMS (ESI) (*m*/*z* 991.5103 [M + H]^+^, calcd for C_48_H_79_O_21_, 991.5108). The ^1^H-NMR spectrum showed the existence of six methyl singlets at δ_H_ 1.17, 1.06, 0.98, 0.94, 0.91, 0.77 (each 3H, s), and three glycosyl anomeric hydrogens at δ_H_ 5.43 (1H, d, *J* = 8.0 Hz, H-1′), 4.80 (1H, d, *J* = 7.8 Hz, H-1″), and 4.34 (1H, d, *J* = 7.8 Hz, H-1‴). On the ^13^C- NMR and DEPT 135 spectra, an ester carbonyl carbon at δ_C_ 178.2 (C-28), one trisubstituted double bond at δ_C_ 144.0 (C-13) and 124.7 (C-12), and three glycosyl anomeric methines at δ_C_ 104.7 (C-1‴), 103.7 (C-1″), and 93.9 (C-1′), together with the above information, indicated a poly-hydroxy triterpenoid tri-glycosides structure for **4**. The six methyl singlets, one double bond, and one carboxyl carbon showed an olean-12-en-28-oic acid-type core structure [30]. This deduction was confirmed by HMBC correlations depicted in Figure 4, including key correlations of these methyl signals to carbons on the pentacyclic ring. Four hydroxyl groups were located at C-1, C-2, C-3, and C-24. The three glycosyl groups were connected by key HMBC correlations of the anomeric hydrogens to methines on another glycose or the carboxyl carbon. After the assignment of the planar structure, the relative configuration of the hydroxyl groups was established by NOESY experiments. NOESY correlations of H_3_-25/H-2, H_3_-25/H_b_-24, H-2/H-3 showed their β-orientation, while the correlation of H-9/H-1 indicated their α-orientation. Analyzing the chair-conformation of ring A, the coupling constant (11.3 Hz) between H-1 and H-2 again indicated their transaxial relationship, while the coupling constant of H-2/H-3 (2.9 Hz) showed H-3 is on the equatorial bond [31]. The chemical shifts of C-23 and C-24 are also similar to derivatives reported in the literature with the same relative configuration [32]. Acid hydrolysis, acetylation, and GC analysis confirmed the hexoses as D-glucose. Therefore, the structure of compound **4** was identified to be 1*β*,2*α*,3*α*,24-tetrahydroxyolean-12-en-28-oic acid 28-*O*-[*β*-d-glucosyl-(1→2)]-[*β*-d-glucosyl-(1→6)]-*β*-d-glucosyl ester.

By comparing their NMR data with those reported in the literatures, the eight known compounds were identified as gentiopicroside (**5**) [33], 6′-*O*-*β*-d-glucopyranosyl-gentiopicroside (**6**) [34], picrogentioside A (**7**) [29], loganic acid (**8**) [35], 1*α*,2*α*,3*β*,24-tetrahydroxyolean-12-en-28-oic acid (**9**) [30], 2*α*,3*α*,24-trihydroxyolean-12-en-28-oic acid (**10**) [36], 1*α*,2*α*,3*β*,24-tetrahydroxyursa-12,20(30)-dien-28-oic acid (**11**) [30], and 1β,2α,3α,24-tetrahydroxyurs-12-en-28-oic acid (**12**) [31], respectively.

### 2.2. Anti-Pulmonary Inflammation Activity of Iridoids

**Figure 5:**
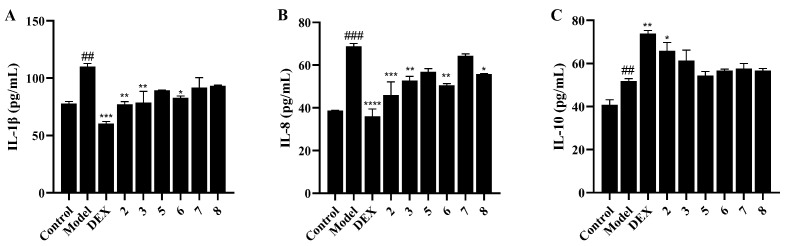
Effects of compounds **2**, **3**, **5**–**8** on cytokine release levels in TNF-α induced A549 cell inflammation. (**A**) Release level of IL-1β. (**B**) Release level of IL-8. (**C**) Release level of IL-10. ^##^ *p* < 0.01. vs. Control group; ^###^ *p* < 0.001. vs. Control group; * *p* < 0.05. vs. Model group; ** *p* < 0.01. vs. Model group; *** *p* < 0.001. vs. Model group; **** *p* < 0.0001. vs. Model group.

In order to evaluate the protective effect of iridoids on lung cell inflammation, a non-lethal concentration (10 ng/mL) of TNF-α was applied to induce an inflammatory response in A549 cells. Expression of the proinflammatory cytokine interleukin-1β (IL-1β) and interleukin-8 (IL-8), as well as the anti-inflammatory cytokine interleukin-10 (IL-10), were detected by enzyme-linked immunosorbent assay (ELISA). As shown in Figure 5, TNF-α stimulation very significantly upregulated the production of IL-1β (*p* < 0.01) and IL-8 (*p* < 0.001), while the expression of IL-10 was also increased via inverse feedback of living cells. Compounds **2**, **3**, and **6** could strongly decrease the content of IL-1β and IL-8 at 10 μM. Compound **2** could also upregulate the expression of the anti-inflammatory cytokine IL-10 significantly. The above data showed that the iridoids, especially 2′-*O*-caffeoylgentiopicroside (**2**), could effectively attenuate the inflammatory response in lung cells and might be a potential lead compound for the development of anti-pneumonia drug.

## 3. Materials and Methods

### 3.1. General Experimental Procedures

Optical rotations were determined on an Anton Paar MCP 200 automatic polarimeter (Graz, Austria). UV Spectra were recorded on a Shimadzu UV-2600 UV–Visible spectrometer. IR spectra were acquired on a Thermo Scientific Fourier Transform NICOLET iS5 Infrared Spectrometer (Waltham, MA, USA) using KBr disks. HRMS (ESI) was measured on an AB SCIEX X500R QTOF mass spectrometry. 1D and 2D NMR data were recorded on a Bruker AVANCE NEO 500 spectrometer (Bremen, Germany). Chemical shift values were expressed in δ (ppm) relative to tetramethylsilane (TMS) as the internal standard. ECD spectra were measured on a Chirascan spectrometer (England, United Kingdom). Precoated silica gel 60 GF254 plates (Branch of Qingdao Haiyang Chemical Co., Ltd., Qindao, China) was used for TLC. Silica gel (200–300 mesh and 300–400 mesh, Qingdao Haiyang Chemical Co., Ltd., Qingdao, China), silica gel for chromatography C18 MB 100-40/75 (Fuji Chemical industries Co., Ltd., Japan) and D-101 macroporous adsorbent resin (Shanghai Macklin Biochemical Co., Ltd., Shanghai, China) were used for column chromatography (CC). A Waters 1500-Series system was applied to perform HPLC separations. For reversed-phase semipreparative HPLC, a YMC Pack ODS-A C18 (250 × 10 mm, 5 μm) column was applied.

### 3.2. Plant Material

Dry root of *G. macrophylla* Pall. was purchased from Dali city, Yunnan province of P. R. China and was authenticated by Prof. Youkai Xu of Xishuangbanna Tropical Botanical Garden, Chinese Academy of Sciences. A voucher specimen (GM2021A) was deposited in the School of Biotechnology and Health Sciences, Wuyi University (Jiangmen, China).

### 3.3. Extraction and Isolation

The air-dried roots of *G. macrophylla* (10 kg) were powdered and extracted with 70% EtOH (3 × 45 L, 7 days for each time) at room temperature. The solvent was evaporated under reduced pressure to obtain a crude extract (4.6 kg), which was then suspended in H_2_O (2.5 L) and successively partitioned with petroleum ether (PE, 4 × 2.5 L), EtOAc (4 × 2.5 L), and *n*-BuOH (4 × 2.5 L). The PE and EtOAc extracts were combined (100.1 g) and then the *n*-BuOH fraction (771.5 g) was obtained. 

The EtOAc fraction was first subjected to a D-101 macroporous resin column eluted with aqueous MeOH (30% to 100%, stepwise) to afford seven subfractions A1–A7. Compound **5** (24.2 g) was obtained by recrystallization of fraction A2. A4 (8.1 g) was further chromatographed over a silica gel column (CH_2_Cl_2_–MeOH, 100:1 to 0:100, stepwise) to obtain eleven sub-fractions, A4A–A4K. A4L (859.4 mg) was separated by a C18 reverse-phased silica gel column (from 60% to 100% aqueous MeOH) to afford six subfractions A4L1–A4L6 and a pure compound **11** (2.5 mg). A4L6 was further chromatographed by an RP-C18 column on a semi-preparative HPLC (CH_3_CN–H_2_O, 40:60, 4 mL/min) to yield compounds **12** (22.8 mg, *t*_R_ = 20.1 min) and **9** (5.1 mg, *t*_R_ = 21.8 min).

The *n*-BuOH fraction was chromatographed on an MCI gel column eluted with MeOH–H_2_O gradient to obtain six major fractions B1–B6. Fraction B2 (50.9 mg) was separated by an RP-C18 column on a semi-preparative HPLC (CH_3_CN–H_2_O, 18:82, 4 mL/min) to obtain compounds **8** (10.5 mg, *t*_R_ = 9.5 min) and **6** (10.0 mg, *t*_R_ = 10.7 min). Fraction B5 (16.9 g) was separated by using silica gel CC eluted with CH_2_Cl_2_–MeOH (100:1, 80:1, 50:1, 30:1, 20:1, 10:1, 5:1, 2:1, each 1 L, *v*/*v*) to give Fr. B5A–B5V. Fr. B5O (149.3 mg) was separated by a reverse-phased C-18 gel column MeOH–H_2_O (from 4:6 to 8:2, *v*/*v*) to give seven sub-fractions, B5O1-B5O7. B5O3 was purified by an RP-C18 column on a semi-preparative HPLC (CH_3_CN–H_2_O, 17.5:82.5, 4 mL/min) to obtain compound **2** (6.7 mg, *t*_R_ = 17.5 min). B5S (223 mg) was purified by a semi-preparative HPLC (CH_3_CN–H_2_O, 22.5:77.5, 4 mL/min) to give compound **1** (1.7 mg, *t*_R_ = 34.6 min). Fr. B5T (623.6 mg) was chromatographed on a silica gel column eluted with CH_2_Cl_2_–MeOH (50:1 to 0:100, *v*/*v*) to get Fr. B5T1~Fr. B5T2. Fr. B5T1 (779.2 mg) was finally purified by an RP-C18 column on a semi-preparative HPLC (CH_3_CN–H_2_O, 20:80, 4 mL/min) to yield compounds **7** (1.6 mg, *t*_R_ = 35.1 min) and **3** (5.0 mg, *t*_R_ = 37.2 min). Compound **10** (4.5 mg) was obtained by an RP-C18 column chromatography eluted with aqueous MeOH (from 25% to 100%, *v*/*v*). B5V was separated by an RP-C18 column on a semi-preparative HPLC (CH_3_CN–H_2_O, 21:79, 4 mL/min) to yield compound **4** (2.4 mg, *t*_R_ = 29.9 min).

*(S)-Dehydroxyabscisic alcohol-β-d-glucosyl(1→6)-β-d-glucopyranoside* (**1**): Colorless gum; [α${]}_{D}^{20}$− 218.7 (*c* 0.16, MeOH); ECD (MeOH, c = 0.40 mg/mL) *λ*_max_ (Δε) 246 (2.36), 306 (−0.11) nm; IR (KBr) *ν*_max_ 3373, 2948, 2842, 1656, 1635, 1411, 1031, 635 cm^−1^; ^1^H- and ^13^C-NMR data, see Table 1; HRMS (ESI) (positive) *m*/*z* 581.2564 [M + Na]^+^ (calcd for C_27_H_42_O_12_Na, 581.2569); also see Supplementary Figures S1–S9.
*2*′*-O-Caffeoylgentiopicroside* (**2**): Brown amorphous powder; [α${]}_{D}^{20}$− 266.6 (*c* 0.30, MeOH); UV (MeOH) nm *λ*_max_ 216 (3.54), 246 (3.49), 284 (3.44), 332 (3.52); IR (KBr) *ν*_max_ 3355, 2919, 2849, 1627, 1608, 1579, 1410, 1271, 1058, 1033 cm^−1^; ^1^H- and ^13^C-NMR data, see Table 1; HRMS (ESI) (positive) *m*/*z* 541.1411 [M + Na]^+^ (calcd for C_25_H_26_O_12_Na, 541.1417); also see Supplementary Figures S10–S18.
*2*′*,6*′*-Bis-O-(4-O-glucosylcaffeoyl)-gentiopicroside* (**3**): Brown amorphous powder; [α${]}_{D}^{20}$− 110.7 (*c* 0.56, MeOH); UV (MeOH) nm *λ*_max_, 214 (4.38), 240 (4.18), 285 (4.28), 321 (4.10); IR (KBr) *ν*_max_ 3358, 2921, 2851, 1657, 1630, 1466, 1410, 1076 cm^−1^; ^1^H- and ^13^C-NMR data, see Table 1; HRMS (ESI) (positive) *m*/*z* 1027.2686 [M + Na]^+^ (calcd for C_46_H_52_O_15_Na, 1027.2690); also see Supplementary Figures S19–S27.
*1β,2α,3α,24-Tetrahydroxyolean-12-en-28-oic acid 28-O-[β-d-glucosyl-(1→2)]-[β-d-glucosyl-(1→6)]-β-d-glucosyl ester* (**4**): White amorphous powder; [α${]}_{D}^{20}$+ 4.3 (*c* 0.23, MeOH); IR (KBr) *ν*_max_ 3679, 2922, 1055, 1033, 1013 cm^−1^; ^1^H-NMR data *δ*_H_ (J in Hz): 5.43 (1H, d, 8.0, H-1′), 5.24 (1H, dd, 3.7, 3.5, H-12), 4.80 (1H, d, 7.8, H-1″), 4.34 (1H, d, 7.8, H-1‴), 4.11 (1H, br d, 11.6, H-6′), 3.90 (1H, dd, 11.3, 2.4, H-6″), 3.84 (2H, overlapped, H-2′, 6‴), 3.83 (1H, d, 2.9, H*_β_*-3), 3.78 (1H, dd, 11.6, 4.4, H-6′), 3.67 (1H, overlapped, H_b_-24), 3.65 (2H, overlapped, H-3′, 6‴), 3.62 (1H, overlapped, H-6″), 3.61 (1H, overlapped, H*_β_*-2), 3.50 (2H, overlapped, H-4′, 5′), 3.45 (1H, d, 11.3, H*_α_*-1), 3.41 (1H, d, 11.3, H_a_-24), 3.35 (2H, overlapped, H-3″, 3‴), 3.28 (2H, overlapped, H-4‴, 5‴), 3.25 (1H, m, H-5″), 3.20 (1H, m, H-2″), 3.19 (1H, m, H-2‴), 3.13 (1H, br t, 9.3, H-4″), 2.83 (1H, dd, 13.7, 3.9, H*_β_*-18), 2.45 (1H, m, H*_α_*-11), 2.08 (1H, m, H*_β_*-11), 2.00 (1H, m, H*_α_*-16), 1.90 (1H, m, H*_α_*-9), 1.85 (1H, m, H*_β_*-16), 1.73 (1H, m, H*_α_*-15), 1.70 (1H, m, H*_β_*-19), 1.69 (1H, m, H*_α_*-22), 1.62 (1H, m, H*_β_*-22), 1.53 (1H, m, H*_β_*-6), 1.48 (1H, m, H*_α_*-6), 1.46 (1H, m, H*_α_*-7), 1.41 (2H, overlapped, H*_α_*-5, H*_α_*-21), 1.35 (1H, m, H*_β_*-7), 1.21 (1H, m, H*_β_*-21), 1.17 (3H, s, Me-27), 1.14 (1H, m, H*_α_*-19), 1.06 (3H, s, Me-23), 1.02 (1H, m, H*_β_*-15), 0.98 (3H, s, Me-25), 0.94 (3H, s, Me-30), 0.91 (3H, s, Me-29), 0.77 (3H, s, Me-26); ^13^C-NMR data *δ*_C_: 178.2 (C-28), 144.0 (C-13), 124.7 (C-12), 104.7 (C-1‴), 103.7 (C-1″), 93.9 (C-1′), 81.2 (C-1), 78.7(C-3′), 78.2 (C-2′), 78.0 (C-3″, 5″, 3‴, 5‴), 77.8 (C-5′), 75.8 (C-2″), 75.2 (C-3, 2‴), 72.5 (C-4″), 71.7 (C-2), 71.5 (C-4‴), 70.7 (C-4′), 69.5 (C-6′), 65.7 (C-24), 63.7 (C-6″), 62.7 (C-6‴), 49.9 (C-9), 49.5 (C-5), 48.0 (C-17), 47.2 (C-19), 44.9 (C-4), 44.3 (C-10), 42.7 (C-14), 42.4 (C-18), 41.5 (C-8), 34.9 (C-21), 34.5 (C-7), 33.5 (C-29), 33.1 (C-22), 31.6 (C-20), 29.8 (C-15), 28.2 (C-11), 26.5 (C-27), 24.2 (C-30), 23.5 (C-16), 23.1 (C-23), 19.4 (C-6), 18.0 (C-26), 13.6 (C-25); HRMS (ESI) (positive) *m*/*z* 991.5103 [M + H]^+^ (calcd for C_48_H_79_O_21_, 991.5108); also see Supplementary Figures S28–S36.

### 3.4. Acid Hydrolysis, Acetylation, and GC Analysis of Compounds **1**–**4**

The structures and configurations of the sugar moieties in compounds **1**–**4** were confirmed according to our published method [37]. Compounds **1**–**4** (0.3–1.0 mg of each) were added to 2–4 mL of 2 M aqueous HCl and reacted at 90 °C for two hours. Each reaction mixture was diluted with H_2_O and extracted with EtOAc (3 × 5 mL). The water layer was concentrated under reduced pressure to dryness. A total of 4 mg Hydroxylamine hydrochloride and 0.5 mL anhydrous pyridine were added and the reaction was carried out in a constant temperature oil bath at 90 °C for one hour. After that, 0.5 mL acetic anhydride was added to the mixture and the reaction was continued for one more hour. The obtained samples were directly used for GC-MS analysis, and the structure of the glycosyl moiety was confirmed by comparing it with the retention time of the standard D-glucose derivative prepared in the same way.

Analysis condition of GC-MS: the temperature of the injector is 250 °C; Injection volume 1.0 μL; Split ratio 41.667:1; Carrier gas helium, flow rate 1.2 mL∙min^−1^; Chromatographic column: hp-5ms (250) μm × 30 m, 0.25 μm; Column initial temperature was 60 °C (0.8 min), rising to 220 °C (3 min) at 18 °C/min, rising to 300 °C (15 min) at 14 °C/min, and dropping to 280 °C (0 min) at 10 °C /min; Detector: MSD 230 °C. Retention time for the standard D-glucose derivative was *t*_R_ = 10.94 min.

### 3.5. Quantum Chemical Calculation of ECD Spectrum of 1

The absolute configurations of **1** were determined by quantum chemical calculations of its theoretical ECD spectrum. The theoretical CD spectra of both 6R-**1** and 6S-**1** were studied. Conformational analyses were first carried out via Monte Carlo searching using molecular mechanisms with MMFF force field in the Spartan 18 program [38]. The results showed 7 lowest energy conformers for 6R-**1** and 5 ones for 6S-**1** within an energy window of 2.0 Kcal/mol. Those conformers were then reoptimized using DFT at the B3LYP/6-31G(d) level using the Gaussian 09 program [39]. The B3LYP/6-31G(d) harmonic vibrational frequencies were further calculated to confirm their stability. Six stable conformers for 6R-**1** and three ones for 6S-**1** (ΔG within 2.0 Kcal/mol) with no imaginary frequencies were, respectively refined and considered for the next steps. The electronic excitation energies and rotatory strengths (velocity) of the first 60 excited states of these conformers were calculated using the TDDFT methodology at the M062X/TZVP level in the gas phase. The ECD spectra were simulated by the overlapping Gaussian function [40] (σ = 0.48 eV), in which velocity rotatory strengths of all the excited states were adopted. To get the final ECD spectra, the simulated spectra of the lowest energy conformers were averaged according to the Boltzmann distribution theory and their relative Gibbs free energy (ΔG) (see Supplementary Figures S37 and S38 and Tables S1–S4).

### 3.6. Reagents

All the isolates and dexamethasone were dissolved in DMSO (less than 0.1% in the cell-based assays). Fetal bovine serum (FBS) was purchased from Cytiva (Marlborough, MA, USA). Phosphate-buffered saline (PBS) powder was purchased from Beijing Langeco Technology Co., Ltd. (Shunyi District, Beijing, China). RPMI 1640 medium, penicillin-streptomycin (P/S), and pancreatin were purchased from Life Technologies (Grand Island, NY, USA). All the chemicals (analytical grade) were obtained from Shanghai Macklin Biochemical Technology Co., Ltd. (Pudong Zhangjiang High Tech. Park, Shanghai, China), except those otherwise specified. The TNF-α, IL-1β, IL-8, and IL-10 ELISA kits were purchased from Shanghai Enzyme-linked Biotechnology Co., Ltd. (Minhang District, Shanghai, China).

### 3.7. Cell Culture and Cytotoxicity Test

Human lung adenocarcinoma cells (A549) were acquired from American Type Culture Collection (Manassas, VA, USA). Cells were cultured in RPMI 1640 medium (Gibco, Grand Island, NY, USA) supplemented with 10% fetal bovine serum (Cytiva, Marlborough, MA, USA) and 1% penicillin-streptomycin (P/S) (Gibco) at 37 °C with 95% humidity and 5% CO_2_.

Cytotoxicity of compounds was tested by the MTT method according to the manufacturer’s instructions. A549 cells were seeded (5 × 10^3^/well) into 96-well plates for 24 h. In addition to an untreated control group, compounds (10, 20, 40, 80, or 160 μM) were added to cells and TNF-α (final concentration,10 ng/mL) was added in after 2 h of incubation. Each group had 3 duplicate wells. A total of 24 h later and 10 μL MTT (final concentration, 0.5 mg/mL) was added and incubated at 37 °C for 4h. Optical density (OD) values were detected with a microplate reader (Thermo Fisher Scientific, Waltham, MA, USA) at 550 nm.

### 3.8. Cytokines ELISA Assay

Levels of IL-8, IL-1β, and IL-10 in culture supernatants were detected by ELISA. A549 cells (5 × 10^4^ cell/100 mL) were seeded into 96-well plates for 24h. After pre-incubation with compounds (10 μM) for 2 h and stimulation with TNF-α for 24 h, culture supernatants were harvested. Each group had 3 duplicate wells. Dexamethasone (1 μM) was used as a positive control. After centrifugation at 12,000 rpm for 10 min, supernatants were collected. An ELISA Kit (Enzyme-linked Biotechnology, Shanghai, China) was used to measure protein expression levels according to the manufacturer’s protocols.

### 3.9. Statistical Analysis

Statistical analyses were performed by Graphpad Prism 6.0 (GraphPad Software, version 6.0, San Diego, CA, USA). The data were expressed as mean ± S.D. based on at least three independent experiments. Statistical differences were accepted as significant at *p*-values less than 0.05, analyzed by one-way ANOVA with Dunnett’s test in multiple comparisons.

## 4. Conclusions

Phytochemical investigation on the roots of *G. macrophylla* led to the isolation of 12 terpenoids, including one rarely occurring sesquiterpenoid di-glucoside (**1**), two new iridoid glucosides (**2**, **3**), and a new triterpenoid tri-glucoside (**4**). Several iridoids were found to alleviate inflammatory response in A549 cells by reducing the release of proinflammatory cytokine IL-1β and IL-8, as well as increasing the expression of anti-inflammatory cytokine IL-10, which indicated that they may have therapeutic potential for pneumonia.

## Figures and Tables

**Table 1:** ^1^H-(500 MHz, *δ* in ppm, *J* in Hz) and ^13^C-NMR (125 MHz, *δ* in ppm) data of compounds **1**–**3** in CD_3_OD.

No.	1		2		3			
	δ_H_	δ_C_	δ_H_	δ_C_	δ_H_		δ_C_	
1	-	37.4	5.67 d (2.0)	97.9	5.67 d (1.9)		97.7	
2	α 2.08 d (16.8) β 2.44 d (16.8)	48.5	/	/	/		/	
3	-	202.1	7.32 s	149.4	7.29 s		149.4	
4	5.90 s	126.0	-	105.3	-		105.2	
5	-	166.0	-	126.6	-		126.5	
6	2.75 d (9.5)	57.3	5.60 m	118.1	5.55 m		118.1	
7	5.64 dd (15.5, 9.5)	127.3	4.84 overlapped 4.95 overlapped	70.6	4.71 overlapped 4.83 overlapped		70.6	
8	6.28 d (15.5)	139.3	5.71 ddd (17.1, 10.3, 6.4)	134.7	5.70 ddd (17.1, 10.4, 6.4)		134.7	
9	-	137.9	3.28 br d (6.4)	46.4	3.27 br d (6.4)		46.3	
10	5.69 dd (7.3, 6.4)	128.5	a 5.16 br d (10.3) b 5.19 br d (17.1)	118.3	a 5.14 dd (10.4, 1.5) b 5.17 dd (17.1, 1.5)		118.2	
11	a 4.35 dd (12.7, 7.3) b 4.51 dd (12.7, 6.4)	66.6	-	166.0	-		165.8	
12	1.82 s	13.1	/	/	/		/	
13	1.93 s	23.8	/	/	/		/	
14	β 0.98 s	27.5	/	/	/		/	
15	α 1.04 s	28.11	/	/	/		/	
1′	4.31 d (8.1)	103.4	4.85 overlapped	97.5	4.87 overlapped		97.2	
2′	3.19 m	75.1	4.76 dd (9.0, 8.6)	74.5	4.76 m		74.6	
3′	3.35 overlapped	77.9	3.62 dd (9.1, 9.0)	75.7	3.71 m		75.5	
4′	3.35 overlapped	71.5	3.37 m	71.7	3.38 m		72.2	
5′	3.45 m	77.1	3.41 m	78.6	3.78 m		75.7	
6′	3.79 dd (11.5, 5.8) 4.16 dd (11.5, 2.1)	69.8	3.70 dd (11.8, 5.8) 3.93 br d (11.8)	62.7	4.40 dd (11.8, 8.1) 4.57 dd (11.8, 2.1)		64.8	
	6′-glucosyl		2′-caffeoyl		2′-caffeoyl		6′-caffeoyl	
1	4.39 d (7.8)	104.9	-	127.6	-	130.8	-	131.2
2	3.22 m	75.0	7.04 br s	115.3	7.04 d (2.0)	116.7	7.15 d (2.0)	116.2
3	3.35 overlapped	77.9	-	146.7	-	148.3	-	148.7
4	3.26 m	71.6	-	149.7	-	148.7	-	149.2
5	3.28 m	78.0	6.78 d (8.0)	116.4	7.13 d (8.5)	117.8	7.25 d (8.4)	118.6
6	3.66 dd (11.9, 5.1) 3.87 dd (11.9, 1.8)	62.8	6.94 br d (8.0)	123.2	6.89 dd (8.5, 2.0)	121.9	7.08 dd (8.4, 2.0)	122.1
7	/	/	7.51 d (15.8)	147.6	7.40 d (15.9)	146.7	7.62 d (16.0)	146.4
8	/	/	6.13 d (15.8)	114.8	6.05 d (15.9)	116.9	6.42 d (16.0)	117.2
9	/	/	-	168.0	-	167.7	-	168.4
					4″-glucosyl		4″″-glucosyl	
1	/	/	/	/	4.88 overlapped	103.0	4.89 overlapped	103.9
2	/	/	/	/	3.56 overlapped	74.8	3.56 overlapped	74.8
3	/	/	/	/	3.52 overlapped	77.5	3.52 overlapped	77.5
4	/	/	/	/	3.44 m	71.7	3.41 m	71.7
5	/	/	/	/	3.49 overlapped	78.4	3.49 overlapped	78.5
6	/	/	/	/	3.73 overlapped 3.92 overlapped	62.4	3.73 overlapped 3.92 overlapped	62.7

## Data Availability

The data presented in this study are available on request from the corresponding author.

## Supplementary Materials

The following supporting information can be downloaded at: https://www.mdpi.com/article/10.3390/molecules28186613/s1. Figures S1–S36: 1D and 2D NMR, MS, and IR spectra of compounds **1**–**4**. Figure S37. B3LYP/6-31G(d) optimized lowest energy conformers for 6*R*-**1**. Figure S38. B3LYP/6-31G(d) optimized lowest energy conformers for 6*S*-**1**. Table S1: Energy (298.15 K) analysis for 6*R*-**1**. Table S2: Energy (298.15 K) analysis for 6*S*-**1**. Table S3: Cartesian coordinates for conformers of 6*R*-**1**. Table S4: Cartesian coordinates for conformers of 6*S*-**1**.

## Sample Availability

Samples of all the isolated compounds are available from the authors.
