# Supplementary material for: Terpenoid Glucosides from Gentiana macrophylla That Attenuate TNF-α Induced Pulmonary Inflammation in A549 Cells
Source: Molecules. 2023 Sep 14;28(18):6613. doi: 10.3390/molecules28186613 (PMC10535684; doi:10.3390/molecules28186613)
Supplement: Supplementary file 1 [file molecules-28-06613-s001.zip › molecules-2513290-SI.pdf]

molecules

MDPI

Supplementary Materials

Terpenoid Glucosides from *Gentiana macrophylla* That Attenuate TNF-*α* Induced Pulmonary Inflammation in A549 Cells

Pei-Qi Huang [1,†], Yong-Xin Luo [1,†], Yu-Jia Zhang [1], Zhi-Xuan Li [1], Yan Wen [1], Kun Zhang [1], Dong-Li Li [1], Jing-Wei Jin [1], Ri-Hui Wu [1,*] and Li-She Gan [1,2,*]

[1] School of Biotechnology and Health Sciences, International Healthcare Innovation Institute, Wuyi University, Jiangmen 529020, China; huangpeiqi04@163.com (P.-Q.H.); lyx13556930460@163.com (Y.-X.L.); april_yhyj@163.com (Y.-J.Z.); lizhixuan202103@163.com (Z.-X.L.); wy3478044835@163.com (Y.W.); kzhang@gdut.edu.cn (K.Z.); wyuchemldl@126.com (D.-L.L.); wyuchemjjw@126.com (J.-W.J.)

[2] College of Pharmaceutical Sciences, Zhejiang University, 866 Yuhangtang Road, Hangzhou 310058, China

* Correspondence: wyuchemwrh@126.com (R.-H.W.); ganlishe@163.com (L.-S.G.)

† These authors contributed equally to this work.

Contents

Figure S1. The ^{1}H-NMR spectrum of compound **1** in CD_3OD........1

Figure S2. The ^{13}C-NMR spectrum of compound **1** in CD_3OD........2

Figure S3. The DEPT-135 NMR spectrum of compound **1** in CD_3OD........3

Figure S4. The HSQC spectrum of compound **1** in CD_3OD........4

Figure S5. The ^{1}H-^{1}H COSY spectrum of compound **1** in CD_3OD........5

Figure S6. The HMBC spectrum of compound **1** in CD_3OD........6

Figure S7. The NOESY spectrum of compound **1** in CD_3OD........7

Figure S8. The (+)-HRESIMS spectroscopic data of compound **1**........8

Figure S9. The IR spectrum of compound **1**........9

Figure S10. The ^{1}H-NMR spectrum of compound **2** in CD_3OD........10

Figure S11. The ^{13}C-NMR spectrum of compound **2** in CD_3OD........11

Figure S12. The DEPT-135 NMR spectrum of compound **2** in CD_3OD........12

Figure S13. The HSQC spectrum of compound **2** in CD_3OD........13

Figure S14. The ^{1}H-^{1}H COSY spectrum of compound **2** in CD_3OD........14

Figure S15. The HMBC spectrum of compound **2** in CD_3OD........15

Figure S16. The NOESY spectrum of compound **2** in CD_3OD........16

Figure S17. The (+)-HRESIMS spectroscopic data of compound **2**........17

Figure S18. The IR spectrum of compound **2**........18

Figure S19. The ^{1}H-NMR spectrum of compound **3** in CD_3OD........19

Figure S20. The 13C-NMR spectrum of compound **3** in CD3OD........20

Figure S21. The DEPT-135 NMR spectrum of compound **3** in CD_3OD........21

Figure S22. The HSQC spectrum of compound **3** in CD_3OD........22

Figure S23. The ^{1}H-^{1}H COSY spectrum of compound **2** in CD_3OD........23

Figure S24. The HMBC spectrum of compound **3** in CD_3OD........24

Figure S26. The (+)-HRESIMS spectroscopic data of compound **3**........26

Figure S27. The IR spectrum of compound **3**........27

Figure S28. The ^{1}H-NMR spectrum of compound **4** in CD_3OD........28

Figure S29. The ^{13}C-NMR spectrum of compound **4** in CD_3OD........29

Figure S30. The DEPT-135 NMR spectrum of compound **4** in CD_3OD 30

Figure S31. The HSQC spectrum of compound **4** in CD_3OD..31

Figure S32. The ^1H-^1H COSY spectrum of compound **4** in CD_3OD 32

Figure S33. The HMBC spectrum of compound **4** in CD_3OD...33

Figure S34. The NOESY spectrum of compound **4** in CD_3OD 34

Figure S35. The (+)-HRESIMS spectroscopic data of compound **4**...35

Figure S36. The IR spectrum of compound **4**...36

S37. The ECD calculation data..37

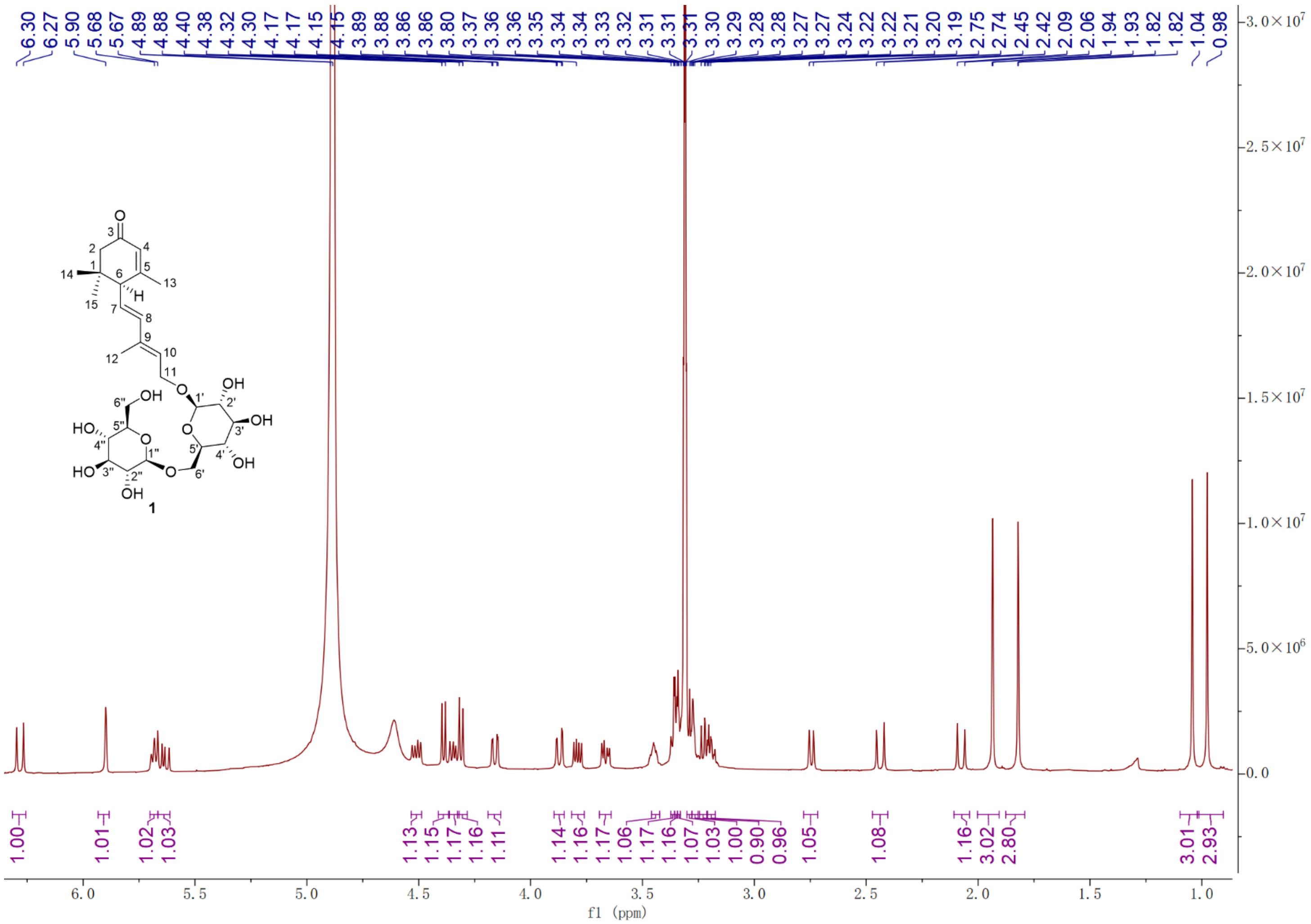

Figure S1. The ^{1}H-NMR spectrum of compound **1** in CD_3OD

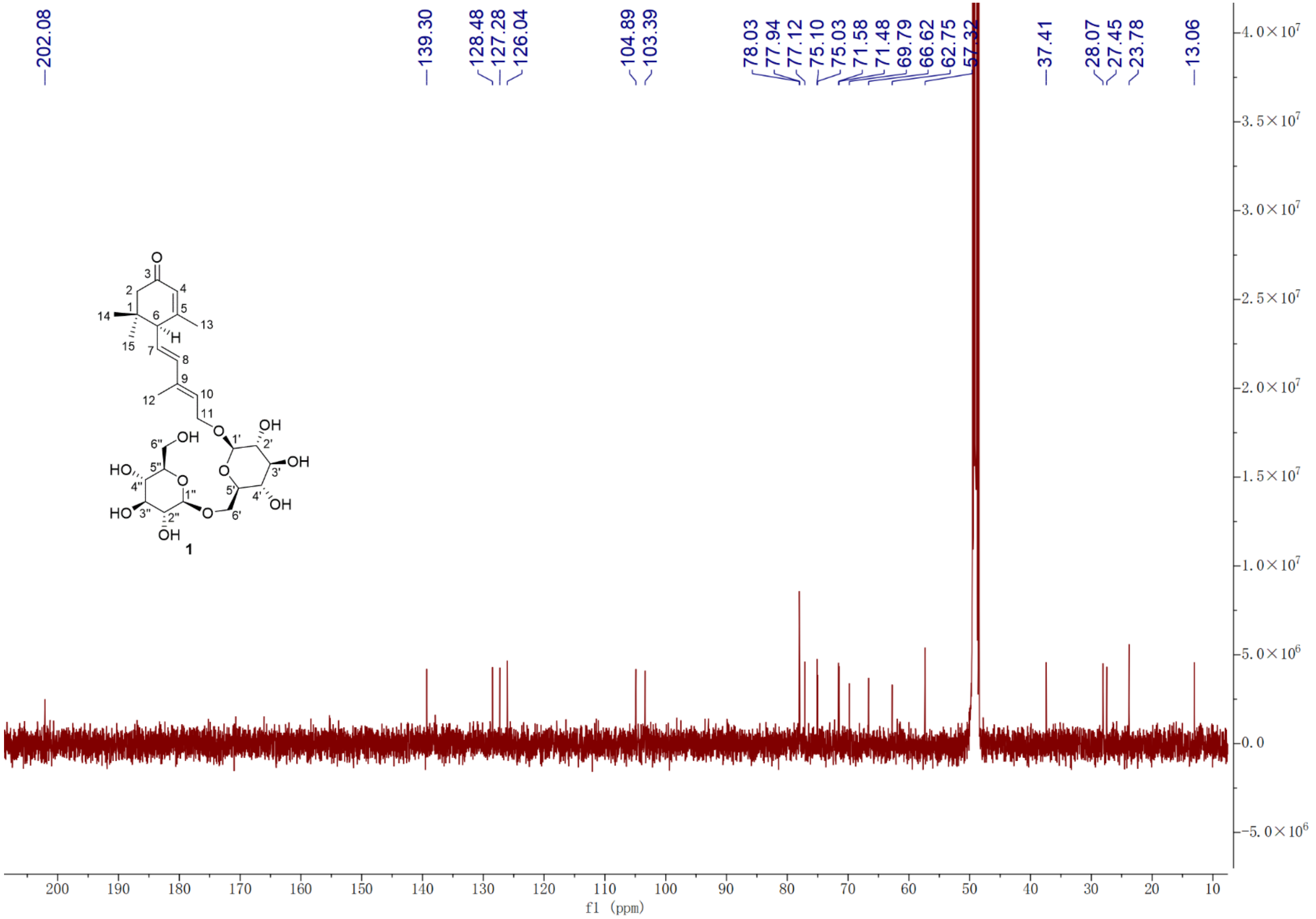

Figure S2. The ^{13}C-NMR spectrum of compound **1** in CD_3OD

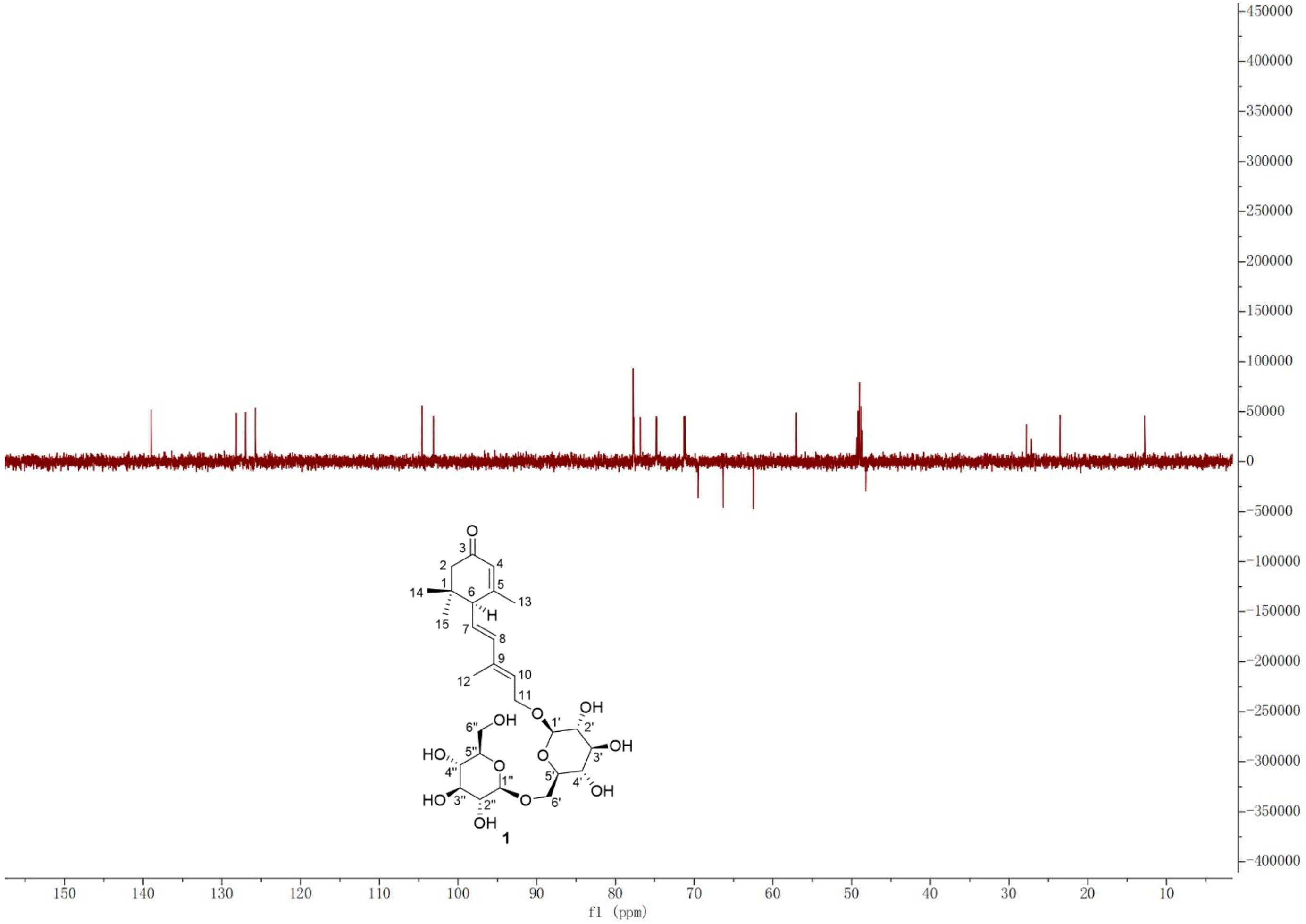

Figure S3. The DEPT-135 NMR spectrum of compound **1** in CD_3OD

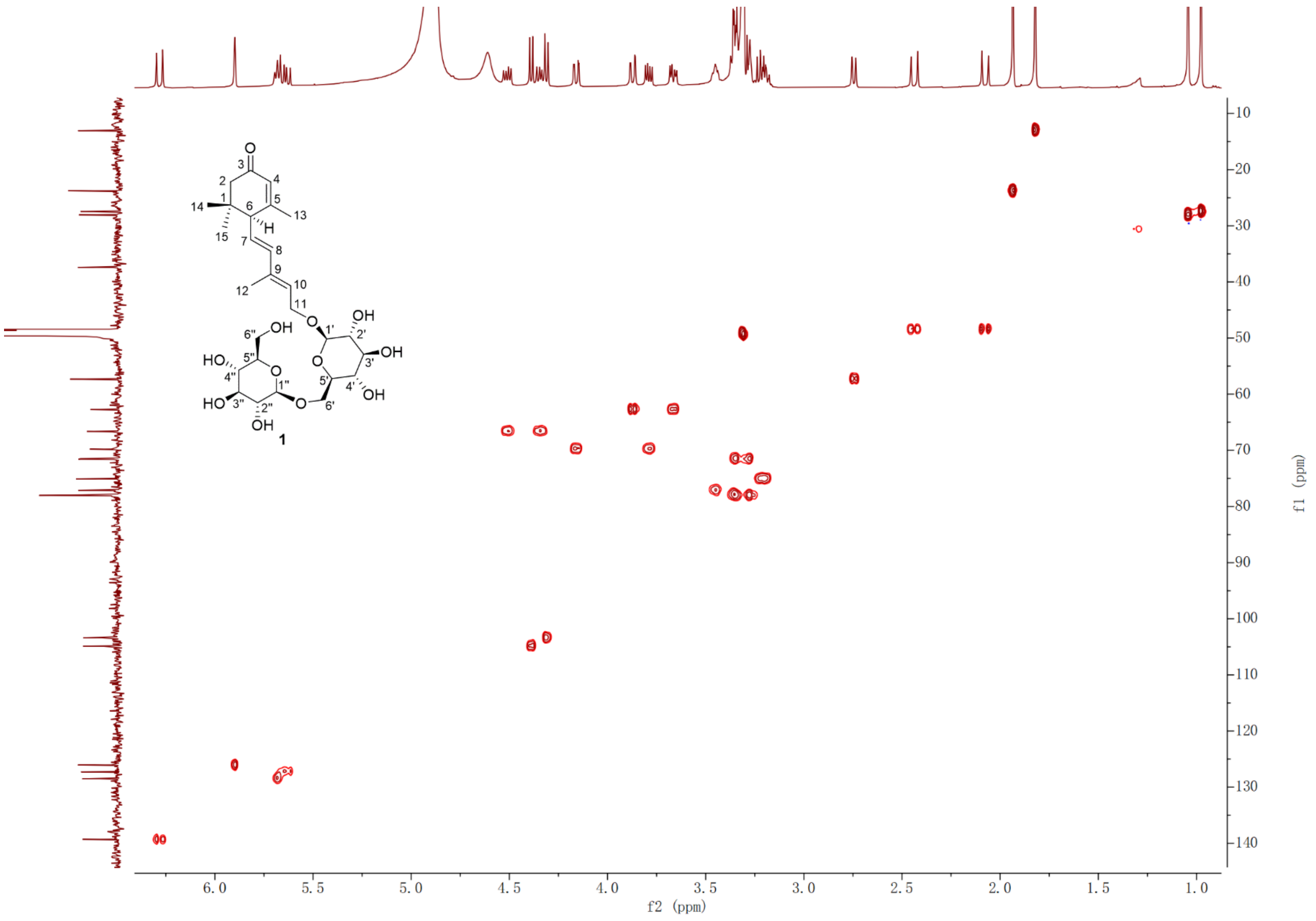

Figure S4. The HSQC spectrum of compound **1** in CD_3OD

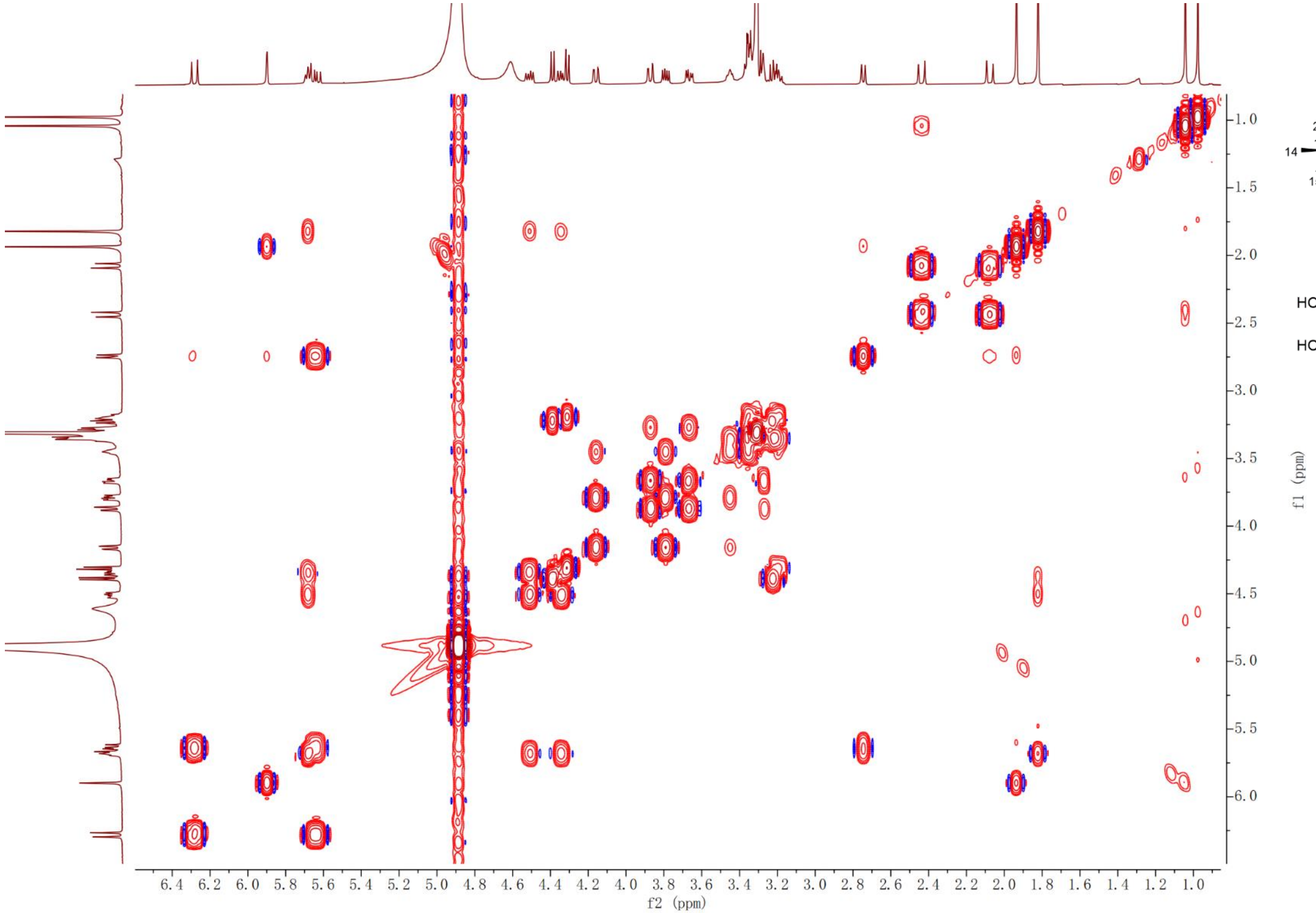

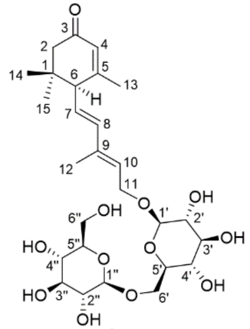

1

Figure S5. The ^{1}H-^{1}H COSY spectrum of compound **1** in CD_3OD

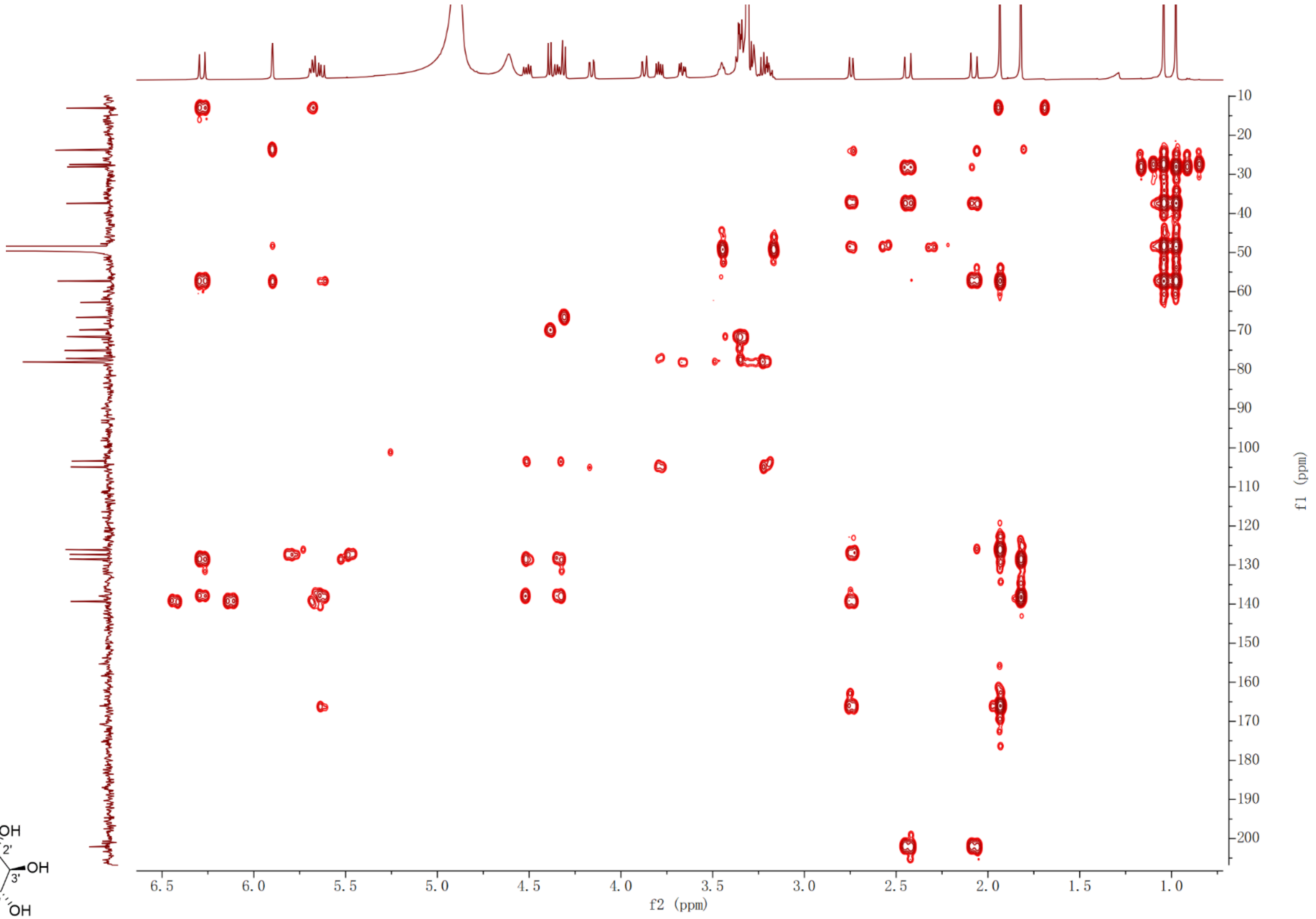

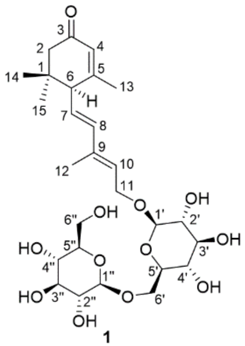
1

Figure S6. The HMBC spectrum of compound **1** in CD_3OD

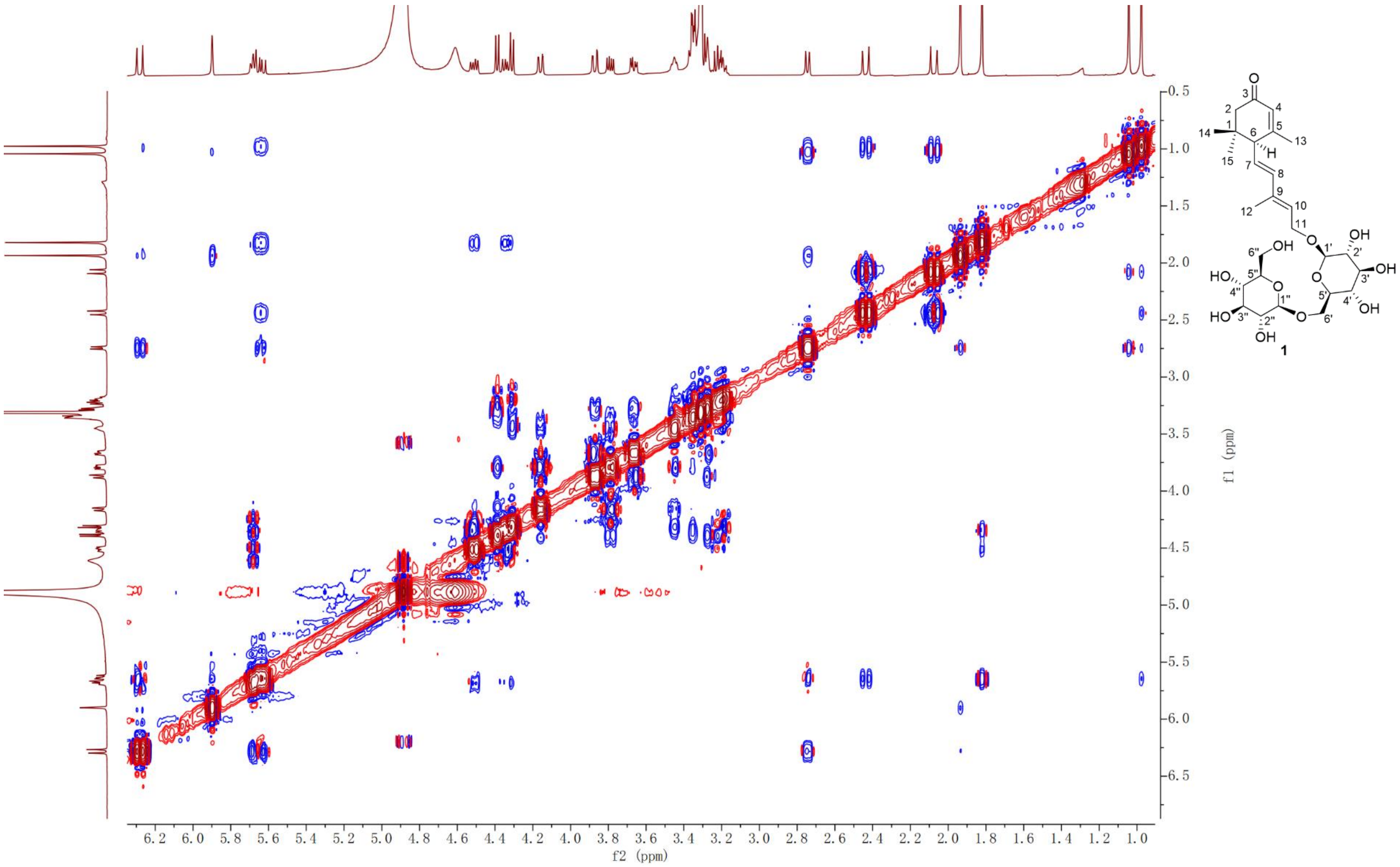

Figure S7. The NOESY spectrum of compound **1** in CD_3OD

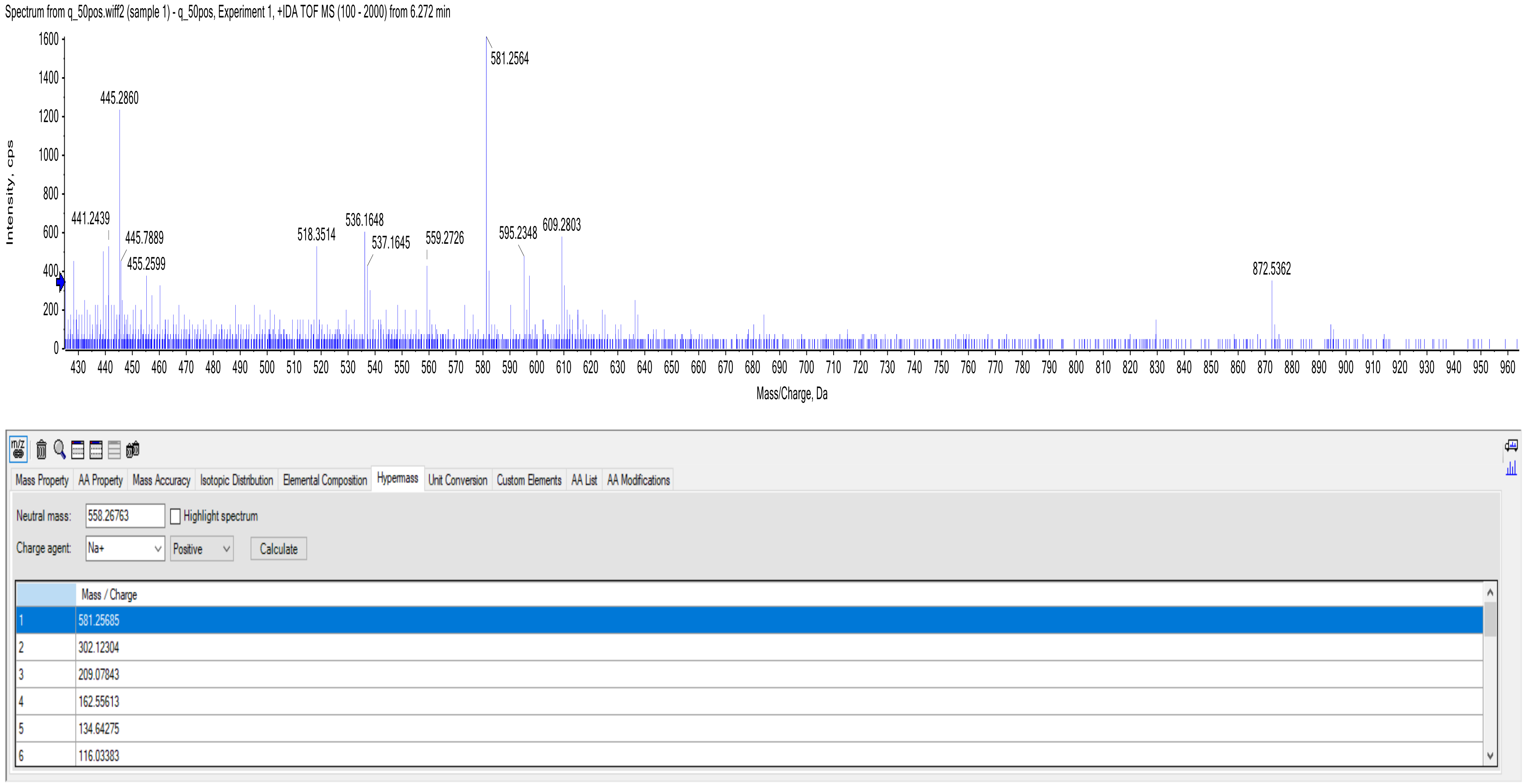

Figure S8. The (+)-HRESIMS spectroscopic data of compound **1**

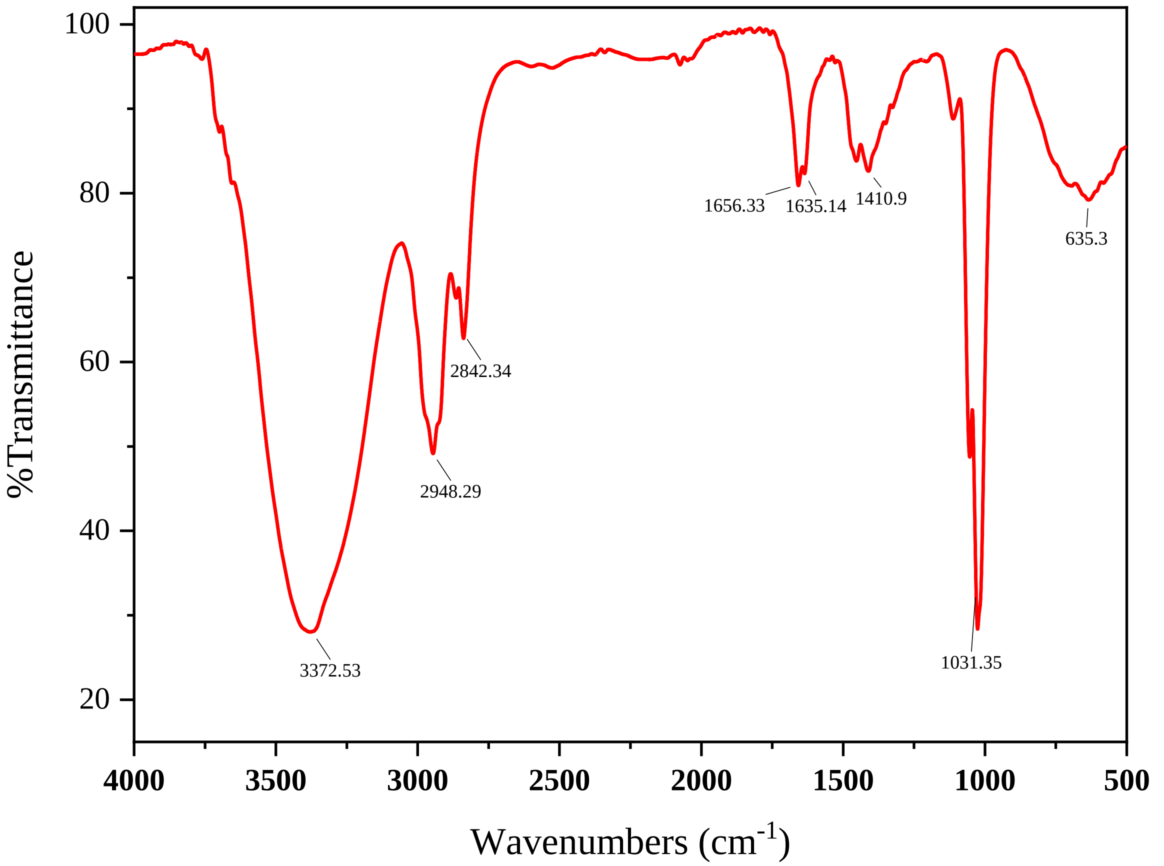

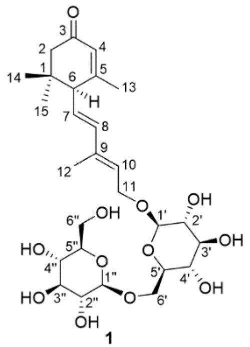
1

Figure S9. The IR spectrum of compound **1**

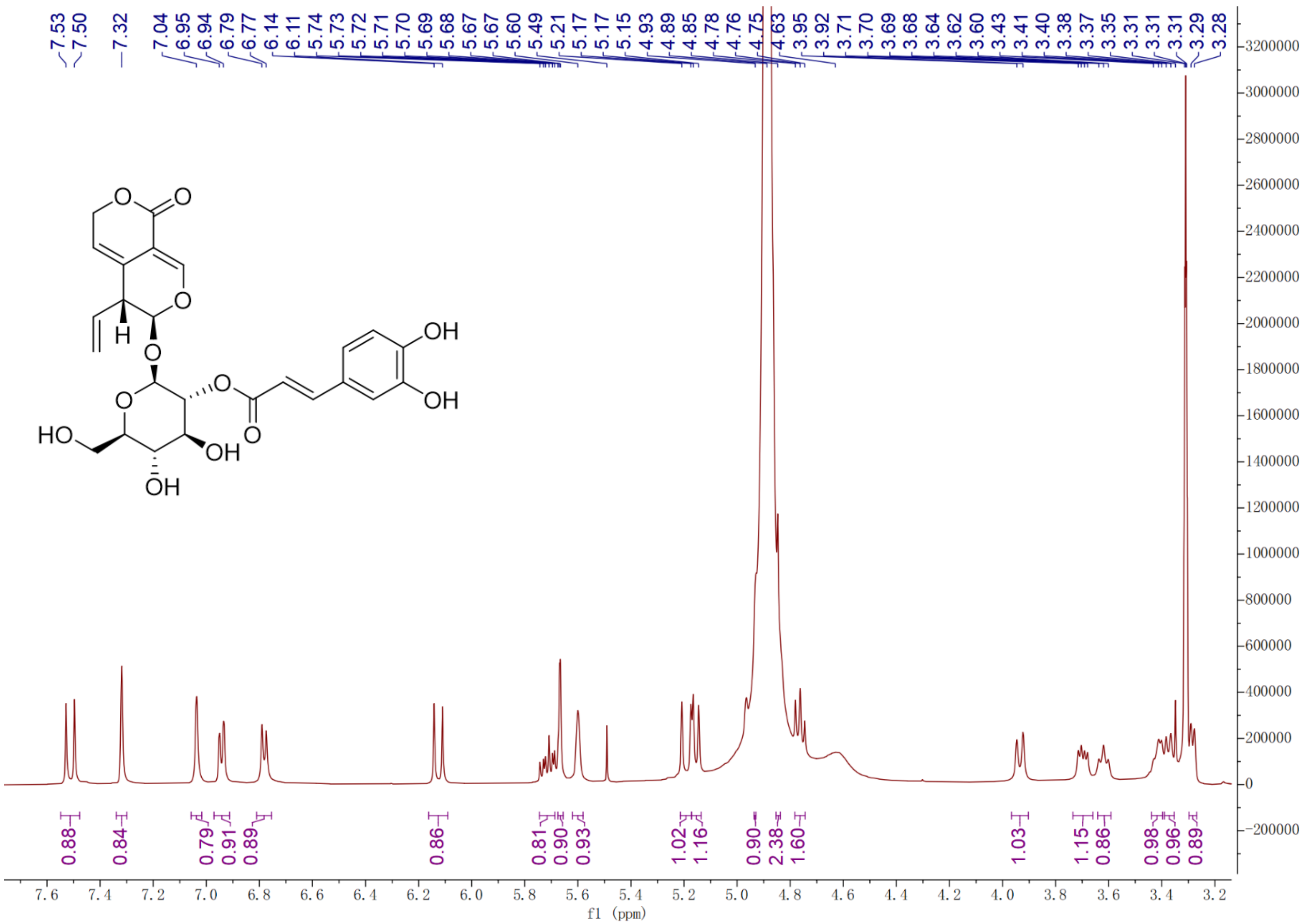

Figure S10. The ^{1}H-NMR spectrum of compound **2** in CD_3OD

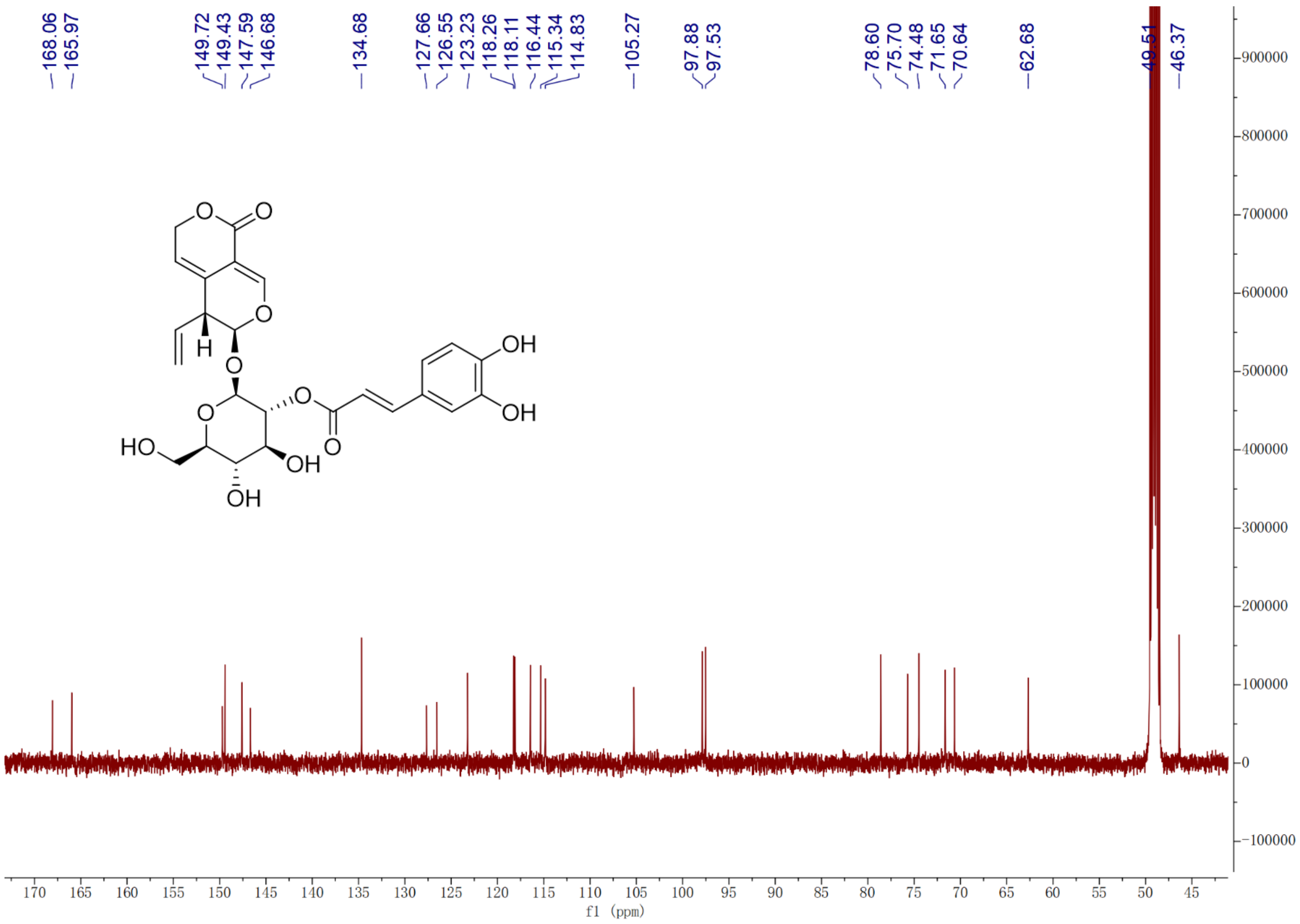

Figure S11. The ^{13}C-NMR spectrum of compound **2** in CD_3OD

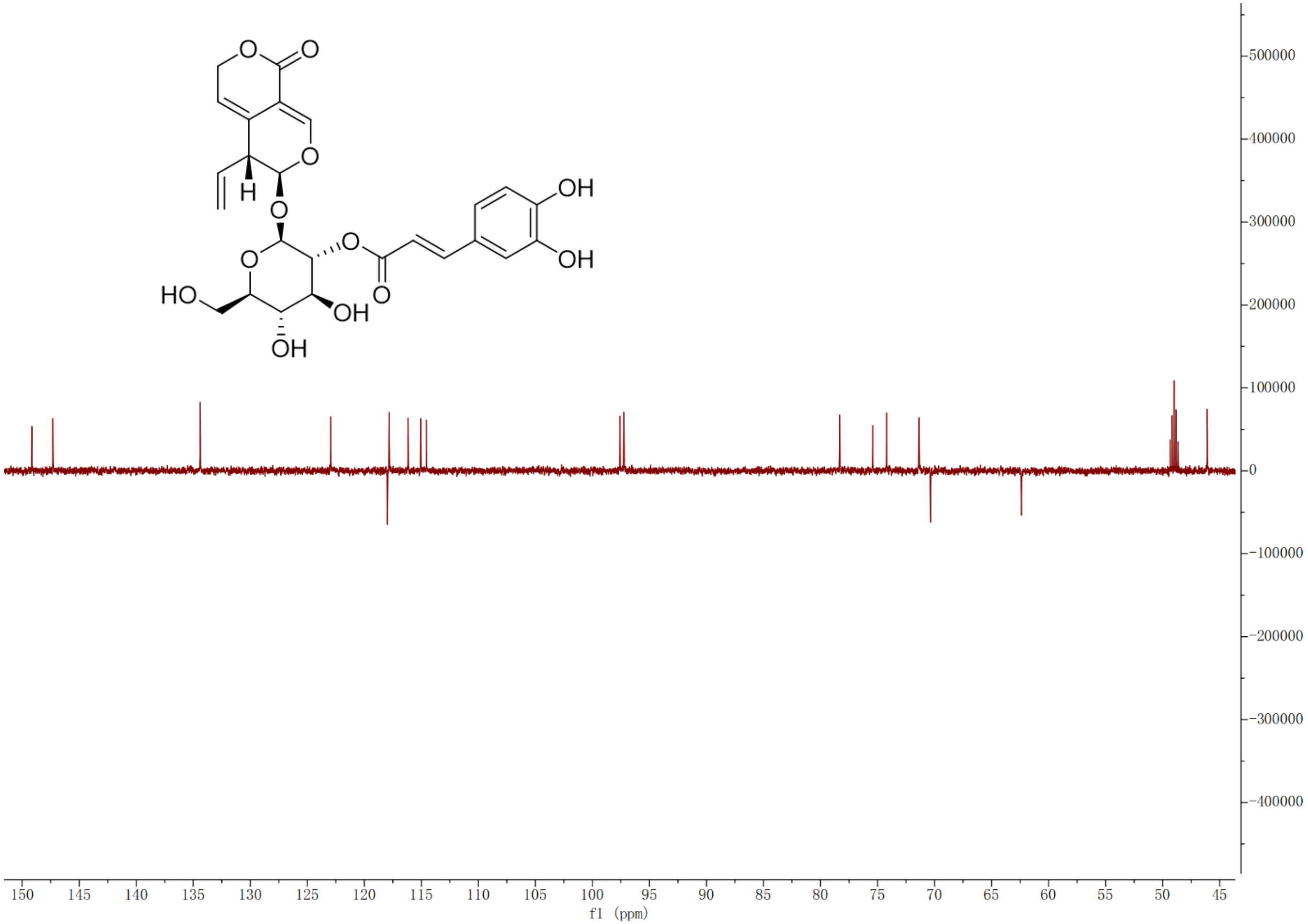

Figure S12. The DEPT-135 NMR spectrum of compound **2** in CD_3OD

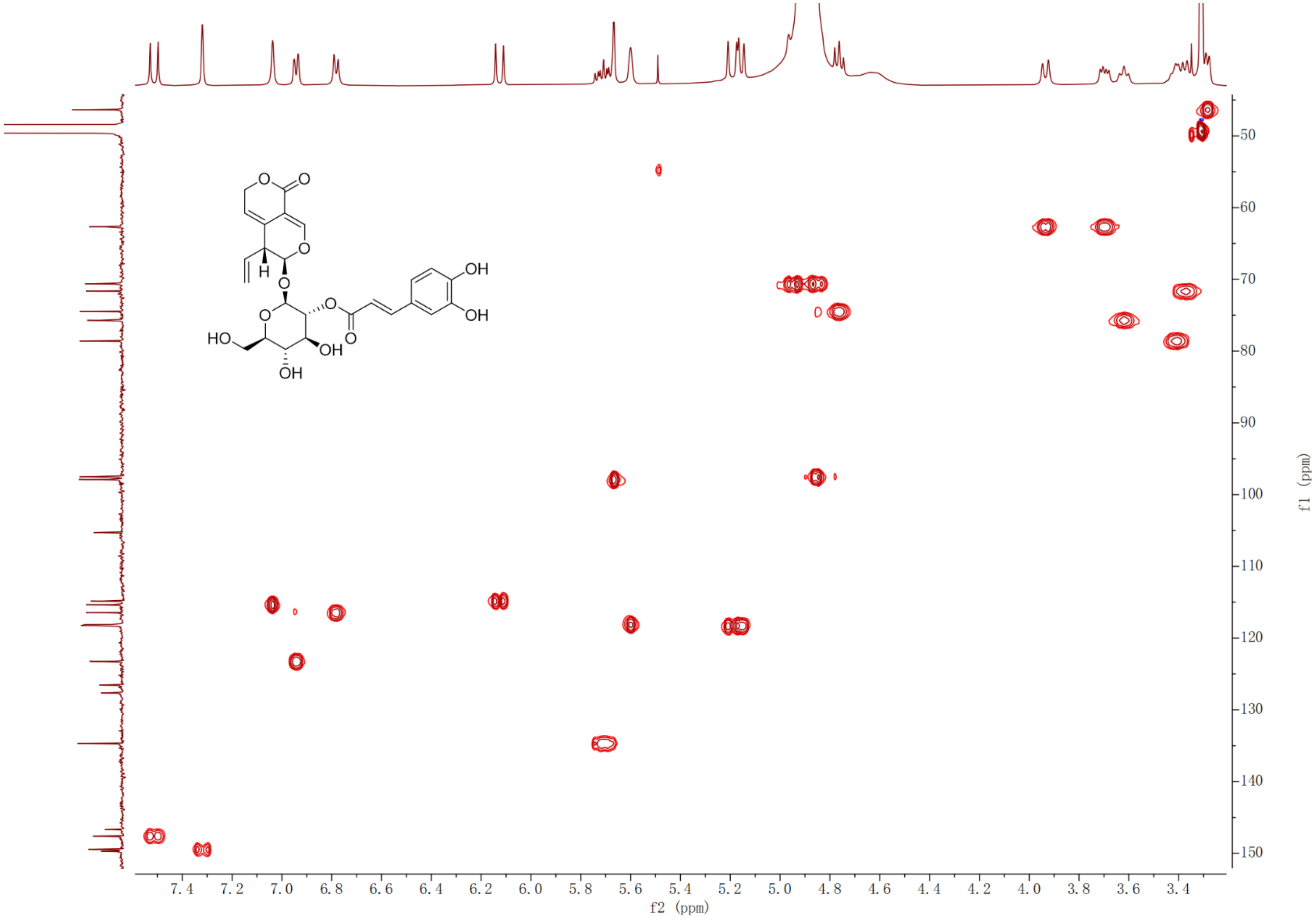

Figure S13. The HSQC spectrum of compound **2** in CD_3OD

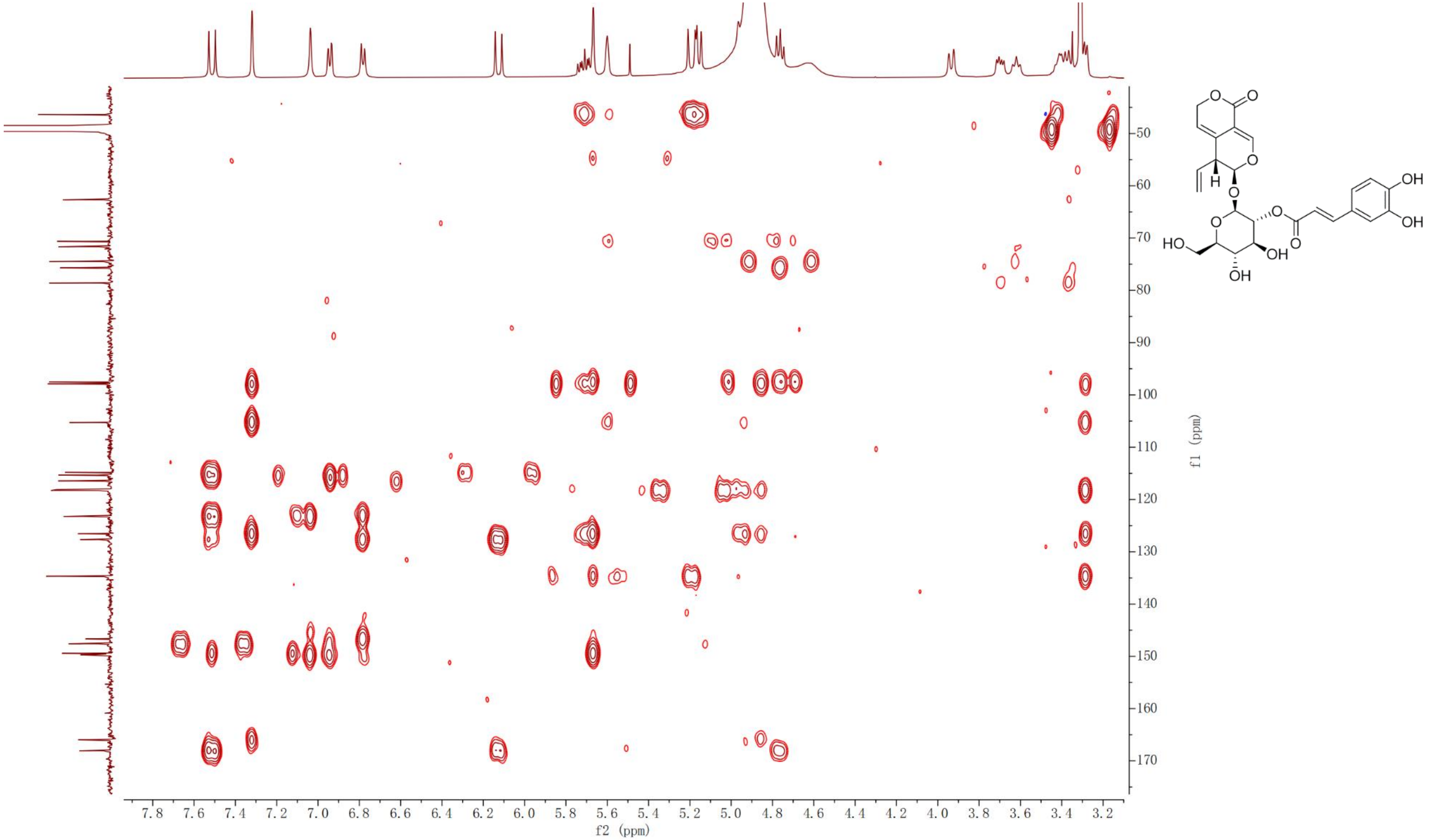

Figure S14. The ^{1}H-^{1}H COSY spectrum of compound **2** in CD_3OD

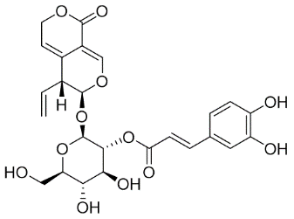

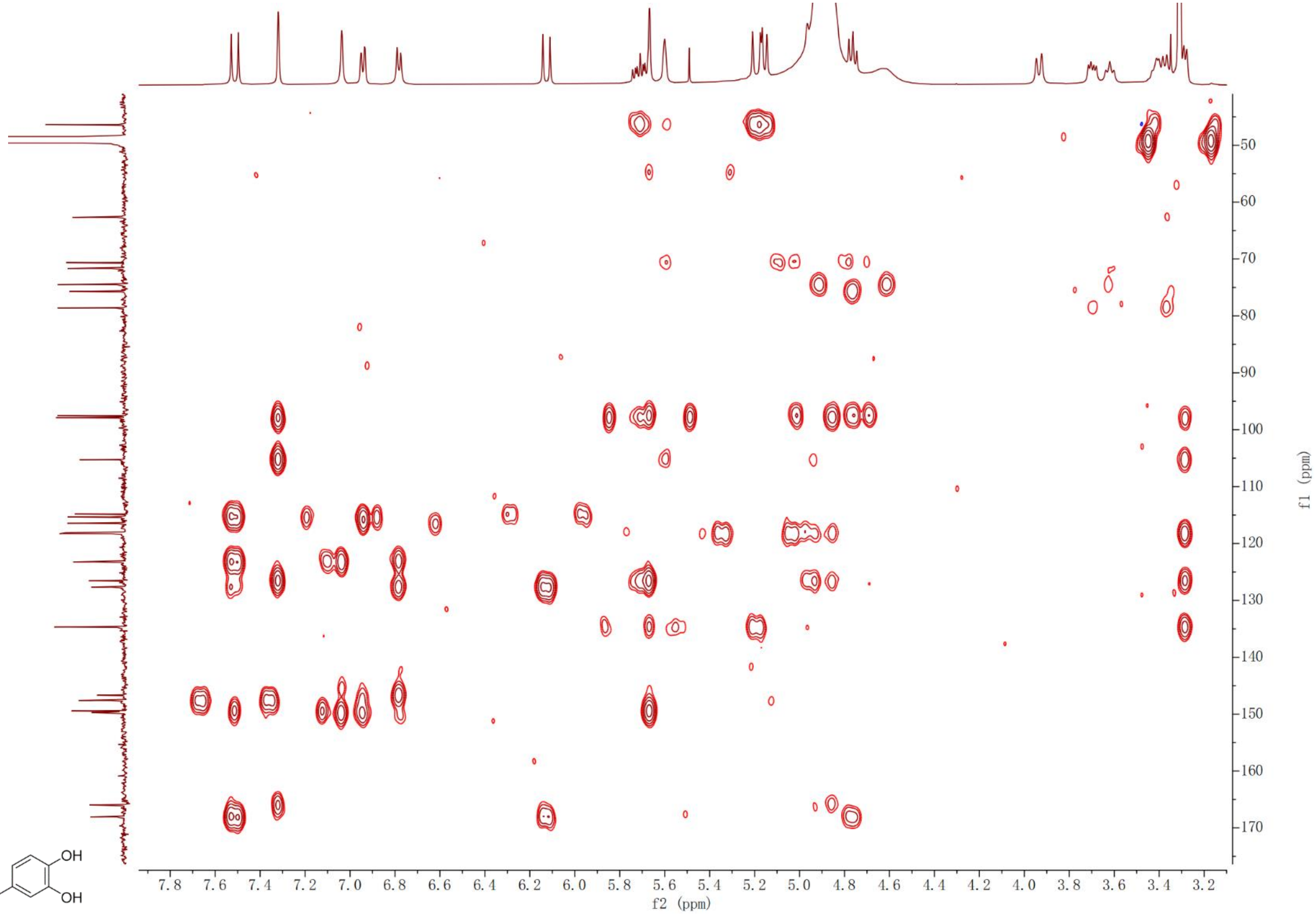

Figure S15. The HMBC spectrum of compound **2** in CD_3OD

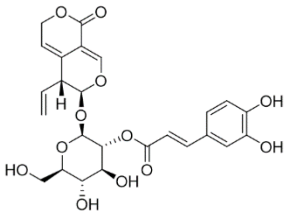

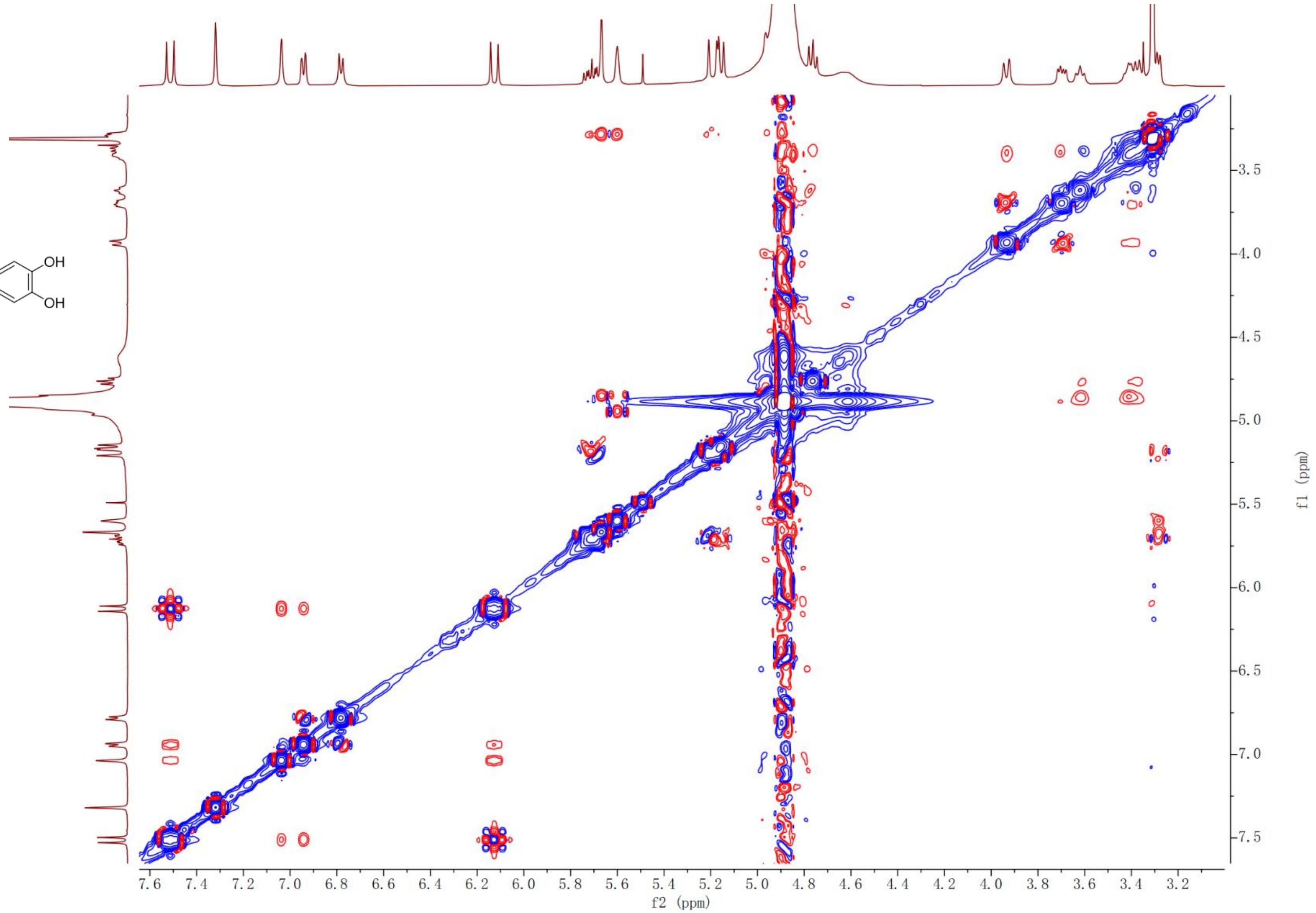

Figure S16. The NOESY spectrum of compound **2** in CD_3OD

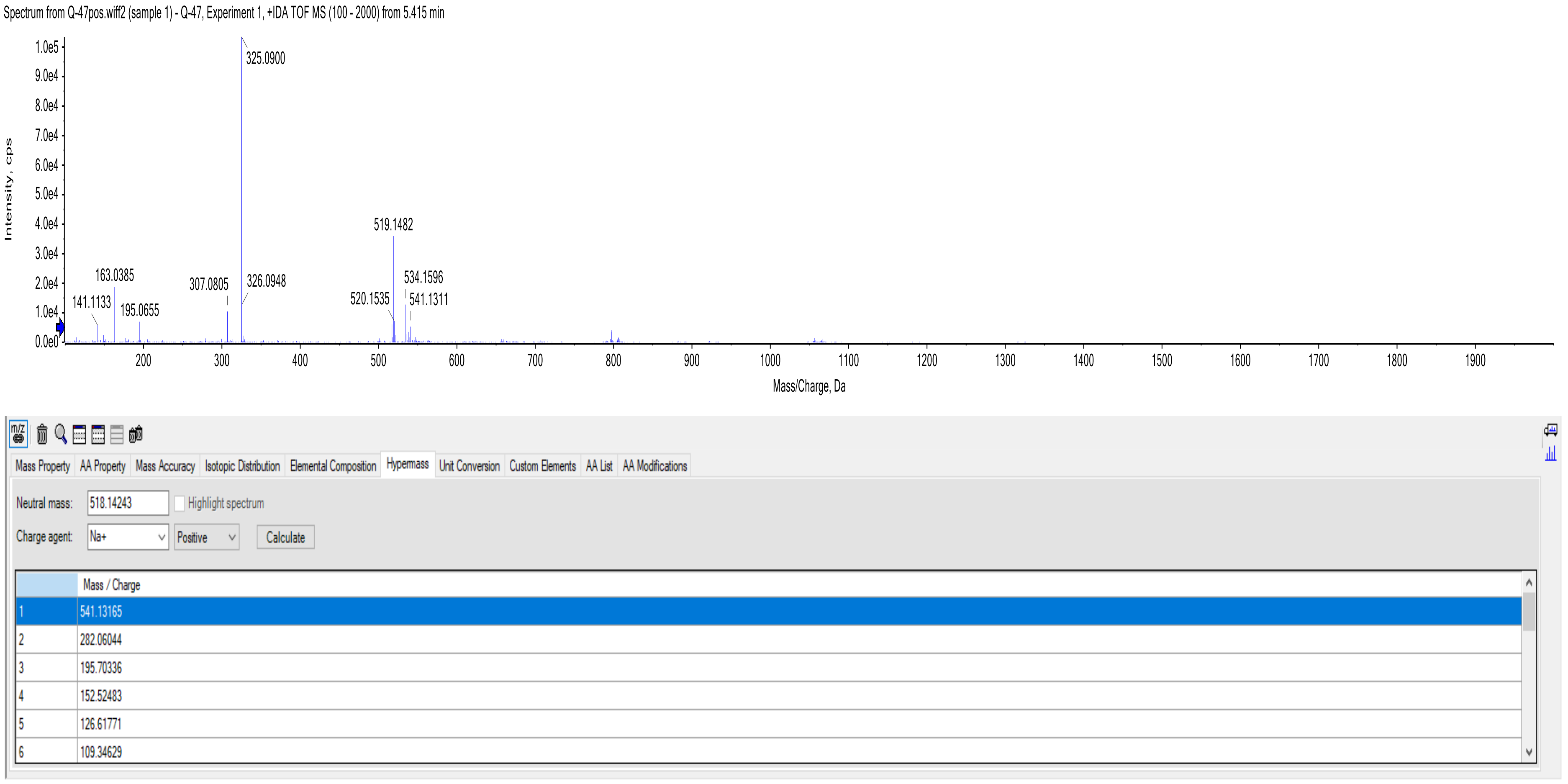

Figure S17. The (+)-HRESIMS spectroscopic data of compound **2**

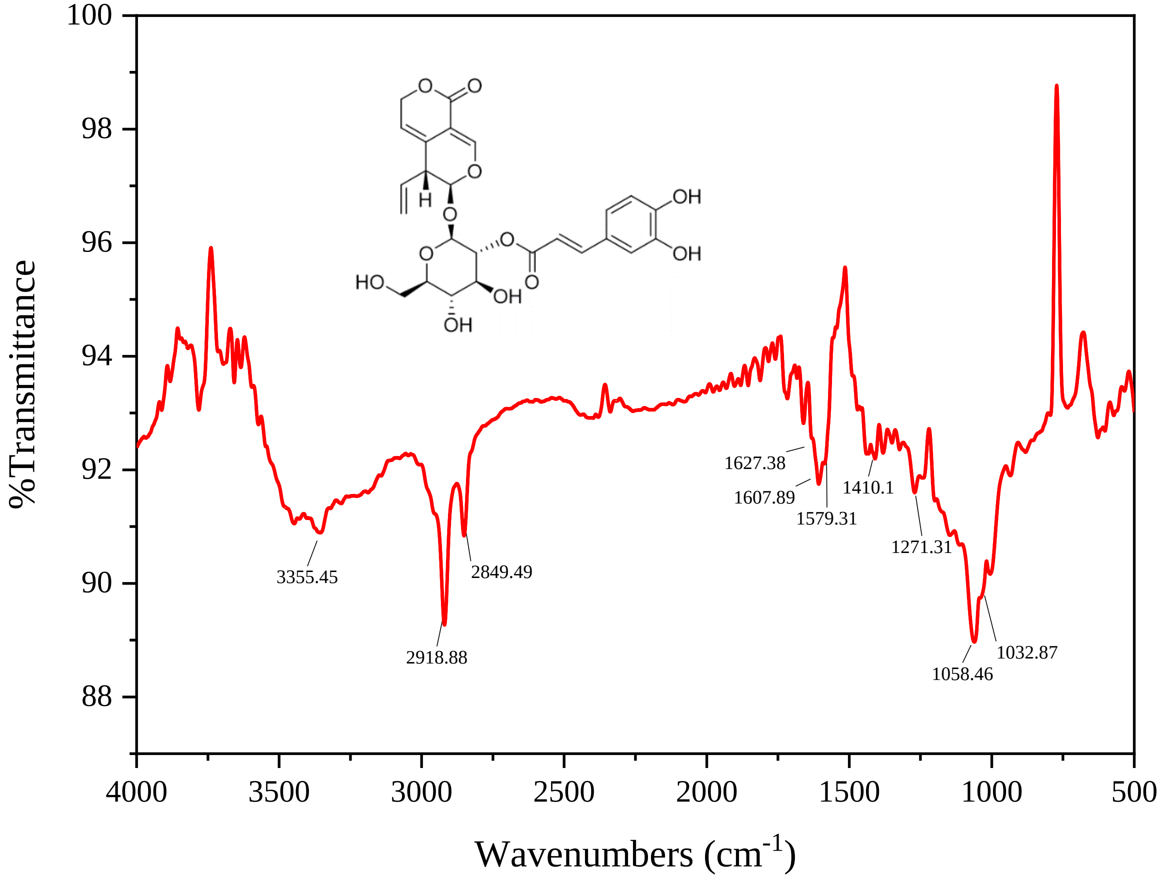

Figure S18. The IR spectrum of compound **2**

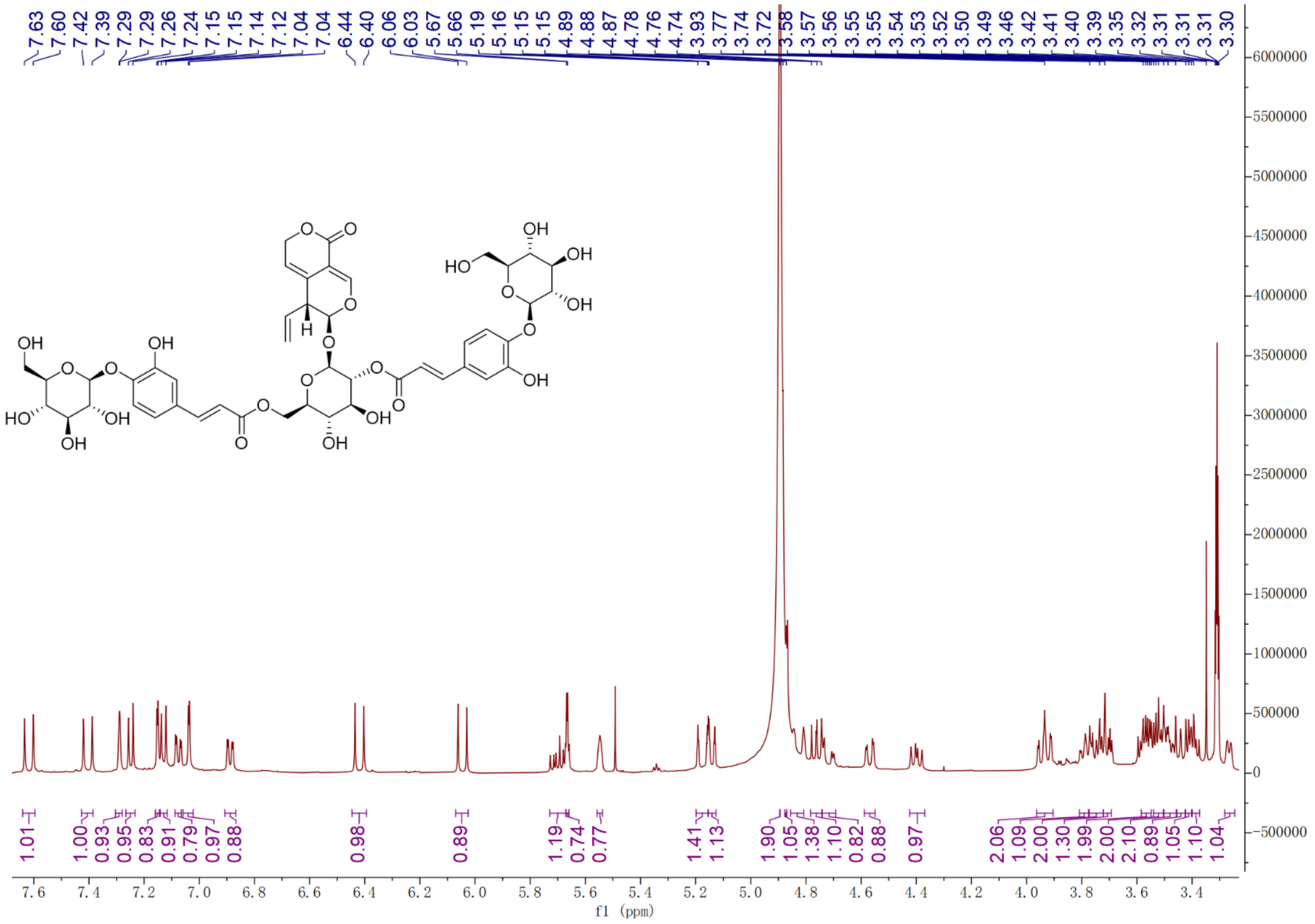

Figure S19. The ^{1}H-NMR spectrum of compound **3** in CD_3OD

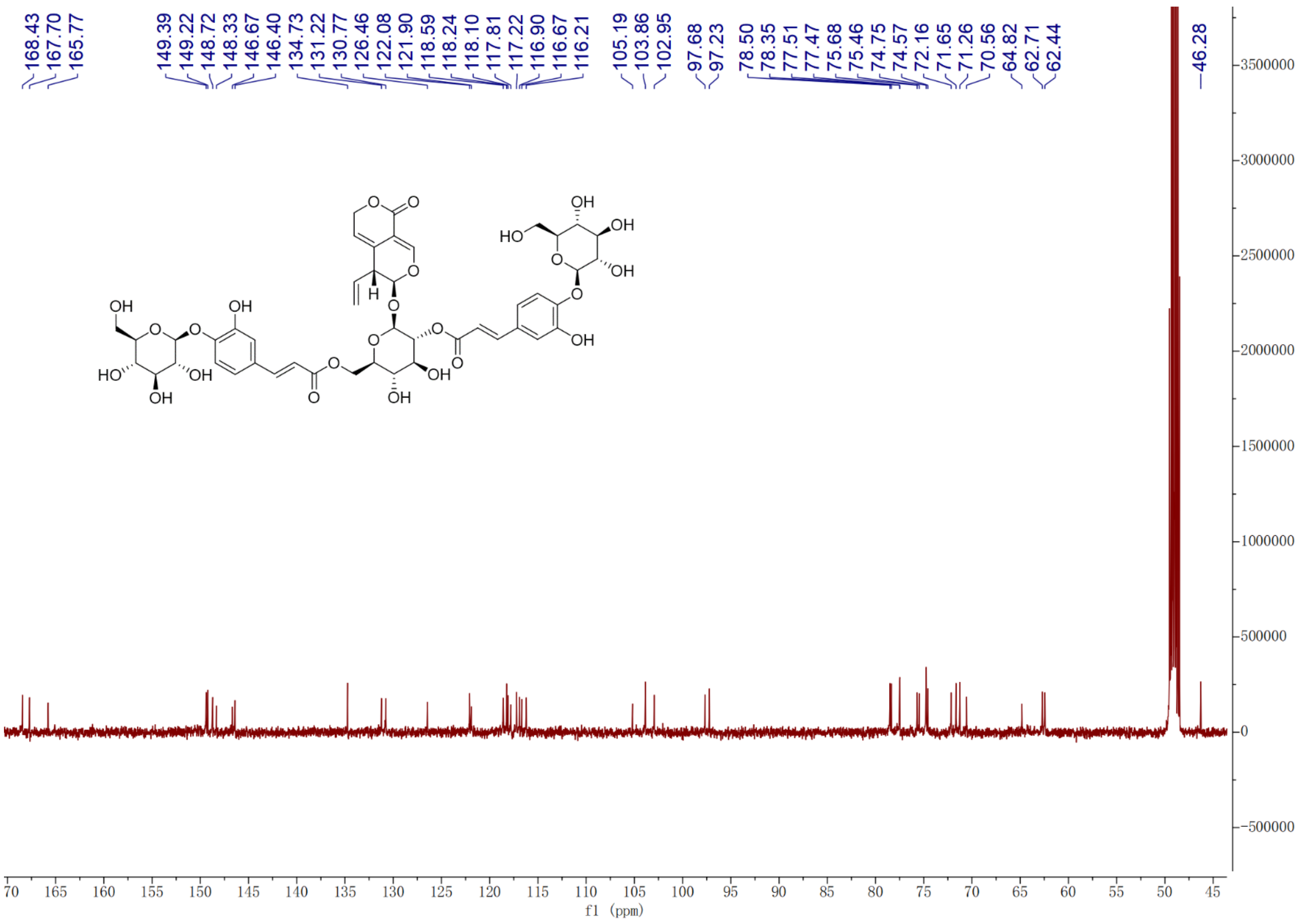

Figure S20. The 13C-NMR spectrum of compound **3** in CD3OD

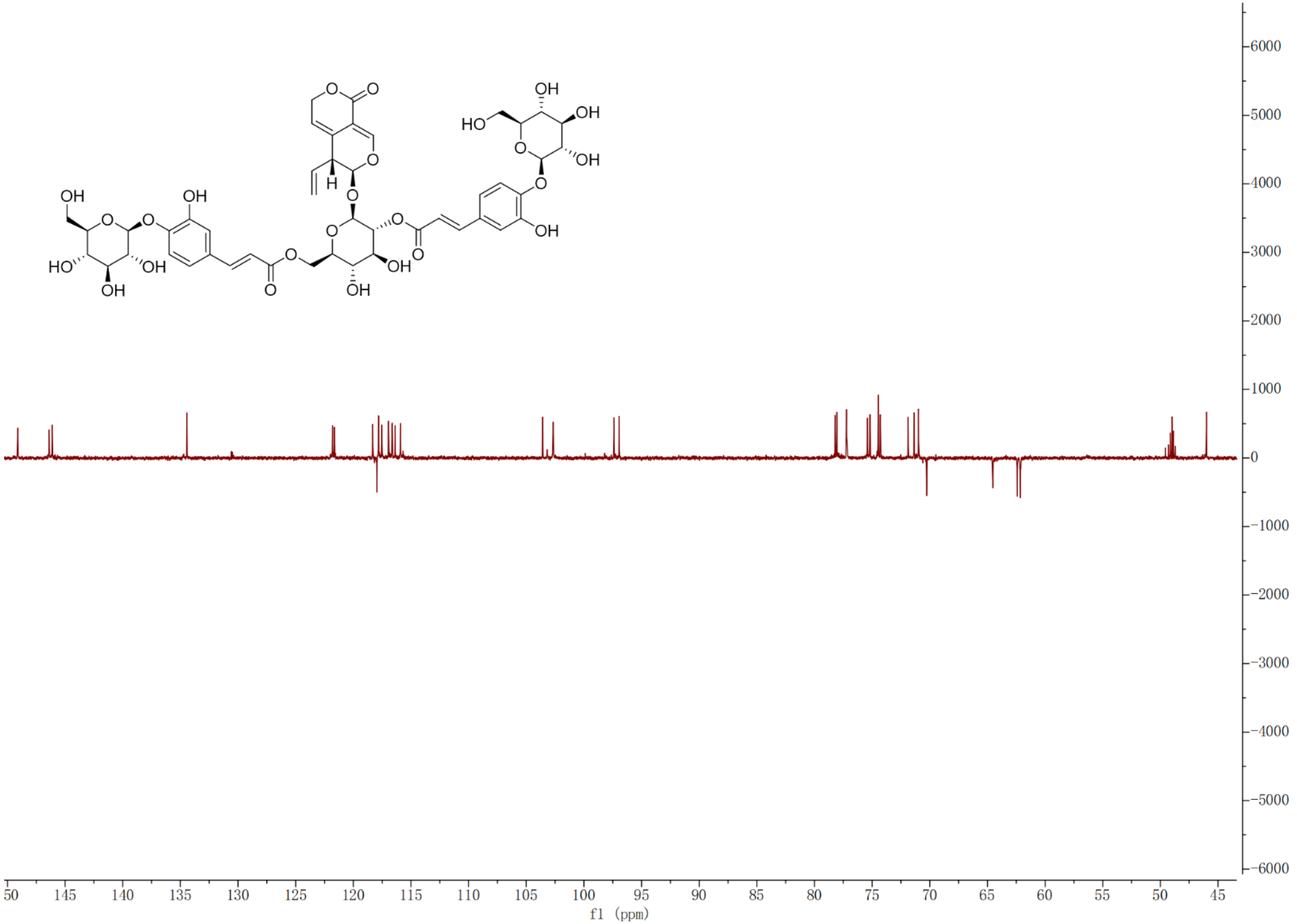

Figure S21. The DEPT-135 NMR spectrum of compound **3** in CD_3OD

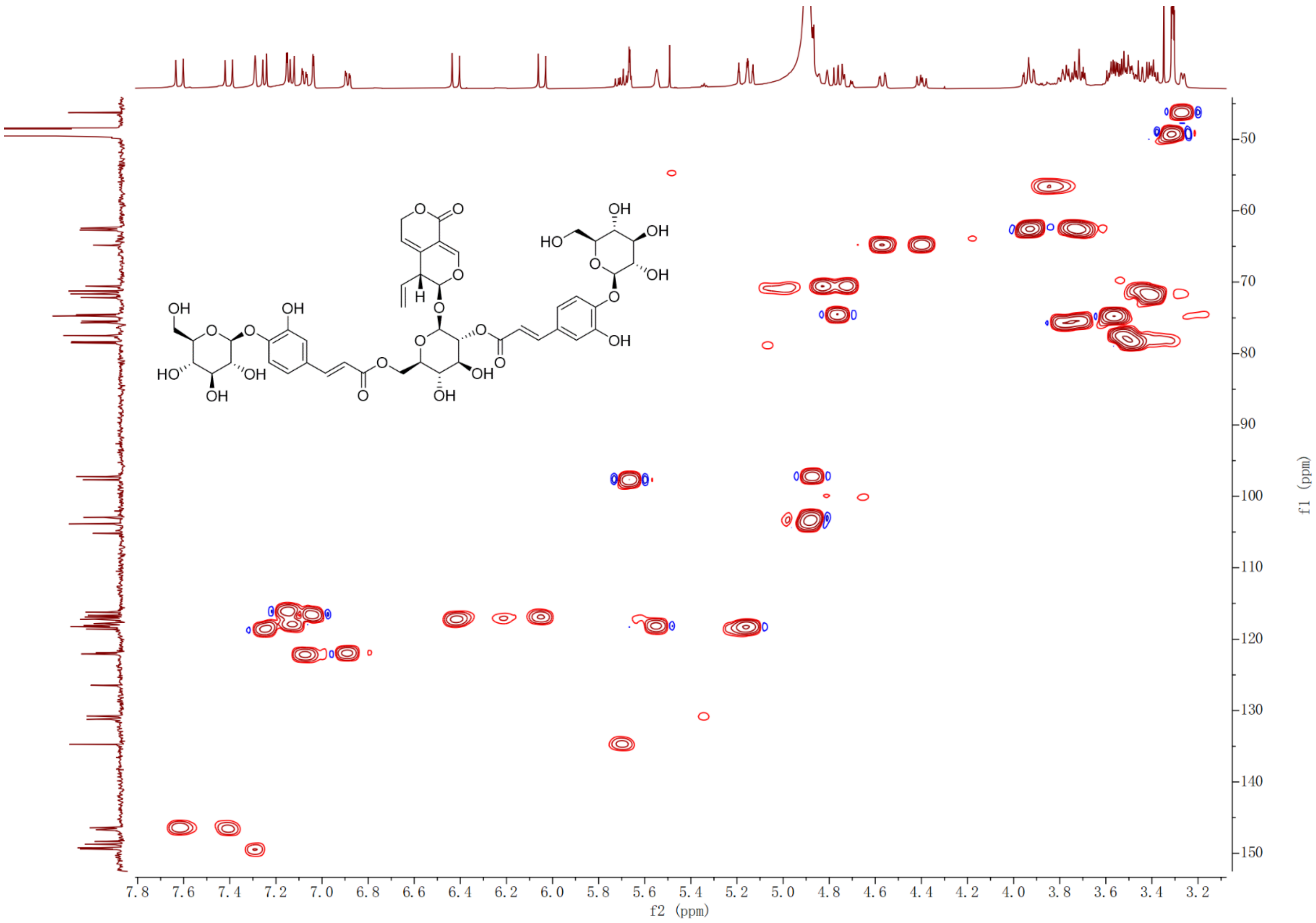

Figure S22. The HSQC spectrum of compound **3** in CD_3OD

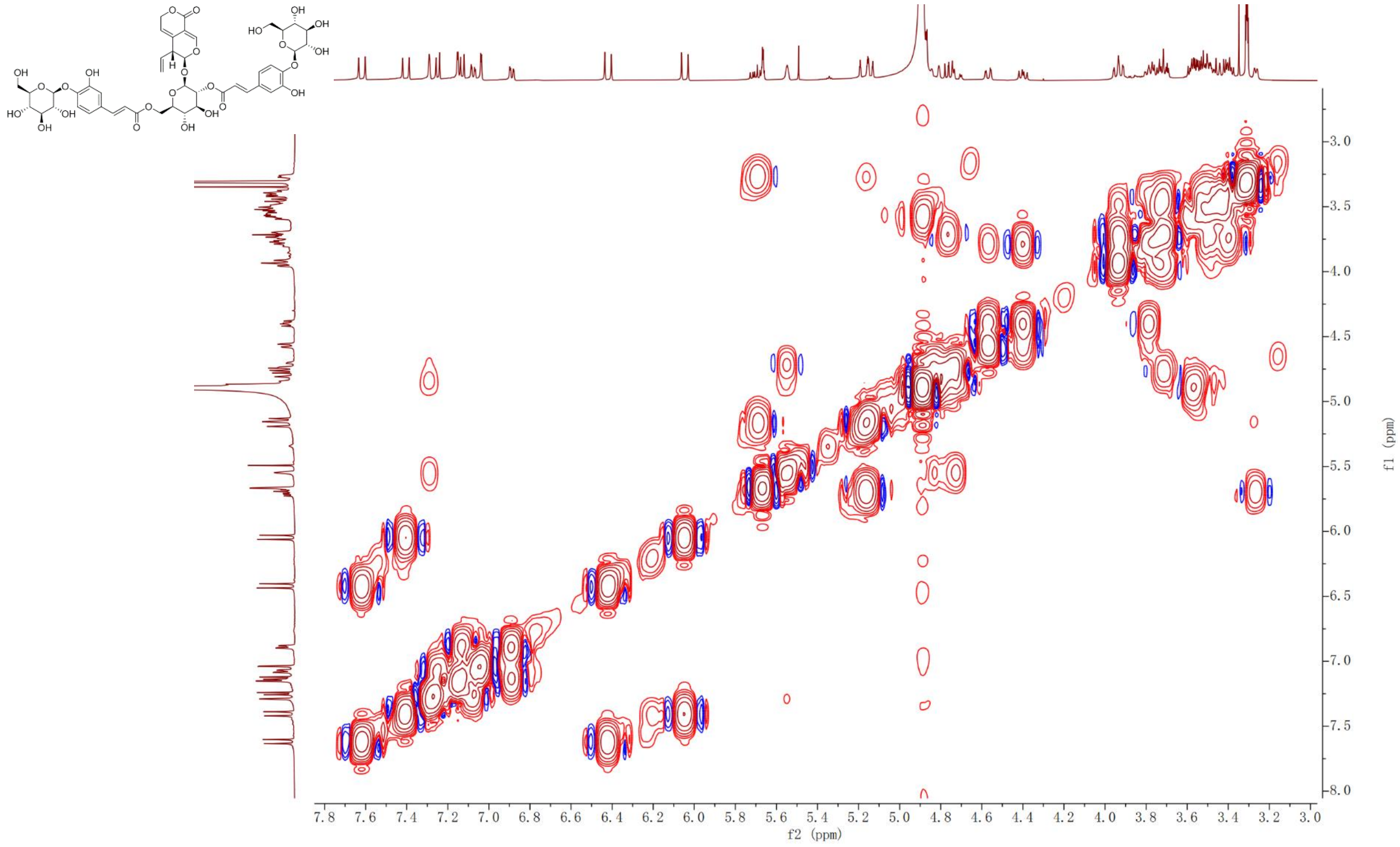

Figure S23. The ^{1}H-^{1}H COSY spectrum of compound **2** in CD_3OD

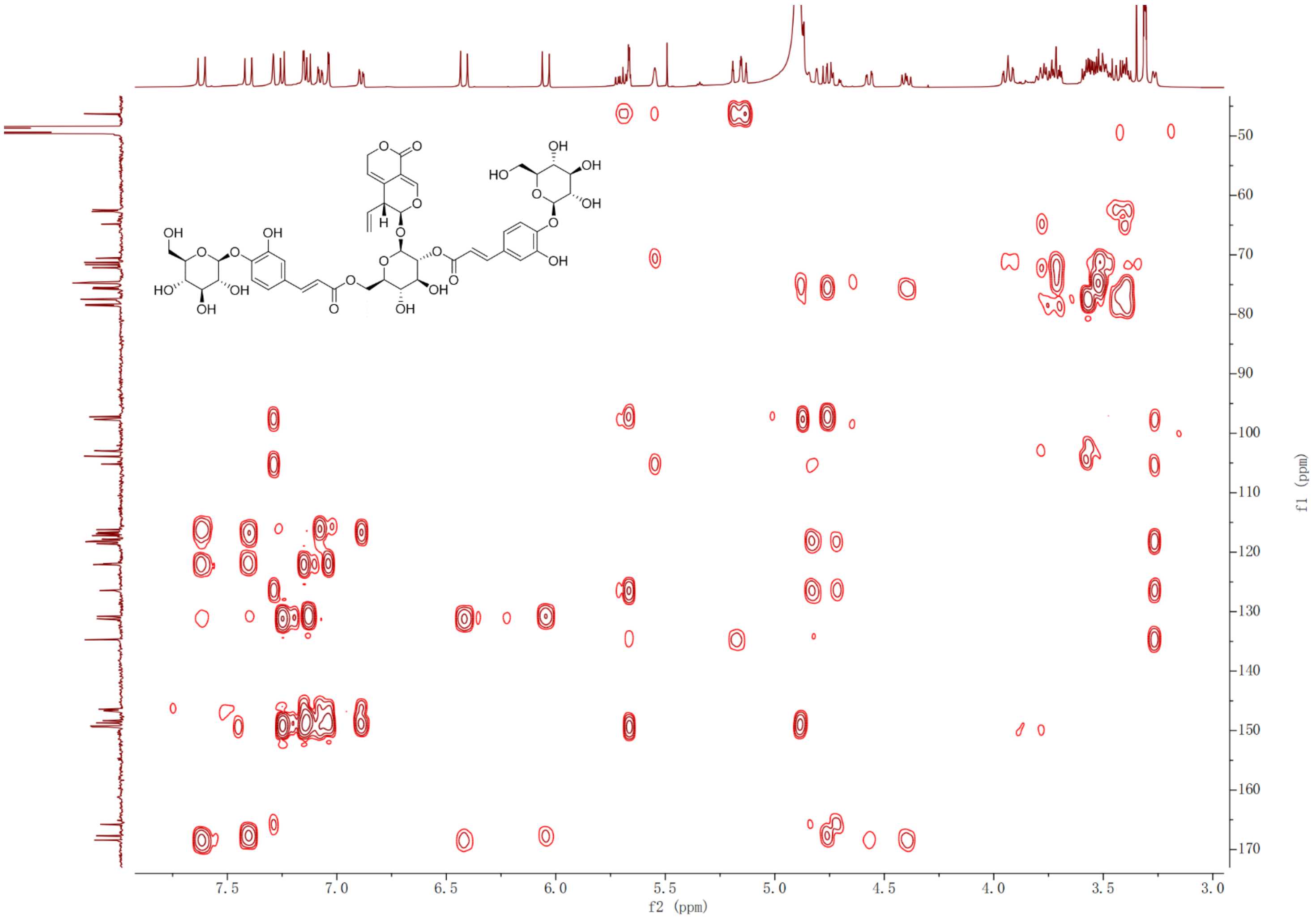

Figure S24. The HMBC spectrum of compound **3** in CD_3OD

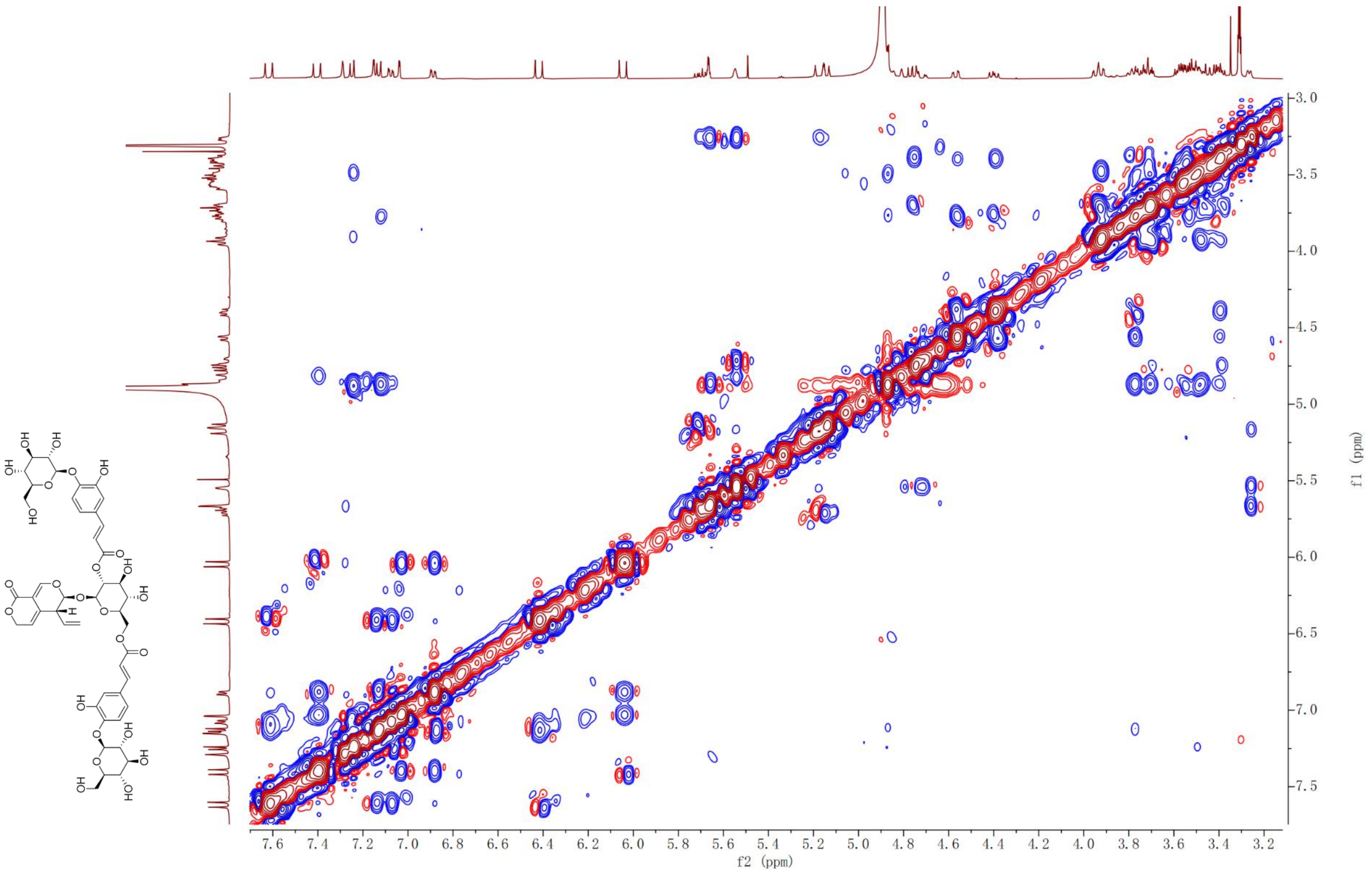

Figure S25. The NOESY spectrum of compound **3** in CD_3OD

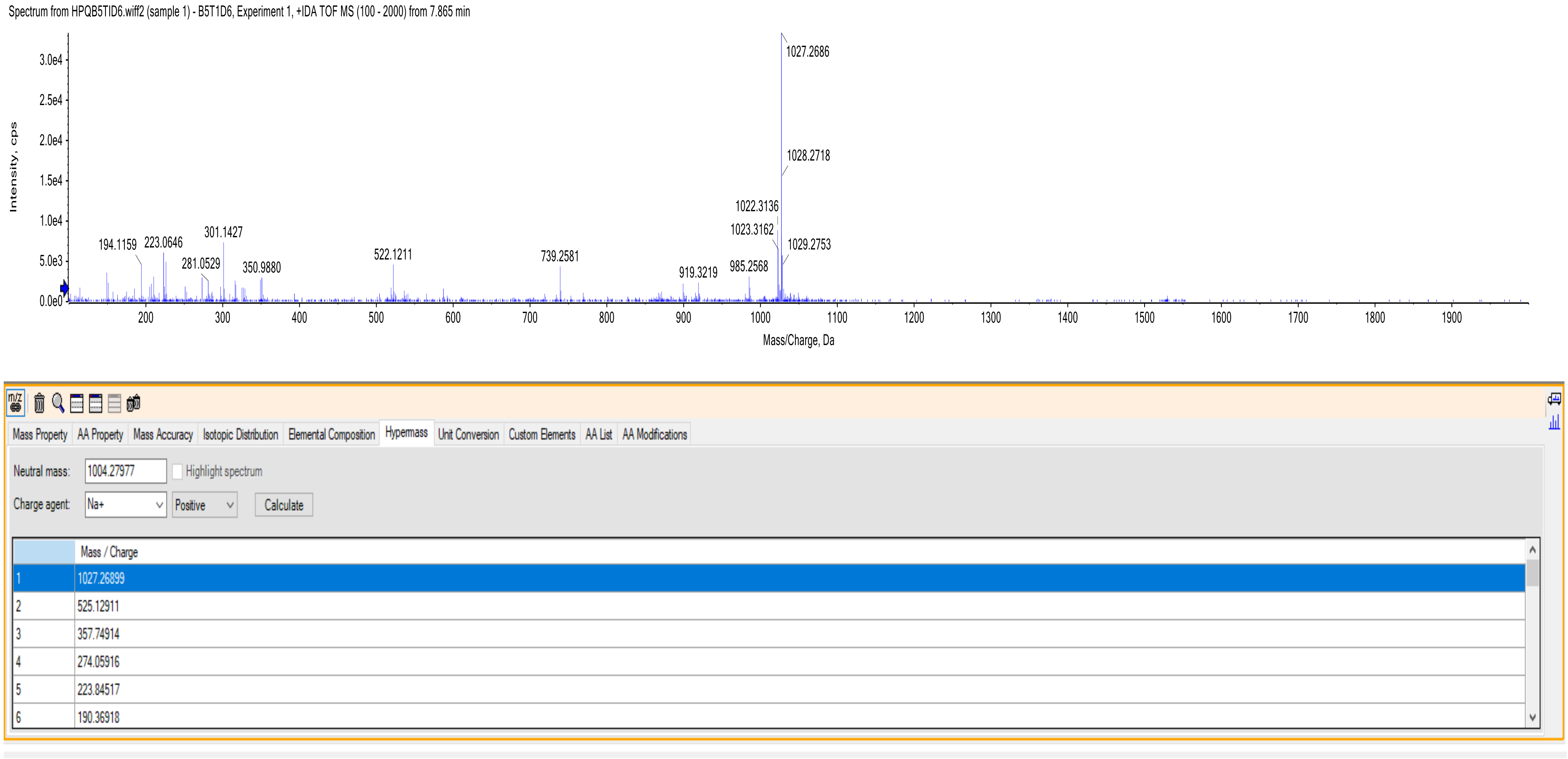

Figure S26. The (+)-HRESIMS spectroscopic data of compound **3**

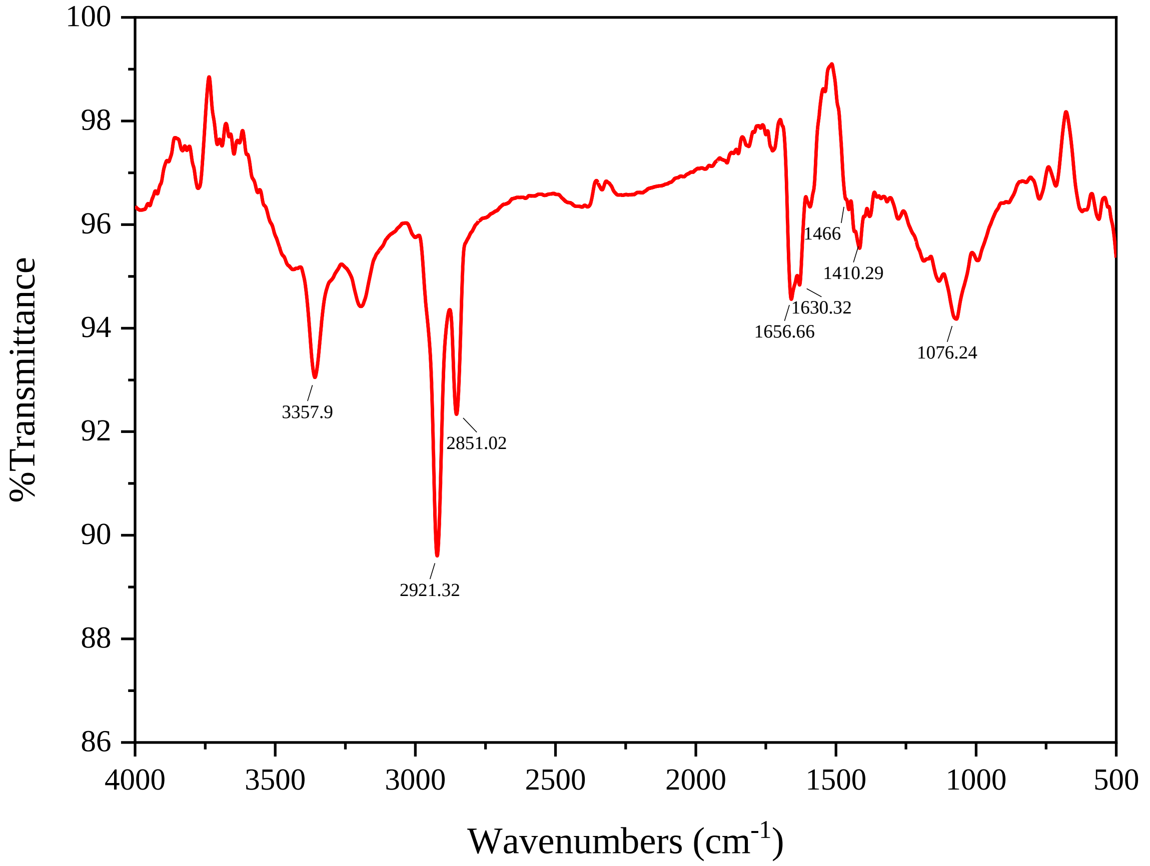

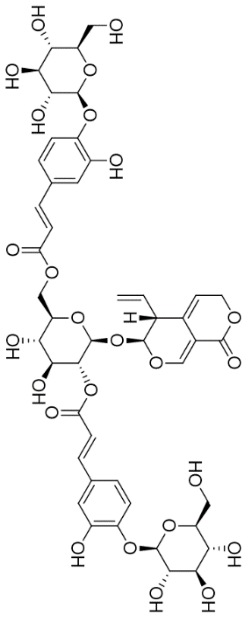

Figure S27. The IR spectrum of compound **3**

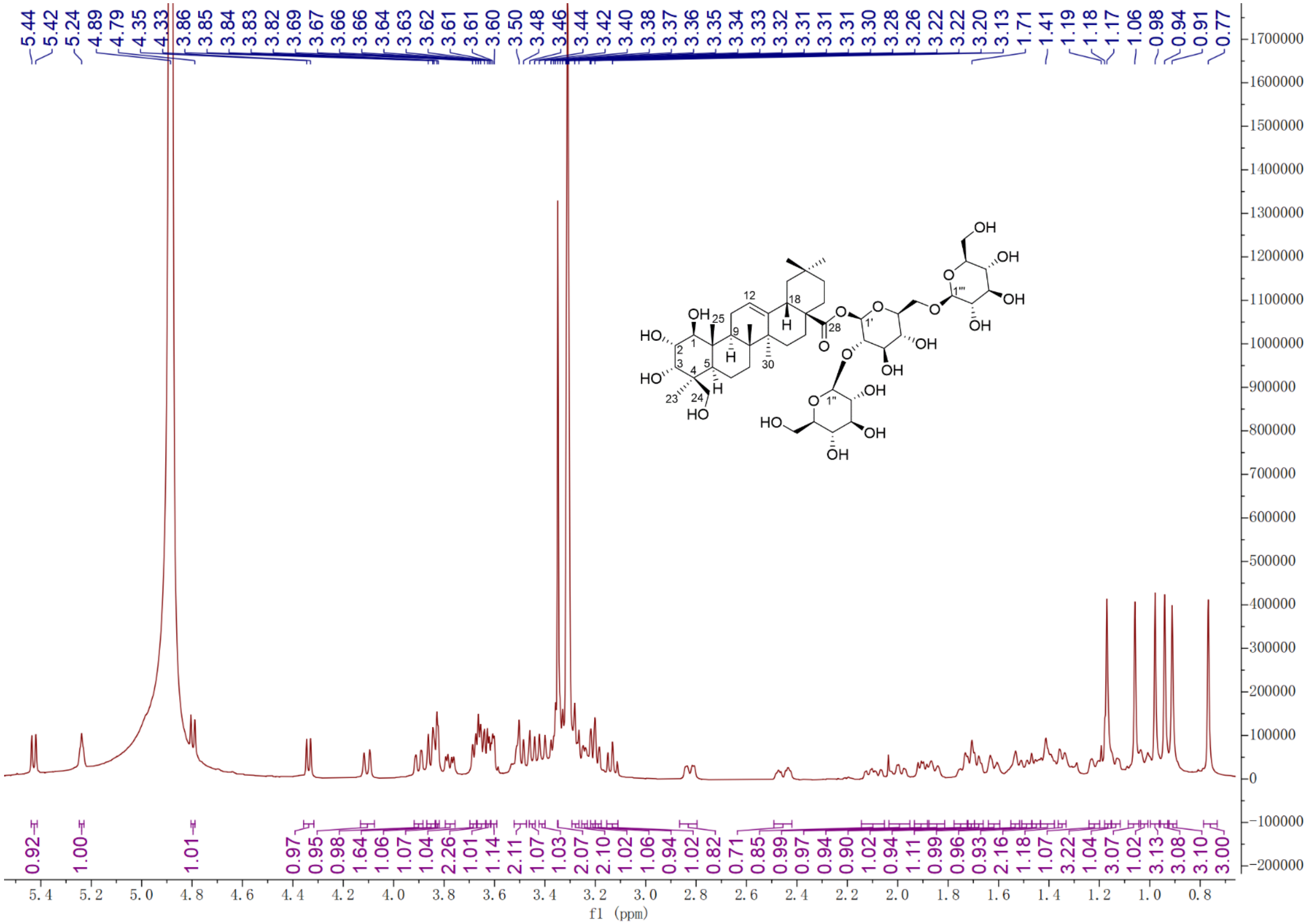

Figure S28. The ^{1}H-NMR spectrum of compound **4** in CD_3OD

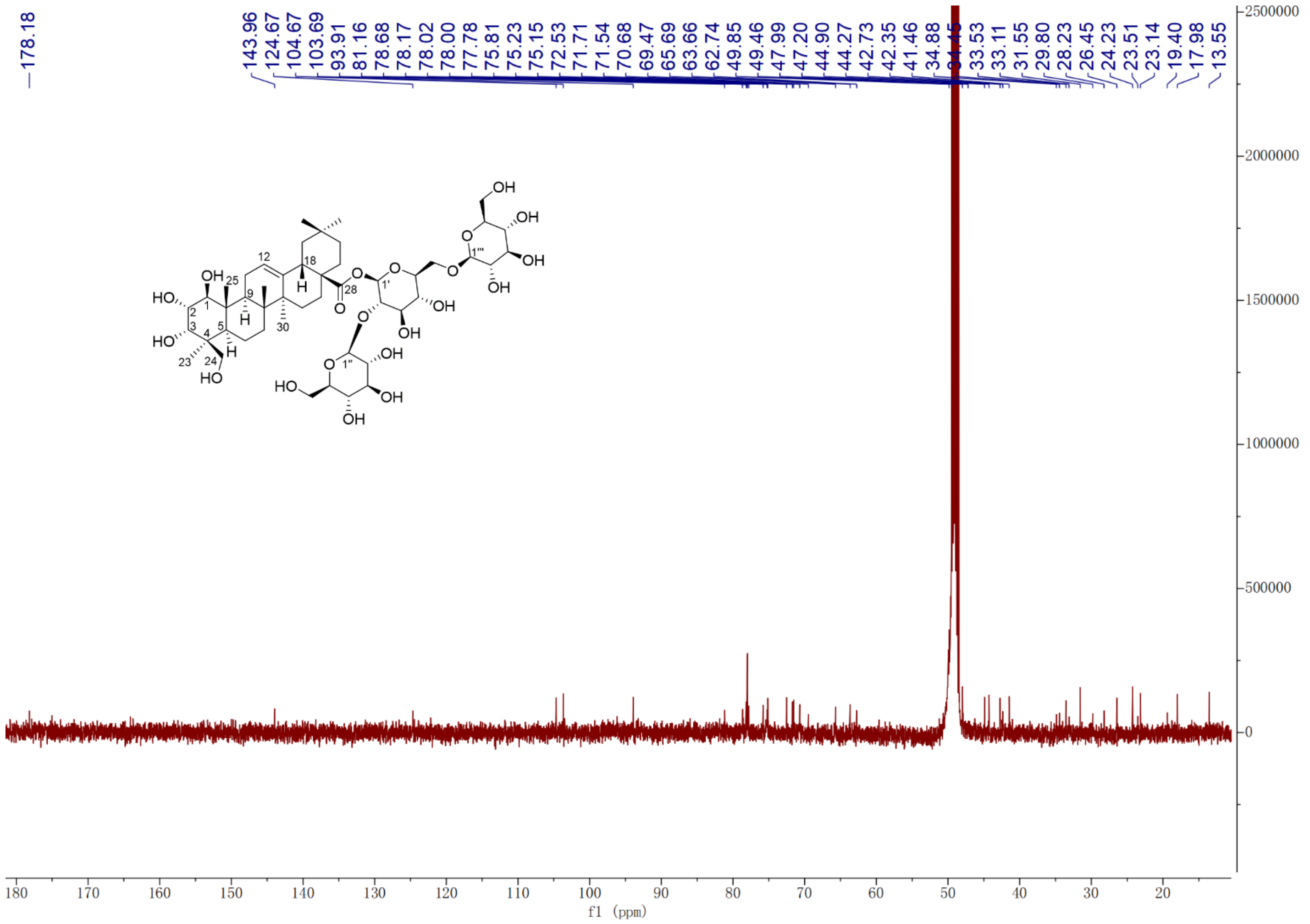

Figure S29. The ^{13}C-NMR spectrum of compound **4** in CD_3OD

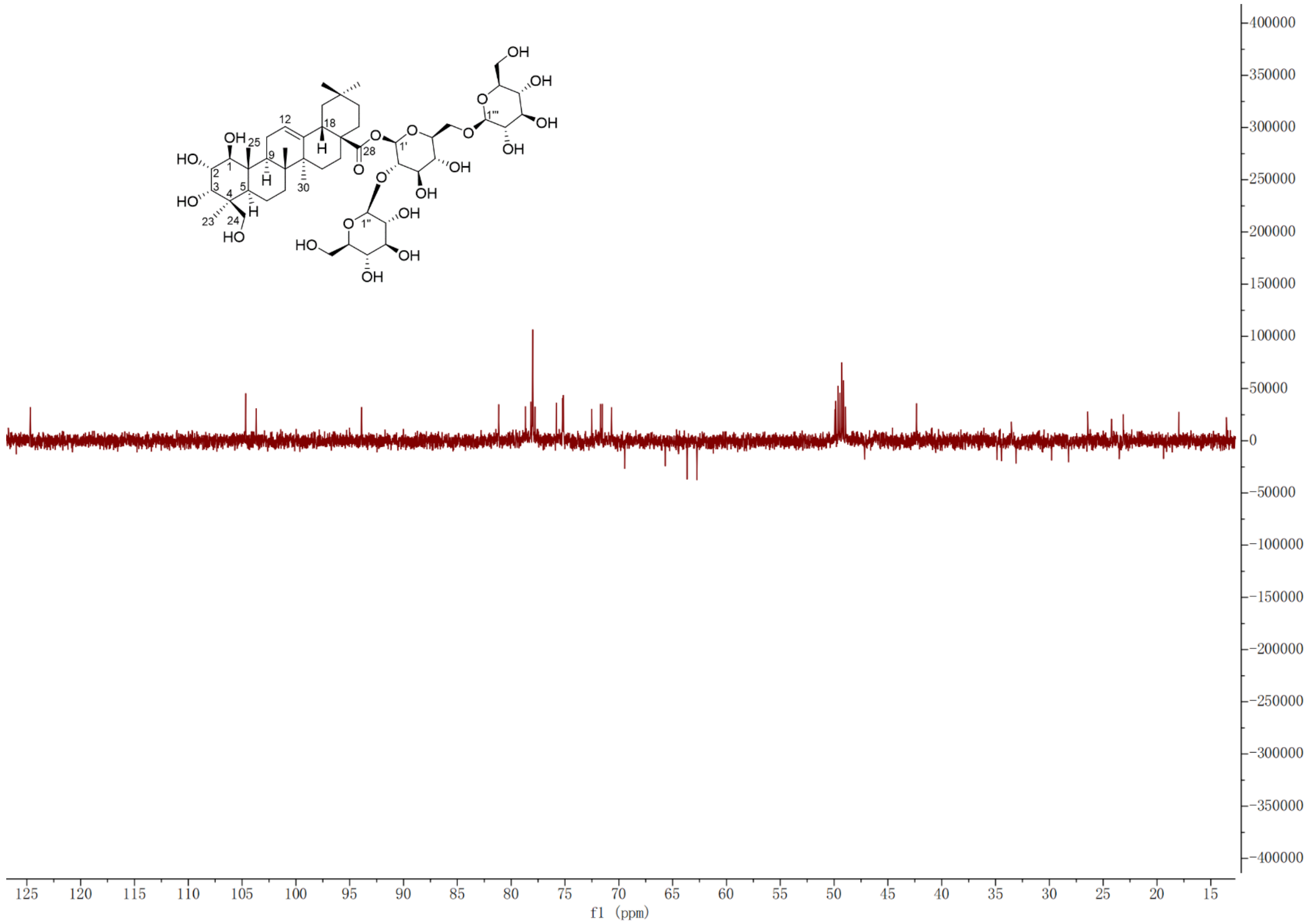

Figure S30. The DEPT-135 NMR spectrum of compound **4** in CD_3OD

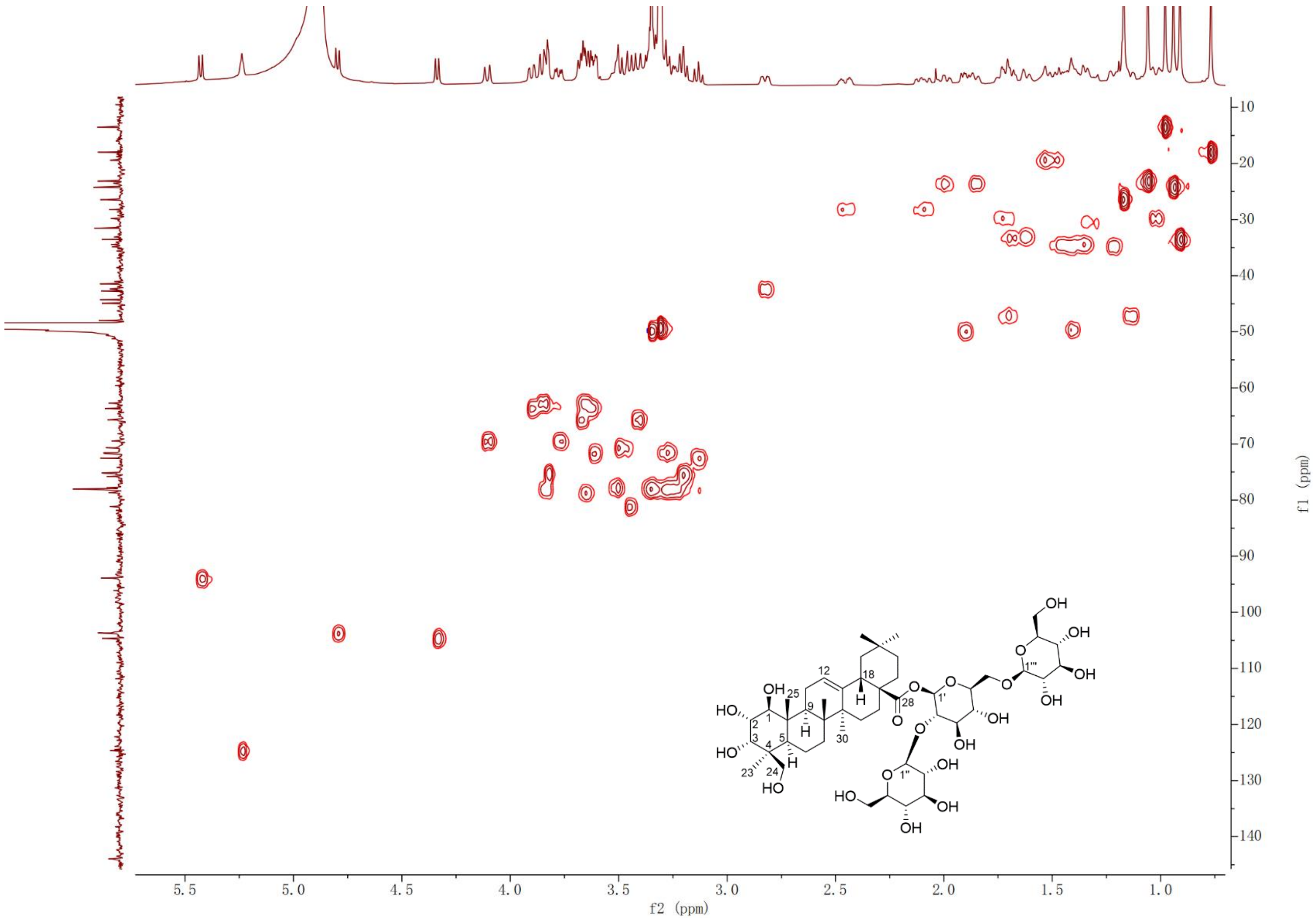

Figure S31. The HSQC spectrum of compound **4** in CD_3OD

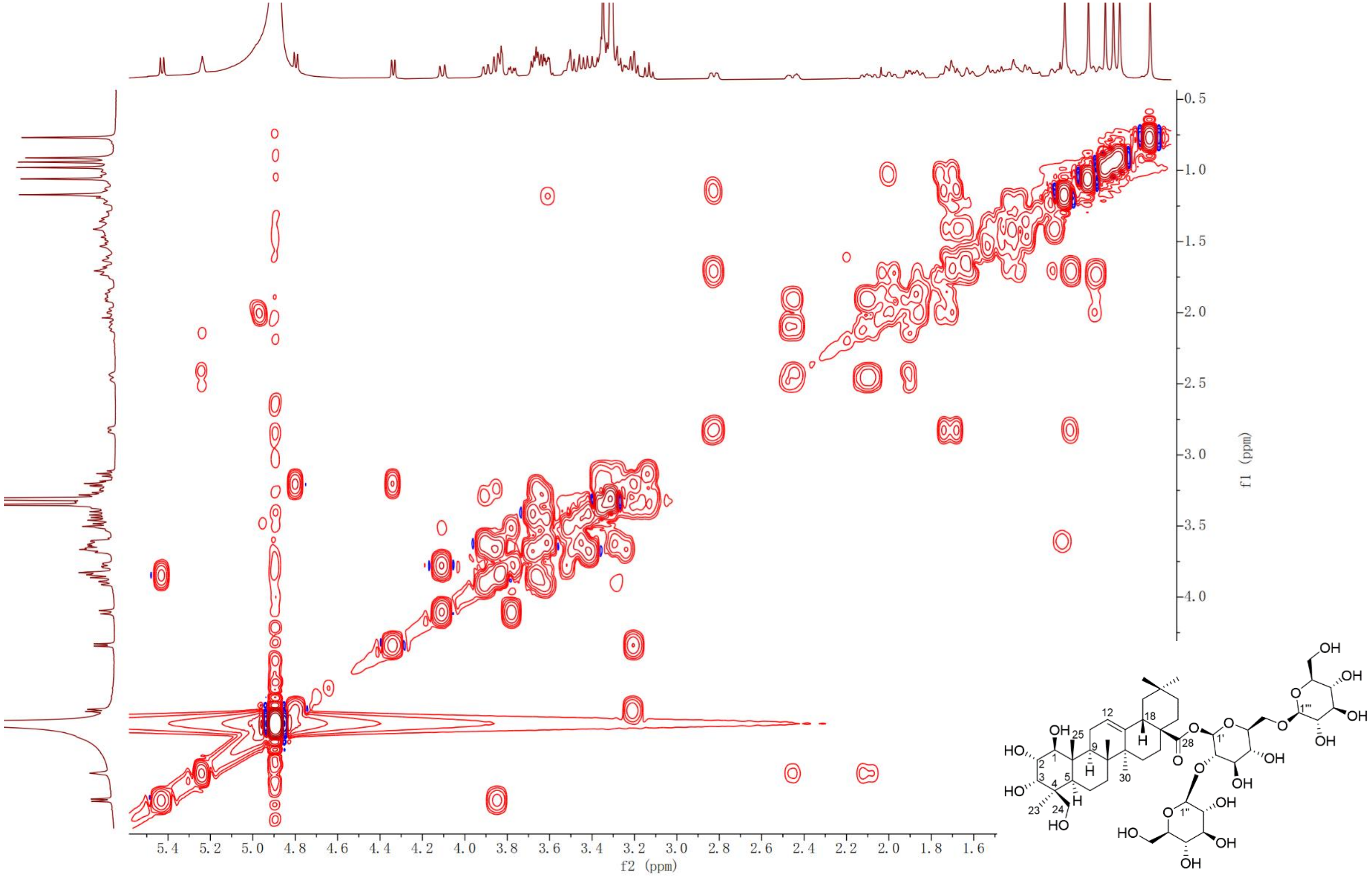

Figure S32. The ^{1}H-^{1}H COSY spectrum of compound **4** in CD_3OD

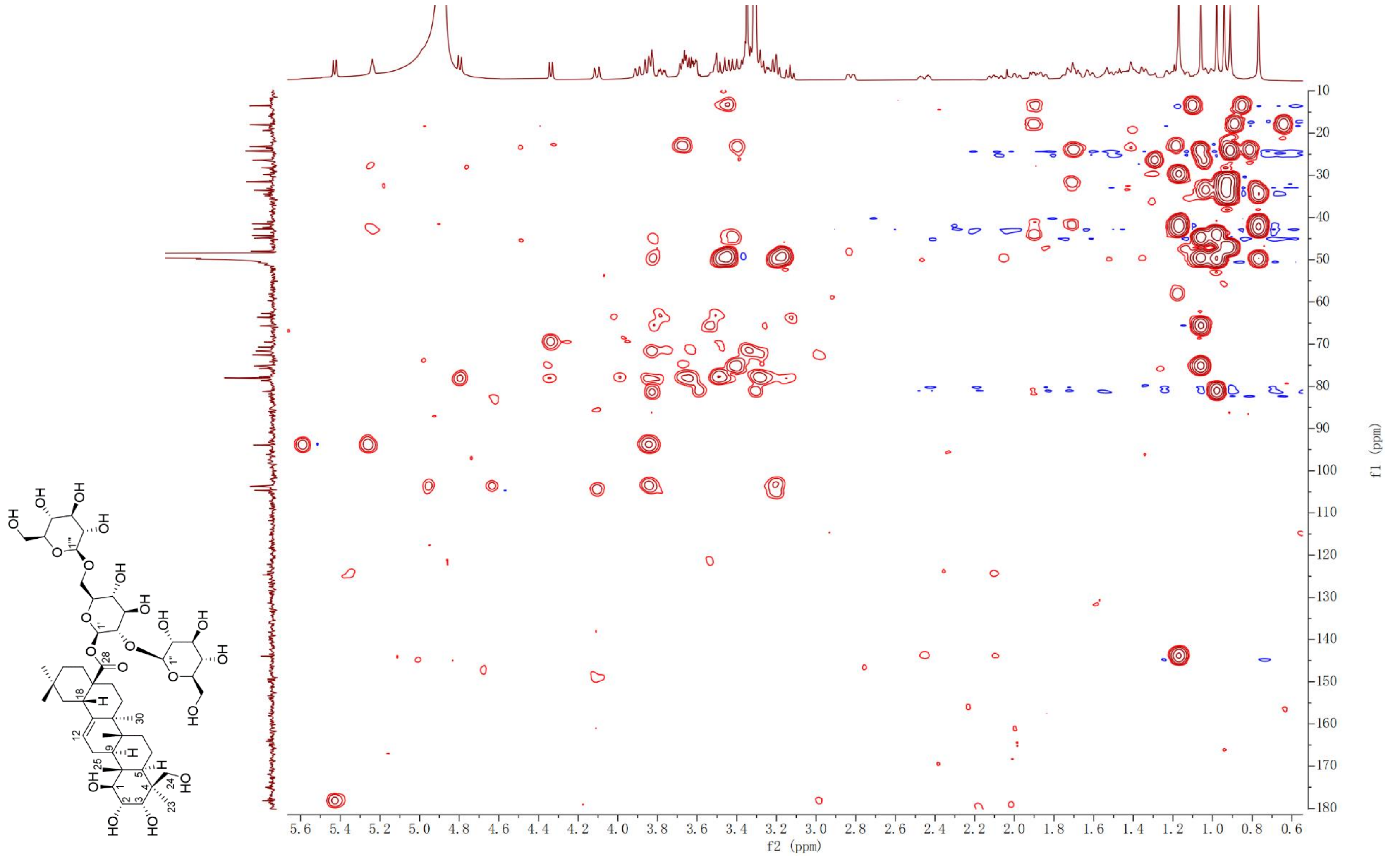

Figure S33. The HMBC spectrum of compound **4** in CD_3OD

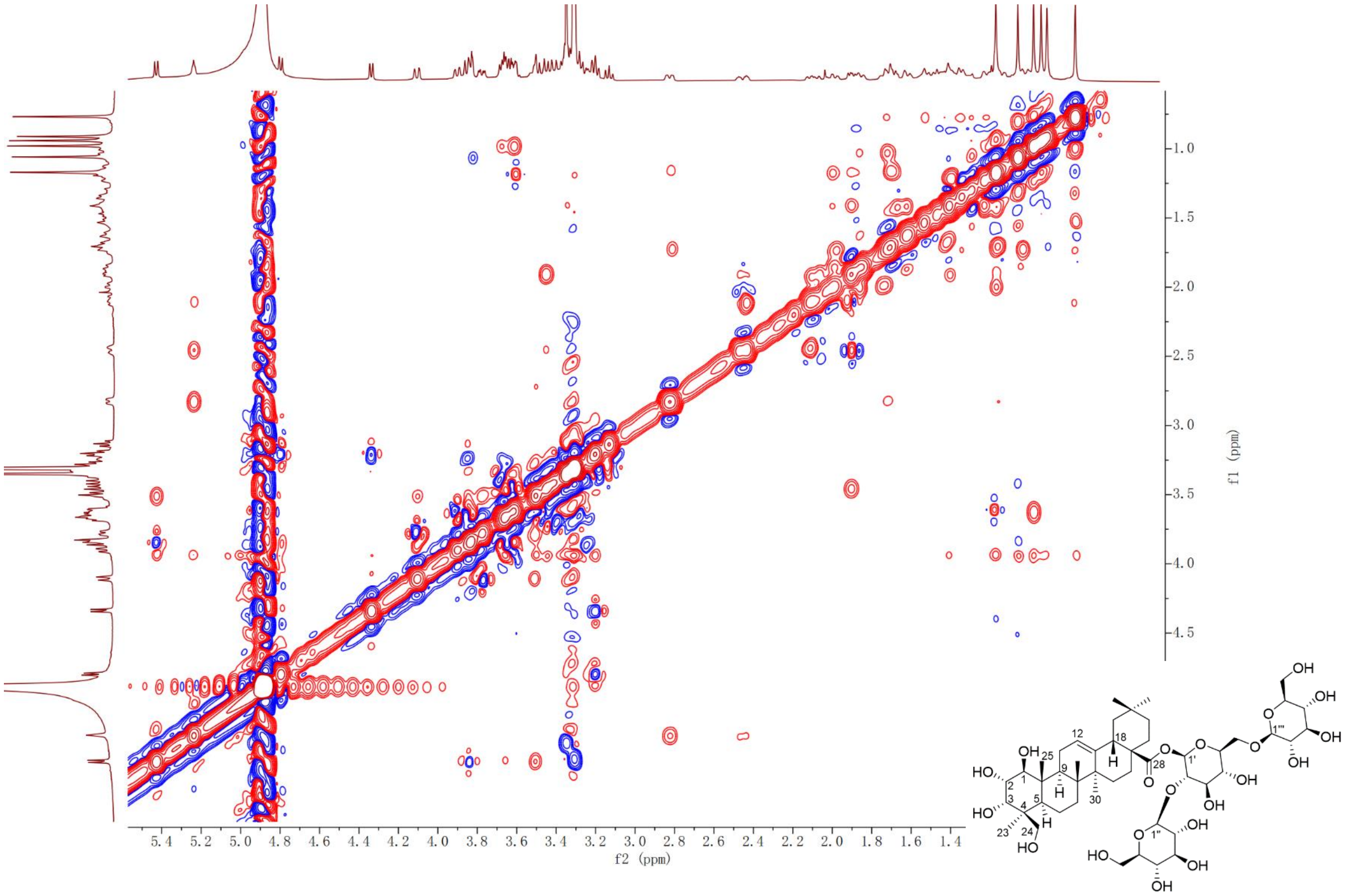

Figure S34. The NOESY spectrum of compound **4** in CD_3OD

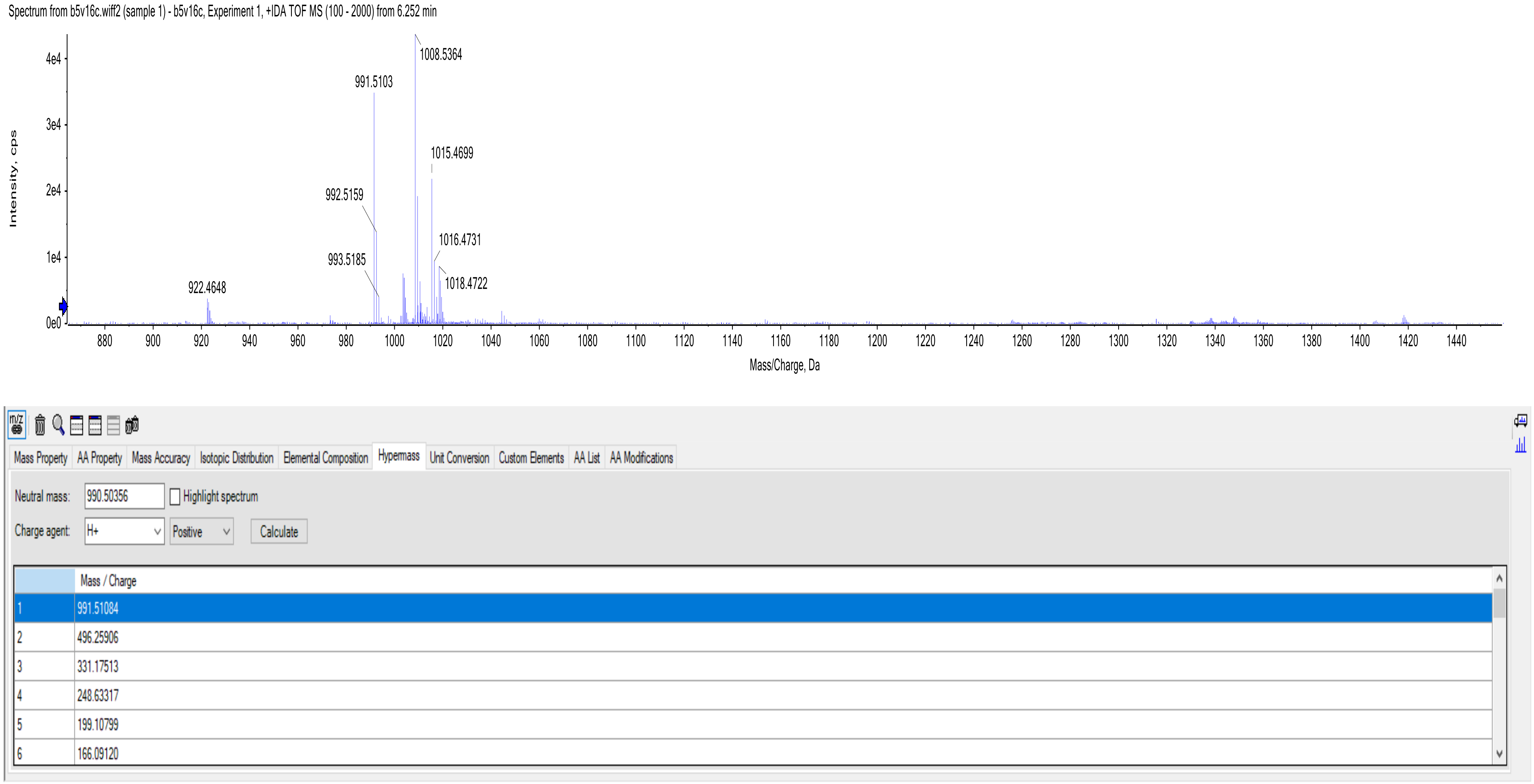

Figure S35. The (+)-HRESIMS spectroscopic data of compound **4**

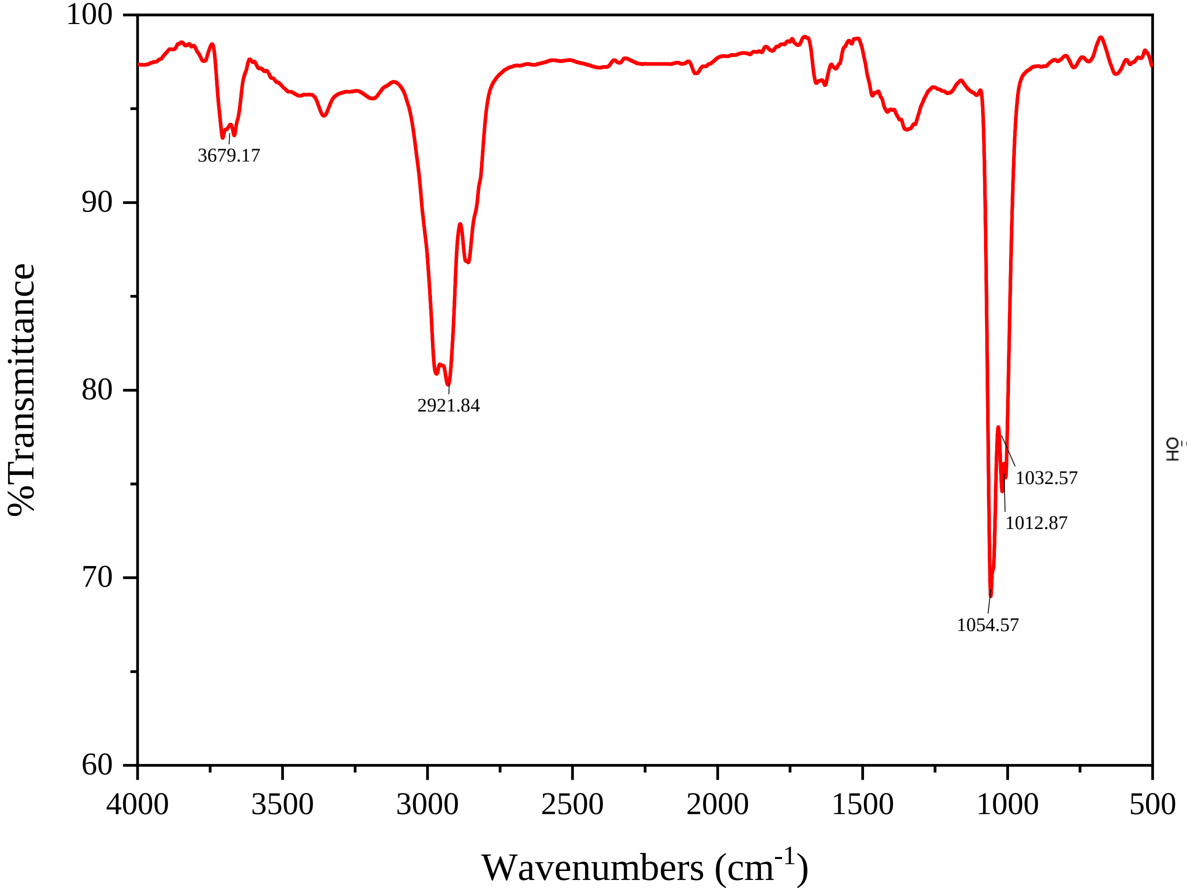

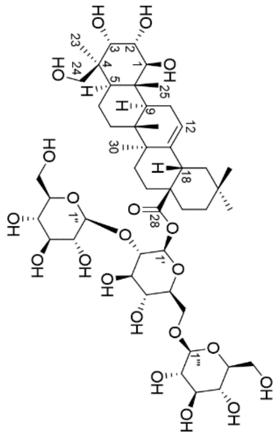

Figure S36. The IR spectrum of compound **4**

S37. The ECD calculation data

1 Conformers and energy analysis

Table S1. Energy (298.15 K) analysis for 6*R*-**1**

Conf.	G (Hartree)	ΔG (Kcal/mol)	Boltzmann Distribution
C1	-1956.116829	0.63566763	0.136933322
C2	-1956.117842	0	0.400584834
C3	-1956.117800	0.02635542	0.383147493
C4	-1956.114775	1.92457317	0.015532907
C5	-1956.114868	1.86621474	0.01714161
C6	-1956.115813	1.27321779	0.046659833
C7	-1956.114063	2.37136029	0.007251497

Table S2. Energy (298.15 K) analysis for 6*S*-**1**

Conf.	G (Hartree)	ΔG (Kcal/mol)	Boltzmann Distribution
C1	-1956.117447	0	0.803831417
C2	-1956.115028	1.51794669	0.061934779
C3	-1956.113703	2.34939744	0.015211288
C4	-1956.114084	2.11031613	0.022777351
C5	-1956.115444	1.25690253	0.096245165

The B3LYP/6-31G(d) harmonic vibrational frequencies for conformers of 6*R*-1

C1:

5.1179,6.3876,9.5236,16.3705,17.4111,31.7725,35.3705,38.8647,53.383,63.6341,75.155,81.2577,96.1117,107.9122,111.5028,117.6847,119.5505,123.2731,131.5051,139.6853,150.0673,157.4676,165.1609,181.2949,188.2932,191.4454,206.76,215.3326,221.9175,226.2391,227.3091,238.9861,247.7776,250.2516,254.8395,255.8869,289.7904,298.7329,304.316,307.0987,312.6854,323.0291,326.6613,336.6844,346.3083,348.477,376.7031,385.7457,390.0673,397.7312,400.7945,408.7786,415.3501,416.8271,420.6862,425.0257,426.3222,428.7665,436.8935,443.6347,456.4362,464.9449,468.6842,501.0793,502.6649,513.1675,514.4618,536.9647,552.4933,559.9805,568.9871,579.3343,590.8826,605.7449,607.2471,621.3268,632.8143,638.0659,643.7828,651.4411,669.2183,792.8962,829.9012,854.5061,856.2281,893.0527,896.5181,906.4897,917.951,927.3583,933.3585,958.846,959.5216,967.4996,991.938,994.2357,1001.3706,1009.9391,1013.2711,1022.8303,1034.1863,1037.5917,1044.0075,1045.8806,1057.6451,1062.1839,1063.0223,1066.1508,1069.8118,1074.2975,1078.4962,1079.1291,1094.9622,1095.2805,1

101.8163,1104.6342,1114.6036,1120.2685,1123.1456,1126.6422,1130.2017,1132.8643,1136.9175,1146.6509,1150.1228,1159.4456,1159.9885,1166.0443,1187.1033,1189.4623,1209.8245,1213.1633,1218.6348,1221.1465,1242.9942,1252.3998,1255.7868,1257.9335,1266.7312,1270.8394,1273.5027,1280.2187,1290.0222,1290.2205,1301.9851,1315.4139,1325.8083,1328.675,1333.0332,1334.9152,1351.0151,1352.6936,1356.5483,1362.2824,1364.7752,1370.4309,1372.5855,1380.7288,1382.5124,1384.8801,1389.4219,1398.2479,1412.6795,1420.2027,1421.2747,1429.6476,1430.7988,1436.5459,1440.9422,1441.6457,1446.6028,1449.8996,1450.9597,1451.2071,1454.6473,1459.3257,1464.4392,1467.3239,1475.5353,1490.3891,1505.4543,1513.5987,1514.9462,1516.3476,1518.792,1519.834,1529.713,1533.9772,1534.2044,1541.4794,1541.6465,1689.3026,1697.319,1711.8154,1772.0797,2954.277,2976.9954,2989.8205,2990.59,2997.6322,2997.9034,2998.1249,3014.7408,3019.3559,3021.6681,3028.4143,3029.4599,3030.383,3033.7705,3035.9899,3042.4076,3047.6863,3049.3136,3081.8802,3088.4552,3097.4909,3100.7125,3104.2588,3111.021,3121.0635,3122.7151,3129.1971,3131.0506,3136.4385,3138.185,3140.2513,3152.3508,3158.2493,3174.6528,3186.5594,3721.3906,3723.2311,3730.7347,3733.2934,3735.3926,3736.6421,3737.3146

C2:

4.7568,5.9448,7.8348,10.5552,22.0731,27.0167,34.8715,38.6462,52.4651,58.2171,69.4653,79.9031,95.818,101.9986,113.2836,116.2748,119.6597,123.8156,124.912,131.7733,146.3165,156.4306,158.7266,177.2362,183.8905,195.3461,196.7827,214.7712,218.2645,224.8299,227.9376,231.8252,236.5712,246.4453,252.6461,260.7028,279.7029,288.5009,302.5016,306.4328,310.6415,314.9567,321.213,333.9189,345.6421,348.8364,365.6951,382.5041,393.9129,396.7058,401.446,408.6876,414.3768,418.3198,423.0985,424.274,429.0682,434.4642,436.8627,443.765,450.9242,465.069,471.6945,498.6181,504.0804,513.7314,514.4534,528.475,553.2701,557.5721,567.5586,579.8757,591.1004,605.2392,616.2547,618.2668,637.1993,639.3954,646.6578,660.1746,677.1818,795.7868,836.2816,851.2586,854.9985,891.2969,897.5769,904.0829,917.3523,924.381,932.6919,958.6055,967.021,989.4412,993.0385,997.1122,1008.1356,1010.1103,1015.8571,1023.4672,1036.7139,1037.5733,1044.0779,1047.3465,1058.6549,1063.0028,1066.0542,1068.1127,1070.2656,1076.0105,1077.5069,1080.2699,1096.8505,1098.9378,1102.2347,1105.3149,1114.3189,1121.82,1127.4793,1128.8144,1131.8124,1133.906,1142.4557,1149.9508,1152.7125,1159.2896,1161.9834,1166.5495,1195.6194,1198.9588,1209.669,1213.8568,1220.7636,1221.9642,1244.1826,1245.7253,1253.8262,1258.3493,1266.814,1270.1378,1273.5799,1278.2865,1282.7406,1287.8566,1290.2422,1314.1708,1316.92,1328.1581,1333.2727,1334.8,1351.3243,1352.8503,1356.9231,1359.2564,1364.2456,1366.1007,1372.4859,1379.7524,1382.5739,1383.0001,1387.5647,1398.0953,1410.882,1421.0697,1421.6752,1429.4921,1429.5649,1436.3568,1441.3276,1445.2891,1449.4468,1450.6801,1452.0358,1454.3167,1459.4112,1460.5551,1464.6612,1469.1499,1475.2872,1489.8528,1505.425,1513.2943,1514.9988,1516.1745,1519.3491,1522.9504,1530.2944,1533.6567,1535.1957,1537.077,1541.5688,1693.2393,1700.8804,1721.4947,1771.2997,2942.5692,2950.9733,2989.3214,2989.4579,2996.9024,2996.9607,2997.657,3013.4088,3018.6,3021.8951,3022.255,3028.4602,3028.9186,3029.6931,3032.7544,3035.9752,3042.4649,3048.7195,3049.3109,3062.4905,3088.5058,3096.9814,3097.1357,3104.303,3110.9949,3115.2328,3122.7073,3128.9357,3135.8245,3138.0042,3141.5252,3153.5004,3162.3744,3172.9871,3185.9552,3721.374,3722.4248,3723.0414,3729.3089,3733.828,3736.3815,3737.5487

C3:

3.989,7.2417,17.8423,21.8655,25.3254,30.0301,39.0023,41.018,58.4105,70.533,72.0517,78.0403,97.2951,106.5332,114.5453,116.7365,120.6018,124.7462,128.1029,137.0844,152.2875,156.4238,165.1371,182.6108,195.4771,201.9003,204.8517,212.6296,222.6565,224.6059,227.0611,231.8605,240.9432,250.4242,259.7265,266.8051,274.2736,289.6887,300.4477,303.892,307.0994,320.3298,322.7645,333.7731,347.6116,356.3996,366.0873,381.0028,388.3275,388.7175,402.1689,402.6879,410.31,414.9338,422.1895,424.2278,428.7907,435.263,438.283,447.3463,457.767,466.2401,471.5902,490.5399,503.5165,505.316,513.8198,524.577,552.5308,555.6253,570.9634,580.5213,596.1496,608.8947,617.3664,623.9336,633.1667,639.1398,641.2312,662.2674,666.7136,803.5852,820.0967,854.811,862.0598,884.9043,895.8613,900.7507,910.5512,916.8588,932.7242,958.6541,966.9499,984.9169,992.6785,994.4246,1008.1873,1010.4419,1017.9121,1021.7002,1036.617,1037.7041,1043.7023,1045.5341,1059.3324,1061.7479,1062.532,1064.6065,1070.0598,1073.9752,1076.077,1081.0318,1089.8698,1098.9828,1101.4668,1103.4034,1113.3808,1117.2166,1120.5041,1127.665,1129.4951,1133.2362,1145.0532,1149.9308,1153.2985,1159.4203,1162.157,1165.6658,1186.9569,1203.1196,1209.8926,1214.2867,1220.7693,1222.3174,1243.5793,1249.0792,1253.9281,1256.6583,1269.4882,1270.0523,1272.9505,1278.7852,1283.4899,1290.158,1307.4151,1314.9723,1327.178,1328.1105,1334.3877,1334.6357,1351.3549,1352.8171,1357.8199,1362.2131,1364.9988,1365.2807,1372.3809,1379.7554,1382.4943,1383.3891,1387.6526,1397.6159,1411.3603,1422.0444,1425.6813,1429.6444,1431.7104,1436.0378,1439.9815,1444.2268,1449.0977,1450.4433,1452.0427,1452.2853,1456.8997,1459.9954,1463.8818,1467.1127,1470.4786,1490.0025,1505.529,1515.1161,1516.2,1519.3044,1522.441,1527.2972,1528.7717,1530.5227,1533.6792,1541.5918,1541.9108,1693.3103,1700.9615,1722.7144,1771.0435,2936.1095,2961.9239,2988.3152,2989.6687,2992.8533,2996.5328,2998.7249,3006.8254,3007.6122,3020.1702,3020.765,3029.5371,3032.9909,3035.6722,3039.6752,3042.4666,3042.7953,3049.2132,3071.9666,3088.351,3089.254,3097.2477,3104.0722,3110.8058,3112.3043,3123.7761,3128.5342,3135.4816,3138.0326,3139.2513,3139.827,3157.2264,3158.7625,3173.1581,3185.2379,3722.0708,3724.1671,3726.834,3729.124,3735.0323,3736.776,3740.928

C4:

5.6781,6.0782,11.7815,14.9116,16.9077,30.1645,38.5158,43.8917,53.9328,64.0701,74.2648,78.1651,99.9669,108.4127,113.1341,118.0807,120.11,122.4099,128.3386,142.4122,156.0017,163.1805,169.1765,176.6952,187.6586,194.0967,211.8248,216.1559,221.7906,227.3037,228.659,235.8557,248.713,250.5724,254.5398,269.3677,288.2357,298.5139,303.1697,306.9975,310.9043,320.3344,333.8927,339.2238,347.6655,348.9399,383.896,391.5073,396.2557,400.0534,405.4874,410.6685,415.387,417.5306,418.5881,423.2591,425.7089,428.1285,437.3364,447.3208,458.7103,465.805,470.5517,488.327,501.4855,513.0821,517.2733,540.6978,552.7665,553.9097,561.2759,579.3407,590.9388,606.2349,608.6885,616.4409,632.8358,640.6672,646.9442,669.2458,713.8002,800.5495,823.2077,850.1671,862.0361,866.3264,896.8085,900.8984,921.566,927.5313,932.9893,957.3174,959.5033,965.2121,992.1064,994.4347,1003.3666,1010.0181,1022.6091,1024.9138,1031.82,1034.3305,1043.5815,1045.5495,1055.264,1061.9359,1062.4069,1065.7306,1072.8368,1074.4783,1077.1714,1078.3727,1080.5,1095.6871,1101.2516,1104.5111,1112.591,1118.1032,1122.196,1126.0732,1130.1173,1132.2739,1137.0561,1147.0796,1149.498,1160.2875,1161.796,1166.2852,1187.3792,1190.0314,1203.3197,1213.3241,1218.7352,1224.582,1243.2729,1251.8537,1256.1793,1258.4048,1267.4038,1273.8764,1275.129,1286.3564,1290.5112,1292.8048,1304.0668,1315.6703,1325.9762,1333.555,1334.4145

,1335.0295,1341.2005,1356.9154,1358.102,1362.1701,1364.3877,1369.531,1371.5948,1382.
4087,1384.4874,1388.4817,1395.9242,1398.2628,1412.663,1419.7153,1421.4354,1428.6416,
1431.1188,1436.1329,1441.0904,1441.6552,1446.6611,1449.1966,1449.9878,1451.4702,145
4.3963,1459.5172,1464.5244,1467.3342,1475.6634,1485.4467,1505.9775,1513.5204,1515.27
82,1518.4024,1519.0616,1521.7095,1529.8304,1533.9811,1534.2591,1541.1457,1544.4868,1
691.4315,1699.0725,1712.503,1773.8621,2955.3724,2978.3518,2988.9756,2990.8325,2996.4
747,2997.5647,2997.6542,2997.95,3014.1576,3019.1268,3028.7101,3029.8272,3033.5836,30
34.5036,3037.0947,3040.5332,3047.0903,3048.8724,3081.7393,3090.0045,3098.3266,3101.2
51,3102.8373,3110.3661,3120.6792,3121.3303,3130.1327,3130.6457,3135.3995,3137.8885,3
152.5951,3158.8491,3162.3673,3180.221,3184.8872,3720.4214,3722.8131,3730.2864,3732.3
382,3734.9374,3736.7712,3737.0787
C5:
5.9221,6.6204,10.6272,16.4002,16.8192,27.5633,35.4216,37.3915,53.2667,63.0132,74.5895,
79.001,96.8488,108.019,112.5541,117.209,118.3793,121.7073,129.4242,144.4116,155.3876,
161.7694,167.0503,177.0283,187.5236,201.5931,206.9991,215.227,216.0948,222.3174,227.2
707,243.0582,250.0698,252.536,259.8345,266.4663,292.5138,299.046,304.4785,307.4279,31
1.8887,329.7684,332.7189,339.3245,348.6522,362.3645,384.0562,387.8511,391.1809,397.49
8,400.7717,407.623,413.0061,417.2281,418.1388,423.2434,425.5766,427.9337,436.8291,445
.5567,454.6522,462.1708,468.5283,474.4049,501.2772,506.816,514.3225,540.4887,549.4165
,568.0177,578.4553,584.8298,591.4553,606.3199,613.0311,626.5129,633.6875,640.9005,646
.0842,653.7583,668.7848,783.2907,819.5049,852.0473,857.677,886.4471,896.5103,907.7226
,919.1503,927.5674,935.479,954.2431,958.1442,960.1695,993.5107,998.1984,1001.5083,100
9.9415,1020.7054,1023.0753,1034.1845,1038.2549,1045.4549,1049.758,1054.8528,1062.155
9,1063.0437,1065.9589,1073.3256,1074.2342,1078.3739,1079.3573,1095.1735,1098.0031,11
02.4058,1105.0661,1115.3378,1121.1145,1125.3462,1128.1449,1130.8866,1135.8388,1136.7
517,1146.9585,1148.5702,1157.7748,1160.1091,1166.1695,1187.3136,1189.1893,1199.2635,
1213.2109,1218.508,1233.0137,1242.908,1252.3393,1255.7206,1257.8374,1262.9123,1266.7
697,1273.587,1281.4767,1290.5989,1297.3524,1302.1291,1315.4583,1319.5898,1326.0435,1
332.9837,1334.7957,1349.6577,1352.8729,1356.5956,1362.2934,1363.8825,1364.794,1370.5
696,1373.8638,1382.554,1384.7854,1388.9753,1398.1723,1412.7451,1420.4286,1421.3693,1
429.4401,1430.8142,1440.9097,1441.1213,1441.7585,1446.5944,1449.6986,1450.3918,1451.
1738,1454.6443,1459.2992,1464.4812,1467.3162,1475.6363,1488.005,1505.635,1507.3369,1
513.2665,1517.5894,1518.5152,1524.5336,1529.7132,1534.0129,1535.6978,1541.1864,1543.
3578,1689.2454,1695.7078,1716.9405,1776.145,2955.4147,2977.4224,2978.0183,2989.2773,
2990.6349,2996.8505,2997.8624,2998.0592,3014.3894,3014.8035,3019.4073,3028.9769,303
0.5096,3033.974,3042.2865,3042.9612,3047.5729,3053.9318,3082.1489,3091.2915,3099.292
3,3099.7724,3106.4022,3116.404,3121.2447,3124.2017,3125.9719,3131.1404,3136.3925,313
8.1539,3139.6218,3152.8684,3159.1423,3175.4921,3185.935,3721.2518,3722.8929,3730.648
8,3733.0597,3735.2795,3736.7686,3737.1946
C6:
3.6435,5.9797,8.5556,10.4855,20.2981,28.9298,33.3306,44.1914,55.4734,59.537,68.6927,78.
9913,96.1982,102.7308,113.7358,117.3192,119.3163,122.3388,124.5536,146.8213,148.9604,
158.8607,170.3649,178.144,184.367,188.4105,197.9325,215.0213,218.5988,226.3279,227.24
07,233.6356,236.1274,248.6353,253.0008,271.4648,278.6573,287.525,302.3349,305.091,307

.9149,316.3478,318.3677,342.9966,348.8856,354.4599,363.8683,392.749,396.7851,400.8138,407.5689,412.8732,414.6275,416.2262,421.3601,423.4728,424.7947,434.5999,436.5328,444.2498,452.8373,466.8518,474.8134,488.32,500.4595,514.7611,516.7305,529.3929,551.1694,554.0223,565.9155,579.3482,591.2915,605.3733,612.8903,618.895,638.6948,646.5101,650.1112,676.1782,720.6986,798.8242,829.3519,848.832,859.833,868.4337,896.9028,897.7675,919.6698,923.3844,933.4423,957.6089,964.5477,990.0316,994.7075,998.2648,1009.9819,1013.068,1023.2647,1024.9853,1032.2778,1036.8284,1043.592,1047.0053,1056.2557,1062.9661,1066.4857,1068.0578,1073.9685,1075.9808,1077.5148,1079.2108,1080.1318,1096.9447,1100.908,1104.9878,1112.2384,1121.6345,1126.9998,1127.8341,1131.8512,1133.0867,1142.5711,1150.0666,1152.9768,1160.8874,1162.7671,1166.7346,1196.5846,1200.2584,1203.7591,1213.9702,1222.39,1224.7562,1244.5225,1246.4425,1254.628,1258.8778,1267.0172,1273.5614,1275.4542,1277.6564,1286.7696,1288.1803,1296.9015,1313.6239,1317.2412,1333.2765,1334.8764,1335.0437,1341.369,1356.9099,1358.3589,1359.0546,1364.6471,1366.2063,1370.5128,1381.2634,1382.6196,1386.8475,1395.996,1398.0412,1410.7399,1420.6623,1421.5212,1428.349,1429.5211,1435.9369,1441.2484,1445.6037,1448.9269,1449.3919,1452.0553,1454.6056,1459.2304,1460.1362,1464.5598,1469.3154,1475.0214,1486.2268,1505.964,1513.4663,1515.332,1519.0002,1521.5573,1523.3446,1530.3797,1534.1515,1535.0338,1536.06,1544.5571,1698.5469,1699.5349,1722.3522,1773.6247,2941.396,2951.6093,2988.9893,2989.6163,2996.0216,2996.7061,2996.9557,2997.8335,3013.2218,3018.5496,3019.6843,3029.0851,3030.5842,3033.7705,3034.7281,3037.0303,3039.9163,3047.4245,3048.3864,3059.323,3090.2354,3095.2752,3100.7055,3102.0182,3109.7538,3114.9429,3121.1414,3130.27,3135.6582,3137.9073,3152.0333,3159.351,3166.1516,3178.0795,3185.1947,3719.9027,3721.1874,3722.7187,3728.5368,3734.0873,3735.3562,3737.4944

C7:

3.6434,5.6311,8.9419,10.6405,18.3578,27.1258,34.0871,39.8659,47.1691,52.3673,60.3127,76.81,96.3563,104.0922,114.975,116.8335,118.844,123.9437,132.2881,137.361,143.1821,147.2232,154.7332,169.4442,186.8971,193.6467,196.6003,210.2544,217.4038,219.0431,226.1673,229.2615,234.1558,246.0422,252.2269,254.7963,274.8217,286.4851,293.1406,302.7153,306.5438,315.6668,321.2464,339.733,347.3991,349.2839,361.8426,384.6166,392.6916,397.169,403.728,409.2701,414.1186,417.7205,423.1381,424.1255,430.3452,435.9389,437.8702,444.7402,453.8475,459.2469,467.5875,485.842,501.5799,513.4499,517.5267,526.4086,550.9921,555.6987,566.681,579.0292,591.0262,603.2296,612.2843,623.6698,637.7581,644.6516,646.9054,649.8597,675.9056,807.5401,831.3817,854.8065,868.2973,892.9064,897.6024,902.8624,920.192,926.8169,933.6985,958.4779,967.5981,988.3475,991.598,996.6997,1004.9064,1009.934,1019.0297,1023.0377,1025.4532,1036.8422,1038.3778,1047.0491,1049.8137,1062.9303,1066.6546,1068.0691,1070.5076,1076.013,1077.9559,1080.0391,1096.9779,1100.8758,1104.8708,1107.5408,1113.4969,1121.9518,1127.6125,1129.6844,1131.7767,1134.0848,1144.0464,1149.8489,1153.5417,1159.4004,1162.566,1166.7826,1195.8207,1199.4465,1210.1585,1213.8715,1221.3828,1222.0149,1227.4738,1244.6321,1253.7913,1258.4833,1264.54,1267.065,1273.7522,1274.3234,1281.6169,1287.9563,1291.0602,1313.2099,1317.2891,1327.1889,1333.3342,1334.9359,1342.873,1349.4949,1356.662,1357.9024,1361.8004,1365.736,1367.895,1375.6029,1382.8887,1384.1498,1390.8453,1398.1423,1409.9361,1417.0233,1421.4522,1429.4259,1429.6802,1436.5757,1441.2022,1444.2807,1445.7245,1450.3128,1452.1535,1454.5035,1459.4587,1460.5915,1464.6394,1469.2533,1475.1519,1490.4454,1505.2113,1511.9428,1514.7

952,1516.5458,1519.5751,1519.8309,1530.3144,1533.6996,1535.2174,1538.2876,1541.7975,1694.2935,1703.309,1735.8548,1771.6705,2941.5485,2951.6844,2989.2235,2989.7038,2996.712,2997.1063,2997.9264,3013.2473,3018.6629,3021.1554,3024.5845,3029.0342,3029.6454,3030.3453,3033.6007,3036.0054,3040.9641,3041.9348,3049.1288,3063.8206,3087.4075,3088.7,3097.2628,3103.7067,3110.67,3114.9849,3122.4188,3127.1996,3129.2636,3136.5372,3137.856,3150.2209,3160.7842,3177.5157,3186.6955,3720.3701,3721.1883,3722.9211,3728.5592,3734.1153,3735.853,3737.3425

The B3LYP/6-31G(d) harmonic vibrational frequencies for conformers of 6*S*-1

C1:

4.7328,7.8051,8.6131,10.7936,21.7208,27.5905,34.3683,39.9264,53.387,57.9146,68.002,79.123,95.6853,102.4628,113.6902,116.4439,120.7424,123.9915,126.7481,129.0806,146.6435,157.343,158.6181,178.0619,184.3753,195.6934,197.5769,215.0891,218.3542,224.6216,228.1073,232.4194,236.0821,246.6035,252.9165,261.7789,278.6363,288.4897,302.697,305.2223,306.6987,315.0566,322.257,332.8353,347.9275,349.0971,365.3485,381.2887,393.2407,396.5487,401.9667,408.2151,414.3282,417.6882,422.7115,424.4336,428.0585,434.7342,438.2821,445.0817,452.7707,465.2617,473.928,498.1605,502.8751,511.9338,515.0381,527.2023,552.0576,556.0087,569.1392,580.0485,591.2926,605.4312,616.2668,618.8498,637.0825,639.3375,646.8054,661.2677,677.3449,806.1889,817.1075,854.7997,863.2893,888.7133,897.6669,903.8898,916.448,924.1595,933.0253,958.7022,967.2404,989.6434,993.1912,997.159,1008.248,1010.1028,1015.9328,1023.3991,1036.7699,1037.5708,1044.0266,1047.3707,1058.6606,1063.0402,1065.9701,1068.3415,1070.187,1076.1173,1077.5311,1080.2685,1096.841,1098.6881,1102.2402,1105.3252,1114.3425,1121.8788,1127.3282,1128.7054,1131.8767,1133.8775,1142.6689,1149.8623,1152.9962,1159.1555,1161.9506,1166.5827,1195.982,1199.5015,1209.5724,1214.0215,1220.7799,1222.2689,1244.4045,1245.8755,1254.0142,1258.6689,1266.8753,1270.1885,1273.7254,1277.9045,1282.5245,1287.8255,1290.4033,1313.9397,1317.1875,1328.0332,1333.3733,1335.0762,1351.5841,1352.7468,1357.1419,1359.399,1364.2821,1366.3825,1372.5349,1379.3286,1382.6558,1383.1385,1387.4157,1398.225,1410.9832,1421.0569,1421.7542,1429.4558,1429.6654,1436.4779,1441.4308,1445.3067,1449.4015,1450.618,1452.1341,1454.48,1459.3895,1460.4151,1464.6314,1469.2293,1475.1108,1489.4353,1505.4677,1513.4193,1514.9541,1516.1884,1519.2977,1523.0668,1530.3257,1533.6525,1534.9811,1536.7456,1541.5404,1693.2923,1700.9696,1721.771,1771.2881,2942.3588,2951.1429,2989.4215,2989.6777,2996.9852,2997.0538,2997.8302,3013.4987,3018.7057,3021.4821,3022.0228,3028.9113,3029.2058,3029.9364,3032.9441,3036.083,3042.3314,3048.8181,3049.2659,3061.7101,3088.6604,3096.98,3097.0261,3104.1631,3110.9188,3115.1927,3122.5252,3129.0264,3136.0869,3137.9919,3141.5056,3153.3774,3162.3855,3172.6906,3186.0926,3721.0513,3721.6041,3722.8737,3729.0468,3733.5508,3735.9267,3737.3608

C2:

4.3509,7.5542,10.1091,10.8578,23.9502,27.244,35.764,41.7547,52.2778,57.6985,72.781,83.6024,95.7652,99.2258,113.4185,116.4655,121.3675,123.4635,125.5432,146.1482,148.4007,158.7167,168.9102,177.3993,183.2435,188.9123,198.2166,214.3643,218.3138,225.2108,229.6653,232.9189,234.6421,248.718,252.5174,272.846,278.9709,291.0351,302.1638,305.2678,308.5481,315.205,318.2669,342.2013,348.1665,349.7728,365.3972,393.6196,396.6535,399.7955,408.0078,412.3624,414.9746,416.6557,421.0322,423.3901,424.9338,434.2541,435.504,444.

0895,451.9004,466.9733,472.1487,491.7221,500.3883,513.7612,517.1,530.2496,552.5473,555.4094,565.0473,579.3454,590.8895,605.3523,612.8454,618.585,638.7116,646.4153,649.5138,675.7292,725.598,794.1884,828.2467,849.3279,854.5909,875.0477,897.4057,899.0426,917.1766,924.384,933.9796,958.0271,965.2325,989.7306,994.434,998.1629,1010.1112,1012.1674,1023.4553,1025.2834,1032.5176,1036.9338,1043.7852,1047.2086,1056.3843,1063.0124,1066.2333,1068.2875,1073.825,1076.0807,1077.9131,1079.162,1080.313,1096.9699,1101.1051,1105.1026,1112.5172,1121.6173,1127.1106,1127.7811,1131.9995,1133.7833,1142.2233,1149.5173,1152.879,1160.8858,1162.7215,1166.7836,1196.134,1199.0179,1203.833,1213.9778,1221.8607,1224.8686,1244.4335,1246.5265,1254.2923,1258.3459,1266.6694,1273.9666,1275.2273,1278.1888,1286.4128,1287.7607,1296.5104,1314.3101,1317.1142,1333.2734,1334.5982,1335.0153,1341.4026,1356.9009,1358.1893,1359.3005,1364.147,1366.0628,1370.2825,1381.4509,1382.7456,1386.9857,1395.978,1398.1541,1410.9222,1420.7763,1421.5619,1428.612,1429.7079,1436.0196,1441.4176,1445.3965,1449.2748,1449.3119,1452.1117,1454.4233,1459.5566,1460.4755,1464.8413,1469.3077,1475.3944,1485.6129,1506.0357,1513.4992,1515.4318,1518.9463,1521.5996,1523.1683,1530.3006,1534.2149,1535.4424,1536.8599,1544.4844,1698.8436,1699.2669,1722.1558,1773.9696,2940.9445,2952.3452,2989.39,2989.4369,2995.8224,2996.6938,2997.2764,2998.017,3013.4095,3018.7066,3021.7718,3028.679,3029.9925,3033.3833,3034.0789,3036.8542,3039.9819,3047.4548,3048.4385,3060.7878,3090.1203,3095.3029,3101.0261,3102.1636,3109.8285,3114.8821,3121.4648,3130.514,3135.429,3137.8988,3152.4832,3159.2725,3165.977,3177.7583,3185.1447,3721.3958,3721.6388,3722.2792,3728.8451,3733.4065,3735.9841,3737.368

C3:

3.7743,5.8779,9.1486,11.2813,18.3459,26.6844,33.9639,41.0282,46.6316,51.0035,59.5382,77.8389,96.1254,104.5944,114.7393,116.4825,118.403,123.9037,129.6984,139.57,143.252,147.0595,156.8462,170.3019,187.2896,192.8932,198.1468,211.772,217.9334,220.4375,225.9466,229.4063,233.6725,244.565,251.5302,254.232,275.308,290.1729,295.2166,302.6126,306.3995,310.0357,317.213,343.12,345.2422,348.7343,359.4903,387.9358,392.8521,398.0727,405.6663,408.4964,413.9677,415.7298,420.7169,423.2924,424.7361,434.9872,436.1845,444.0245,454.1452,463.7449,473.5217,488.8077,503.5978,511.5424,517.1477,524.1926,550.5308,554.7332,565.475,579.4748,591.082,603.3295,611.9627,626.8571,638.2075,644.7431,647.1986,649.7987,675.9378,800.951,847.5079,853.5728,856.1429,891.9123,897.6478,906.3396,920.1607,926.8675,932.9931,958.5548,967.1701,987.9725,991.5206,996.9185,1005.1838,1009.9612,1018.7308,1022.9985,1025.1089,1036.8395,1038.9074,1047.0893,1049.9243,1062.924,1066.7204,1068.1347,1070.9036,1076.0152,1077.8705,1080.0357,1096.9854,1100.8492,1104.8349,1107.1529,1113.4262,1121.9351,1127.6441,1129.6434,1131.8409,1134.1565,1144.1538,1149.9104,1153.6234,1159.4306,1162.6707,1166.8347,1196.016,1199.5273,1209.6997,1213.8489,1221.4764,1221.8721,1227.3835,1244.6225,1253.7614,1258.4735,1264.4944,1267.1636,1273.7714,1274.2112,1281.93,1287.9515,1290.0085,1313.0363,1317.2369,1328.2881,1333.3252,1334.9542,1343.5802,1349.8641,1356.6864,1357.9328,1361.9806,1365.8211,1368.4065,1376.0425,1382.8659,1384.0618,1390.6805,1398.1439,1409.9414,1416.9606,1421.438,1429.5511,1429.668,1436.2135,1441.2182,1444.1921,1445.7248,1450.5481,1452.1474,1454.5101,1459.435,1460.5385,1464.5899,1469.217,1475.1289,1490.0411,1505.4043,1512.1377,1514.7786,1515.87,1519.2043,1519.7941,1530.3178,1533.5575,1535.2169,1538.2355,1541.3557,1694.8383,1704.6264,1736.5292,1771.9355,2941.5845,2951.6134,2989.1409,2989.6808,299

6.7229,2997.0037,2997.8847,3013.2228,3018.6416,3021.6446,3024.3514,3028.9929,3029.38
98,3030.1866,3033.4181,3035.7278,3040.6114,3042.6703,3049.4179,3063.6242,3086.5688,3
087.7056,3097.6503,3104.5766,3111.1794,3114.9118,3122.5112,3127.3738,3129.2177,3136.
0787,3137.8459,3150.3112,3160.9,3177.141,3186.0819,3720.455,3721.1171,3722.8547,3728
.5449,3734.0787,3735.6682,3737.2706
C4:
4.2828,5.7747,9.0272,10.5209,21.5619,27.1351,36.1598,38.2001,48.7908,56.6812,69.6736,7
4.501,92.3824,100.711,111.2375,116.5468,118.7314,121.7039,125.3848,138.4929,146.8185,
153.8715,155.7746,178.5091,183.726,190.816,197.1379,211.3784,218.0633,220.6687,225.00
86,228.0841,231.9346,239.6818,252.0725,252.3272,275.5859,290.2909,301.7703,303.6756,3
06.7476,313.7795,316.9902,340.2266,345.0583,348.9221,364.385,388.7701,395.4126,396.62
83,402.5405,409.7396,414.2634,418.1742,423.2619,424.1403,431.1639,435.2749,439.2943,4
46.9303,451.1094,460.8013,467.6375,495.3556,502.4633,511.6608,518.0647,531.6375,553.0
686,557.6696,564.5419,578.9265,590.8959,602.8957,612.634,623.5126,637.8218,645.3804,6
46.6275,654.0965,676.3009,795.8527,833.1923,854.9846,858.5679,896.2391,897.3553,897.9
069,923.4537,931.8621,934.6191,958.625,967.6458,989.2374,992.0409,996.9954,1007.0168,
1010.0138,1015.0392,1022.9775,1026.0665,1037.0474,1038.3454,1047.2144,1049.9846,106
2.9543,1067.0946,1068.4883,1070.8338,1075.8687,1076.5818,1080.2101,1096.991,1100.874
,1104.8575,1107.4444,1113.655,1121.821,1127.4798,1129.9964,1131.9138,1135.0617,1144.2
44,1149.7417,1154.2905,1159.4224,1163.1898,1166.9972,1196.3342,1199.3807,1210.2049,1
213.8995,1221.3834,1222.2901,1227.3922,1244.5765,1253.7452,1258.4721,1265.2332,1267.
2034,1273.7391,1274.3686,1281.3797,1287.8447,1290.7581,1313.2727,1317.2845,1326.945
3,1333.3232,1335.1082,1342.1638,1348.3434,1356.6025,1357.8075,1361.5684,1365.6096,13
67.5931,1375.3043,1382.7213,1384.047,1392.0371,1398.1088,1409.4537,1415.7287,1421.64
94,1429.4382,1429.7121,1436.7636,1441.4553,1444.857,1445.8606,1450.3627,1452.0658,14
54.4686,1459.1286,1460.0743,1464.7028,1469.2115,1475.2392,1490.3612,1505.5174,1513.9
386,1515.0755,1516.228,1517.1517,1519.674,1530.3739,1533.7703,1535.0932,1537.6361,15
41.4314,1694.1498,1701.7043,1731.1099,1771.8487,2940.4618,2952.1079,2989.4256,2989.5
015,2996.7255,2997.2008,2998.1141,3013.2768,3018.9106,3020.8996,3021.6216,3029.1031,
3029.6142,3030.1449,3033.3524,3035.9265,3041.8404,3042.1587,3049.0229,3059.8271,308
8.2064,3088.7658,3097.4939,3103.6429,3110.648,3115.2201,3122.6602,3127.3844,3128.844
8,3136.2685,3137.9641,3144.8281,3161.8637,3173.9542,3186.4713,3721.2342,3721.343,372
2.5472,3728.8053,3733.2908,3735.9212,3737.6111
C5:
4.5115,7.6642,9.204,11.834,20.9937,25.4618,34.5102,38.3979,53.3669,58.4806,68.1971,79.0
314,96.3039,102.1875,114.2348,116.5533,117.2678,122.6989,124.3281,135.7695,146.7508,1
58.3327,162.6817,176.5885,182.0312,192.7574,202.4319,214.7409,217.5184,220.9844,229.7
027,232.0259,244.2829,252.3702,262.1184,266.2423,279.999,294.3262,302.5359,305.9111,3
08.2421,316.578,332.9475,339.7728,348.5816,357.8582,367.6985,388.1091,394.3156,397.87
87,402.1556,408.8977,414.4462,417.4217,419.9472,423.3821,424.6439,433.7267,435.4034,4
43.4077,452.7906,454.9277,469.5292,479.0955,500.9725,508.9417,515.7154,528.3023,547.6
93,566.7384,577.6805,588.9743,592.5222,605.9297,617.6005,625.2566,638.5004,645.578,64
8.8907,660.6109,677.0794,792.5274,812,852.0034,864.7995,883.7464,897.5401,899.7791,92
2.1605,924.5309,934.5052,953.8258,958.4649,990.1287,996.5556,999.9383,1009.8924,1011.

4762,1020.9623,1023.4906,1036.8696,1038.2629,1047.099,1049.7442,1055.6927,1062.9398,1066.8555,1068.5366,1073.4148,1076.0926,1077.6749,1080.353,1096.8702,1100.3906,1104.4001,1107.1314,1116.9159,1122.2826,1127.7296,1130.7897,1131.9012,1135.2889,1142.7744,1148.4956,1152.3895,1157.9887,1162.4323,1166.7108,1195.9188,1199.0789,1199.3468,1213.9901,1222.0333,1232.8969,1244.2712,1245.7235,1253.9318,1258.5373,1262.6231,1266.8441,1273.7015,1278.7278,1282.3789,1287.745,1298.2207,1314.0828,1317.2183,1319.5203,1333.3415,1334.936,1349.7182,1352.8291,1357.1673,1359.3201,1364.2337,1364.4731,1366.3161,1373.5072,1381.5856,1382.9507,1387.1993,1398.2042,1410.8802,1421.1647,1421.8074,1429.3014,1429.6284,1441.078,1441.3393,1445.2743,1449.0722,1449.826,1452.0163,1454.3976,1459.4698,1460.6057,1464.7556,1469.2651,1475.2136,1488.2035,1505.6014,1507.3222,1513.1807,1517.677,1522.7801,1524.4999,1530.2464,1535.261,1535.4714,1536.3456,1543.0746,1689.9878,1704.8638,1724.3227,1775.9555,2942.3639,2951.4095,2977.7379,2989.3967,2989.6438,2996.9195,2997.1207,2997.9183,3013.5382,3015.0569,3018.8402,3021.1815,3029.122,3029.9158,3033.0692,3042.4825,3043.1103,3047.8777,3053.5146,3060.8171,3091.6285,3095.9818,3099.6647,3106.3797,3115.1104,3116.0351,3124.2779,3125.6132,3135.9151,3137.9946,3140.7004,3152.9996,3162.9576,3173.6105,3185.3507,3721.0926,3721.2031,3722.809,3729.1168,3733.9826,3735.9741,3737.5048

Table S3. Cartesian coordinates for conformers of 6*R*-**1**

C1				C2			
C	6.219452	-1.330571	-0.452477	H	-0.02336	0.326183	0.328697
C	6.052854	-1.346937	-1.981605	C	0.709016	1.122343	0.524409
C	7.71542	-1.125481	-0.131123	C	2.02666	0.781338	-0.094519
H	6.670596	-2.138313	-2.422389	O	0.237339	2.335954	-0.088075
H	6.367473	-0.392944	-2.42279	H	0.767977	1.261892	1.611798
H	5.014255	-1.523636	-2.279149	C	3.175267	0.427595	0.520049
C	5.734396	-2.671834	0.140027	H	2.013011	0.812746	-1.183583
C	5.394237	-0.15865	0.180378	C	-1.02632	2.741359	0.328824
H	8.31055	-1.946297	-0.547959	C	3.345518	0.333272	2.017794
H	8.082791	-0.191221	-0.573138	C	4.334001	0.10021	-0.320814
H	7.908048	-1.087458	0.945476	C	-1.249367	4.15549	-0.21019
C	5.682268	-2.703252	1.664161	O	-2.008712	1.858702	-0.199251
H	6.364317	-3.504097	-0.192832	H	-1.091685	2.741993	1.433942
H	4.718726	-2.894753	-0.220095	H	4.195897	0.941038	2.349661
C	5.351622	-0.24051	1.705297	H	3.553774	-0.700046	2.322907
C	3.964958	-0.072367	-0.315233	H	2.462315	0.666665	2.56571
H	5.892695	0.782207	-0.094463	C	5.5424	-0.305521	0.105486
C	5.453585	-1.41936	2.351102	H	4.170581	0.197156	-1.395068
O	5.802052	-3.749723	2.286817	C	-2.656778	4.611341	0.149106
C	5.15008	1.055942	2.439157	O	-0.341367	5.089318	0.356477
C	3.439158	1.00317	-0.926449	H	-1.145492	4.113322	-1.304768
H	3.342484	-0.943672	-0.119527	C	-3.339843	2.201336	0.199778

H	5.395997	-1.473489	3.436089	C	6.716169	-0.599657	-0.806835
H	4.266725	1.584969	2.060224	H	5.735351	-0.40664	1.172111
H	6.008281	1.724057	2.281181	C	-3.69331	3.599336	-0.334577
H	5.029942	0.900739	3.51505	O	-2.986886	5.862528	-0.439934
C	2.06064	1.148067	-1.412041	H	-2.728956	4.679995	1.247462
H	4.087369	1.86187	-1.107363	H	0.551336	4.767465	0.1489
C	1.097886	0.008822	-1.165299	C	-4.27791	1.137882	-0.337961
C	1.732732	2.285534	-2.065892	H	-3.414001	2.212609	1.298948
H	1.157416	-0.321372	-0.122057	C	7.27045	-2.05984	-0.684355
H	1.344279	-0.860763	-1.788576	C	7.791103	0.449586	-0.529809
H	0.059026	0.287149	-1.351961	H	6.368382	-0.463858	-1.841131
C	0.418057	2.633702	-2.697923	O	-4.989793	3.945473	0.122969
H	2.499802	3.053974	-2.165513	H	-3.660291	3.574263	-1.436344
O	-0.250353	3.735431	-2.03879	H	-2.295478	6.488974	-0.169573
H	-0.264716	1.783021	-2.750315	O	-4.004801	-0.084858	0.346513
H	0.577327	3.00092	-3.716834	H	-5.312883	1.451529	-0.170336
C	-0.753377	3.441461	-0.774818	H	-4.111562	1.009498	-1.417671
C	-1.295895	4.744756	-0.186831	C	6.122944	-3.082241	-0.748248
O	-1.809238	2.491689	-0.897939	C	8.230625	-2.33641	-1.861146
H	0.033852	3.025819	-0.121753	C	8.025883	-2.205685	0.654851
C	-1.941498	4.452552	1.161047	C	7.598757	1.791855	-1.178708
O	-0.266679	5.69953	0.034554	C	8.8342	0.191751	0.284374
H	-2.055518	5.13351	-0.881391	H	-5.102642	4.889436	-0.080715
C	-2.368839	2.117131	0.36348	C	-4.62501	-1.211769	-0.180688
C	-2.993762	3.35303	1.032322	H	6.521068	-4.10311	-0.708863
O	-2.592505	5.592699	1.707286	H	5.555981	-2.980771	-1.682008
H	-1.157061	4.098962	1.850873	H	5.419358	-2.961329	0.081661
H	0.162048	5.846467	-0.824417	H	8.644948	-3.348458	-1.785037
C	-3.396361	1.029381	0.120486	H	7.700845	-2.265825	-2.819011
H	-1.582819	1.716278	1.022906	H	9.069428	-1.63402	-1.887457
O	-3.492202	2.97762	2.305707	C	9.067788	-1.121362	0.911352
H	-3.806294	3.72889	0.388917	H	8.527001	-3.177458	0.724665
H	-1.931623	6.304128	1.733599	H	7.313098	-2.169359	1.492456
O	-2.720647	-0.141262	-0.346868	H	6.602941	2.193745	-0.953756
H	-3.919691	0.820529	1.058094	H	7.661762	1.702058	-2.272228
H	-4.124593	1.365038	-0.632399	H	8.350626	2.515041	-0.850273
H	-3.711675	3.807194	2.762762	H	9.581356	0.95244	0.500285
C	-3.540164	-1.255662	-0.506664	C	-3.957293	-2.429309	0.460068
C	-2.755354	-2.288034	-1.317174	O	-6.010685	-1.193057	0.135197
O	-3.868707	-1.789633	0.768989	H	-4.5006	-1.244533	-1.279565

H	-4.4692	-0.981749	-1.040751	O	10.041081	-1.326039	1.624394
C	-3.578038	-3.56378	-1.440515	C	-4.650711	-3.696013	-0.021574
O	-2.480938	-1.825794	-2.632795	O	-2.585599	-2.517902	0.100274
H	-1.825618	-2.50349	-0.769235	H	-4.076798	-2.338183	1.550303
C	-4.728108	-2.940927	0.695895	C	-6.721019	-2.330851	-0.385034
C	-4.010602	-4.063906	-0.063416	C	-6.154851	-3.61499	0.23437
O	-2.849814	-4.615895	-2.06256	O	-4.171802	-4.860187	0.641868
H	-4.484877	-3.335707	-2.024849	H	-4.489867	-3.788266	-1.108525
H	-2.03334	-0.969109	-2.536268	H	-2.194359	-1.654183	0.314597
C	-5.075005	-3.302225	2.135476	C	-8.19152	-2.093444	-0.0619
H	-5.654509	-2.675968	0.162266	H	-6.599156	-2.371906	-1.479006
O	-4.897002	-5.164594	-0.180152	O	-6.826874	-4.723966	-0.340055
H	-3.105708	-4.347716	0.498998	H	-6.316126	-3.581482	1.324572
H	-2.551111	-4.277768	-2.922616	H	-3.210015	-4.881614	0.508687
O	-5.73219	-2.234682	2.7941	O	-8.66237	-0.893855	-0.649218
H	-5.756264	-4.156257	2.137888	H	-8.787488	-2.913312	-0.469497
H	-4.151753	-3.596377	2.661424	H	-8.31893	-2.085728	1.03325
H	-4.474925	-5.782873	-0.800206	H	-6.333924	-5.511622	-0.054572
H	-5.148991	-1.46408	2.698102	H	-8.050756	-0.198854	-0.355505

C3				C4			
C	6.426608	0.167348	-1.547116	C	5.054589	-2.162005	1.704723
C	5.376887	-0.045038	-2.651221	C	3.776871	-2.431881	2.519201
C	7.653066	0.86491	-2.173734	C	5.491768	-0.695384	1.902217
H	5.801204	-0.638471	-3.469786	H	3.98384	-2.324118	3.590293
H	5.04604	0.913424	-3.070207	H	3.408259	-3.452819	2.355729
H	4.490024	-0.567654	-2.27896	H	2.972427	-1.734207	2.264721
C	6.844456	-1.196446	-0.954654	C	6.154013	-3.121523	2.208224
C	5.850749	1.07097	-0.404179	C	4.814305	-2.409213	0.169352
H	8.084065	0.240012	-2.964599	C	6.674782	-0.279743	1.036675
H	7.365709	1.821814	-2.626481	H	5.746115	-0.490137	2.947687
H	8.439865	1.062512	-1.439128	H	4.655496	-0.029507	1.642922
C	7.743955	-1.100018	0.27344	H	6.360865	-2.940545	3.269368
H	7.357566	-1.813444	-1.700544	H	5.834042	-4.165811	2.105907
H	5.950443	-1.7628	-0.653205	H	7.093787	-3.002373	1.660576
C	6.777159	1.125399	0.808919	C	3.49618	-1.83626	-0.33234
C	4.478647	0.651605	0.084014	C	5.988408	-1.941084	-0.677559
H	5.757257	2.090728	-0.805043	H	4.735458	-3.500365	0.041121
C	7.605803	0.10549	1.109783	C	6.810756	-0.955047	-0.267866
O	8.527967	-1.993792	0.563806	O	7.461248	0.587655	1.391833

C	6.682702	2.355435	1.667989	C	3.336571	-0.696557	-1.027197
C	3.392176	1.44263	0.063701	H	2.621131	-2.431239	-0.080691
H	4.411428	-0.349604	0.506113	C	6.173059	-2.630677	-2.00183
H	8.242593	0.138533	1.991243	H	7.645432	-0.624752	-0.882667
H	5.644162	2.539887	1.969938	C	2.061799	-0.145005	-1.506901
H	7.009143	3.241938	1.106358	H	4.220199	-0.114414	-1.286248
H	7.299538	2.273133	2.567417	H	5.250917	-2.588778	-2.594659
C	2.048907	1.093395	0.542391	H	6.402577	-3.694747	-1.851831
H	3.492057	2.447047	-0.350633	H	6.983785	-2.184178	-2.584671
C	1.83698	-0.291578	1.107274	C	0.789988	-0.888456	-1.167544
C	1.082621	2.031668	0.463842	C	2.098162	0.992491	-2.237462
H	2.124078	-1.055552	0.373677	H	0.765247	-1.13496	-0.09985
H	2.469607	-0.447713	1.990731	H	0.727911	-1.838514	-1.714254
H	0.80273	-0.476768	1.403237	H	-0.108604	-0.306285	-1.379359
C	-0.341414	1.922994	0.910057	C	0.946386	1.710759	-2.875572
H	1.340131	3.008478	0.054758	H	3.069038	1.460966	-2.399968
O	-1.149856	2.65932	-0.028452	O	0.690245	3.005733	-2.282029
H	-0.694576	0.889744	0.967771	H	0.024188	1.12574	-2.864781
H	-0.47337	2.379346	1.907923	H	1.183243	1.947024	-3.917872
C	-2.494888	2.723423	0.303934	C	0.161351	2.964402	-0.995587
C	-3.152022	3.774833	-0.591282	C	0.091189	4.404065	-0.48458
O	-3.101452	1.453191	0.077825	O	-1.150019	2.405629	-1.044393
H	-2.621924	3.000986	1.368574	H	0.791402	2.351939	-0.327277
C	-4.645149	3.817246	-0.29321	C	-0.569009	4.4126	0.887892
O	-2.635396	5.075969	-0.349761	O	1.380238	4.982057	-0.335775
H	-2.992966	3.470951	-1.636637	H	-0.524	4.979914	-1.192205
C	-4.487953	1.418729	0.426855	C	-1.757848	2.304256	0.245357
C	-5.265279	2.428428	-0.432652	C	-1.924941	3.711211	0.84361
O	-5.349043	4.679697	-1.177058	O	-0.796011	5.730535	1.371031
H	-4.779717	4.152477	0.748911	H	0.081365	3.861009	1.587105
H	-1.679551	5.02909	-0.515119	H	1.810489	4.928975	-1.204841
C	-4.982188	-0.005437	0.216024	C	-3.090474	1.599496	0.085931
H	-4.61448	1.685924	1.488988	H	-1.1229	1.706001	0.917988
O	-6.618511	2.43081	-0.009364	O	-2.475445	3.589975	2.144974
H	-5.185873	2.124599	-1.489615	H	-2.592591	4.295329	0.188978
H	-4.913421	5.546494	-1.126386	H	0.06144	6.186537	1.347655
O	-4.291723	-0.91716	1.07663	O	-2.847317	0.250473	-0.323118
H	-6.046548	-0.053407	0.459416	H	-3.619186	1.620044	1.043408
H	-4.851821	-0.29846	-0.83186	H	-3.697318	2.114889	-0.672874
H	-7.037672	3.186842	-0.45422	H	-2.392242	4.468451	2.553302
C	-3.353515	-1.737886	0.453175	C	-3.987668	-0.543379	-0.406514
C	-2.43334	-2.290565	1.54212	C	-3.608813	-1.806374	-1.1812

O	-4.032355	-2.812692	-0.187717	O	-4.421672	-0.890244	0.902221
H	-2.77073	-1.17282	-0.291872	H	-4.798865	-0.005792	-0.932027
C	-1.456692	-3.286027	0.930206	C	-4.801722	-2.752301	-1.219909
O	-1.670022	-1.257185	2.161029	O	-3.251066	-1.511923	-2.524528
H	-3.062776	-2.803043	2.284034	H	-2.777277	-2.287834	-0.644554
C	-3.143782	-3.714137	-0.870109	C	-5.607551	-1.704222	0.907523
C	-2.203782	-4.366827	0.14989	C	-5.318547	-3.029019	0.190623
O	-0.670645	-3.947388	1.914956	O	-4.474339	-4.007872	-1.804154
H	-0.803891	-2.744388	0.225718	H	-5.609001	-2.268253	-1.794096
H	-2.309407	-0.583578	2.447241	H	-2.551268	-0.839421	-2.482414
C	-4.036074	-4.708488	-1.604324	C	-5.997054	-1.874771	2.371253
H	-2.547596	-3.156045	-1.609311	H	-6.419344	-1.176675	0.382316
O	-1.296889	-5.206294	-0.545391	O	-6.514775	-3.789532	0.151545
H	-2.808694	-4.94943	0.864084	H	-4.531424	-3.565735	0.74585
H	-0.230352	-3.257239	2.436961	H	-4.118284	-3.819204	-2.687854
O	-4.902865	-4.049816	-2.510228	O	-6.250611	-0.626449	2.989452
H	-3.413842	-5.393591	-2.184783	H	-6.915917	-2.462483	2.433076
H	-4.598711	-5.298483	-0.862042	H	-5.197682	-2.429426	2.89005
H	-0.630436	-5.479553	0.107244	H	-6.339373	-4.532452	-0.450285
H	-5.360776	-3.37154	-1.986933	H	-5.456476	-0.088357	2.837648

C5				C6			
C	6.10274	-1.275856	-0.578664	H	0.071564	-0.014344	0.611308
C	6.264558	-0.799757	-2.032828	C	0.859425	0.702707	0.88361
C	5.432283	-2.666125	-0.578938	C	2.196045	0.206397	0.432102
H	6.87529	-1.511244	-2.600958	O	0.602284	1.943651	0.20411
H	6.76109	0.177542	-2.080376	H	0.804297	0.862588	1.968626
H	5.295946	-0.709851	-2.535273	C	3.205433	-0.284118	1.181993
C	7.500929	-1.377932	0.069364	H	2.32667	0.242023	-0.648932
C	5.264112	-0.223726	0.235565	C	-0.626367	2.524213	0.499005
H	4.483042	-2.652502	-1.125106	C	3.164886	-0.402435	2.687161
H	6.083991	-3.395052	-1.074079	C	4.416346	-0.742908	0.487774
H	5.236385	-3.031382	0.434714	C	-0.620383	3.928941	-0.106936
C	7.467182	-1.718087	1.550975	O	-1.671303	1.749826	-0.077447
H	8.123406	-2.119793	-0.442282	H	-0.777604	2.583577	1.594182
H	8.015841	-0.408673	-0.026423	H	4.027279	0.102249	3.139465
C	5.346389	-0.44098	1.751008	H	3.21176	-1.453868	2.997966
C	3.832712	-0.125919	-0.241156	H	2.260672	0.028765	3.120837
H	5.731159	0.75826	0.047056	C	5.501432	-1.288255	1.064138

C	6.355966	-1.131599	2.317893	H	4.403481	-0.623001	-0.594893
O	8.33679	-2.393209	2.084868	C	-1.98046	4.575838	0.115468
C	4.284615	0.204862	2.601389	O	0.35262	4.766329	0.500895
C	3.318952	0.93899	-0.879577	H	-0.436274	3.823372	-1.186487
H	3.203127	-0.99252	-0.048763	C	-2.973639	2.277524	0.194028
H	6.402484	-1.270577	3.395822	C	6.765109	-1.750629	0.351797
H	3.293786	-0.223506	2.406085	H	5.517645	-1.423137	2.143418
H	4.202618	1.276087	2.37458	C	-3.098191	3.682519	-0.41827
H	4.50534	0.090392	3.666324	O	-2.096198	5.830412	-0.54296
C	1.946796	1.081454	-1.384233	H	-2.130193	4.703427	1.200734
H	3.971809	1.793393	-1.064622	H	1.212247	4.333626	0.369119
C	0.978326	-0.052766	-1.136977	C	-3.995766	1.316843	-0.38294
C	1.629103	2.2125	-2.053854	H	-3.133166	2.34956	1.281824
H	1.019505	-0.367918	-0.088101	C	7.965198	-0.743672	0.497136
H	1.23409	-0.931758	-1.743007	C	6.490563	-2.108879	-1.101211
H	-0.056927	0.224004	-1.34427	H	7.075776	-2.676467	0.861527
C	0.322191	2.557383	-2.703772	O	-4.370984	4.211154	-0.085204
H	2.39916	2.977974	-2.152902	H	-2.981038	3.602428	-1.511605
O	-0.350658	3.667341	-2.063297	H	-1.347278	6.371955	-0.243721
H	-0.362108	1.707844	-2.754977	O	-3.936739	0.102775	0.366019
H	0.493496	2.913912	-3.724503	H	-4.992111	1.764569	-0.315182
C	-0.860858	3.390663	-0.79843	H	-3.761638	1.118788	-1.439166
C	-1.408357	4.701369	-0.231863	C	8.084185	-0.223286	1.940667
O	-1.915123	2.4378	-0.914063	C	7.727451	0.442795	-0.46024
H	-0.07718	2.985023	-0.134966	C	9.281935	-1.465665	0.139515
C	-2.060488	4.427075	1.116712	C	5.84207	-3.443574	-1.348784
O	-0.381905	5.660639	-0.018086	C	6.80123	-1.270734	-2.109988
H	-2.164892	5.079151	-0.935788	H	-4.343394	5.151173	-0.331956
C	-2.480255	2.080126	0.349579	C	-4.609106	-0.977307	-0.193208
C	-3.110289	3.324091	0.998338	H	8.967245	0.419088	2.037483
O	-2.716096	5.573528	1.643651	H	8.197822	-1.049839	2.654055
H	-1.278875	4.084552	1.815299	H	7.210448	0.365751	2.237691
H	0.051121	5.797293	-0.876591	C	7.463918	0.030799	-1.903302
C	-3.505042	0.987411	0.117559	H	8.573001	1.139156	-0.454124
H	-1.696822	1.68982	1.018411	H	6.847005	1.009516	-0.123162
O	-3.613935	2.965264	2.274537	H	10.127021	-0.770197	0.201252
H	-3.920508	3.689621	0.346161	H	9.476421	-2.287271	0.839944
H	-2.056655	6.286535	1.663204	H	9.266895	-1.882569	-0.872034

O	-2.825955	-0.189037	-0.330751	H	4.920039	-3.543096	-0.762792
H	-4.031481	0.790963	1.056099	H	6.507019	-4.258344	-1.030233
H	-4.230838	1.311164	-0.642759	H	5.601951	-3.590678	-2.405668
H	-3.837088	3.80067	2.719045	H	6.59672	-1.534201	-3.145593
C	-3.640051	-1.311422	-0.459088	C	-4.142341	-2.226957	0.554675
C	-2.859377	-2.353708	-1.260868	O	-6.011664	-0.80948	-0.035133
O	-3.94749	-1.823805	0.830811	H	-4.370532	-1.067345	-1.269763
H	-4.578058	-1.053744	-0.98532	O	7.764108	0.752036	-2.845055
C	-3.674517	-3.637283	-1.348718	C	-4.90437	-3.438355	0.036239
O	-2.606223	-1.914889	-2.58853	O	-2.755419	-2.462025	0.354511
H	-1.920737	-2.552517	-0.721763	H	-4.37016	-2.079975	1.62134
C	-4.800399	-2.981511	0.790771	C	-6.773498	-1.893027	-0.596694
C	-4.085513	-4.113969	0.043219	C	-6.411243	-3.199906	0.120412
O	-2.946887	-4.696226	-1.959918	O	-4.620896	-4.616131	0.783125
H	-4.59045	-3.426808	-1.92535	H	-4.637256	-3.59008	-1.022725
H	-2.163435	-1.053433	-2.514831	H	-2.307139	-1.627882	0.574914
C	-5.125814	-3.3178	2.241394	C	-8.238427	-1.499609	-0.446979
H	-5.735465	-2.732496	0.264699	H	-6.538946	-1.990906	-1.66848
O	-4.965486	-5.222651	-0.040701	O	-7.126919	-4.260486	-0.49156
H	-3.171198	-4.3809	0.598765	H	-6.684939	-3.105896	1.184344
H	-2.664583	-4.373931	-2.831528	H	-3.657611	-4.737116	0.758051
O	-5.780969	-2.242322	2.888789	O	-8.517842	-0.284375	-1.118285
H	-5.801328	-4.175944	2.269066	H	-8.867466	-2.272821	-0.894159
H	-4.193596	-3.596272	2.760182	H	-8.481958	-1.434949	0.626438
H	-4.548955	-5.848404	-0.65702	H	-6.74962	-5.080168	-0.1301
H	-5.20537	-1.469365	2.768772	H	-7.871652	0.357506	-0.78086

C7			
C	7.633005	-0.590499	0.105189
C	7.744496	0.235178	1.398385
C	8.918692	-1.431701	-0.04411
H	8.636436	0.872089	1.36687
H	7.831442	-0.417336	2.276209
H	6.873183	0.880657	1.548956
C	7.480527	0.35533	-1.106334
C	6.396537	-1.549348	0.168566
H	9.796444	-0.778129	-0.107977

H	9.056062	-2.088162	0.823886
H	8.903688	-2.058202	-0.941252
C	7.175366	-0.352875	-2.422504
H	8.380438	0.963068	-1.251683
H	6.658811	1.064673	-0.926227
C	6.120428	-2.224536	-1.173524
C	5.114689	-0.873478	0.611926
H	6.622916	-2.335878	0.903037
C	6.458618	-1.638049	-2.339329
O	7.491558	0.135055	-3.49923
C	5.418083	-3.552668	-1.122955
C	4.381511	-1.246335	1.671825
H	4.769185	-0.04445	-0.005306
H	6.235097	-2.11126	-3.293019
H	4.495662	-3.483767	-0.532962
H	6.051536	-4.303111	-0.629565
H	5.167383	-3.919294	-2.122358
C	3.107847	-0.632745	2.10755
H	4.762694	-2.046325	2.310466
C	2.916479	-0.598859	3.607804
C	2.213195	-0.184235	1.206808
H	3.749571	-0.074319	4.092543
H	2.908664	-1.619057	4.014863
H	1.986765	-0.115628	3.915063
C	0.892398	0.470177	1.467784
H	2.430763	-0.318059	0.148882
O	0.83435	1.667974	0.67151
H	0.742974	0.728954	2.524201
H	0.068354	-0.18997	1.163055
C	-0.366809	2.363598	0.757643
C	-0.159188	3.713369	0.068326
O	-1.391385	1.631808	0.095346
H	-0.654079	2.517945	1.815666
C	-1.474516	4.479835	0.061986
O	0.797108	4.51502	0.747171
H	0.153464	3.512247	-0.967203
C	-2.666748	2.278424	0.153392
C	-2.5875	3.638717	-0.559367
O	-1.393914	5.686054	-0.686113
H	-1.75193	4.700755	1.106348
H	1.621836	4.002295	0.768548
C	-3.685845	1.360412	-0.493898
H	-2.959107	2.444527	1.202613

O	-3.841492	4.288955	-0.436535
H	-2.33713	3.467344	-1.619297
H	-0.647084	6.189922	-0.322821
O	-3.83122	0.204708	0.331754
H	-4.639256	1.88912	-0.589773
H	-3.335687	1.066087	-1.494155
H	-3.700156	5.202332	-0.738267
C	-4.541972	-0.846698	-0.234877
C	-4.29056	-2.075974	0.639475
O	-5.929288	-0.537834	-0.254915
H	-4.194691	-1.037548	-1.267817
C	-5.108922	-3.245809	0.111485
O	-2.92118	-2.454475	0.619449
H	-4.619527	-1.830034	1.660543
C	-6.729386	-1.583965	-0.833914
C	-6.58381	-2.862285	0.001577
O	-5.031199	-4.386425	0.95901
H	-4.742004	-3.499687	-0.896829
H	-2.417307	-1.653046	0.841015
C	-8.154443	-1.045548	-0.883816
H	-6.387627	-1.782307	-1.862107
O	-7.330272	-3.89386	-0.62305
H	-6.963734	-2.664631	1.017588
H	-4.088308	-4.598008	1.055885
O	-8.232827	0.13713	-1.658824
H	-8.804602	-1.787846	-1.352654
H	-8.508516	-0.878843	0.146987
H	-7.080433	-4.716167	-0.168873
H	-7.567143	0.739574	-1.288237

Table S4. Cartesian coordinates for conformers of 6*S*-**1**

**C1**				**C2**			
H	-0.030081	0.291075	0.274509	C	-7.347552	-1.918036	-0.408545
C	0.70108	1.082164	0.494296	C	-7.006135	-2.623499	-1.733144
C	2.025499	0.753475	-0.117239	C	-6.483866	-2.50726	0.726296
O	0.237963	2.30852	-0.09824	H	-7.274632	-3.684739	-1.674217
H	0.748595	1.199171	1.584957	H	-7.564683	-2.188134	-2.571922
C	3.163278	0.372836	0.501226	H	-5.938243	-2.563224	-1.966668
H	2.027262	0.821157	-1.204739	C	-8.845797	-2.148507	-0.116797
C	-1.01804	2.722833	0.332465	C	-7.068018	-0.372338	-0.498028
C	3.311047	0.226585	1.99718	C	-6.646324	-1.792756	2.062114
C	4.332801	0.068697	-0.333399	H	-6.695849	-3.570732	0.881044
C	-1.231292	4.142995	-0.194702	H	-5.421839	-2.432307	0.44979

O	-2.013663	1.853729	-0.193192	H	-9.054168	-3.219989	-0.016102
H	-1.073709	2.715973	1.438127	H	-9.464213	-1.7659	-0.938166
H	4.154682	0.824201	2.363234	H	-9.17098	-1.657965	0.805654
H	3.517549	-0.816264	2.268946	C	-5.750423	-0.037527	-1.183523
H	2.418922	0.539072	2.542952	C	-7.184903	0.310935	0.856356
C	5.536427	-0.346569	0.097196	H	-7.856968	0.046995	-1.142446
H	4.185695	0.203297	-1.405956	C	-6.969023	-0.354264	2.008475
C	-2.631408	4.610392	0.178139	O	-6.49411	-2.374164	3.127857
O	-0.31004	5.06391	0.371585	C	-4.607018	0.326632	-0.577677
H	-1.136051	4.107518	-1.290301	H	-5.772575	-0.122081	-2.267797
C	-3.338072	2.207414	0.21857	C	-7.542706	1.772122	0.838165
C	6.703816	-0.672484	-0.812539	H	-7.05137	0.144582	2.971856
H	5.706339	-0.506545	1.160549	C	-3.335221	0.637527	-1.2446
C	-3.681571	3.612044	-0.304538	H	-4.593314	0.408195	0.508573
O	-2.95311	5.868762	-0.400062	H	-6.851191	2.334123	0.198328
H	-2.694831	4.672321	1.277448	H	-8.547879	1.916	0.418143
H	0.578025	4.735867	0.154017	H	-7.52458	2.208972	1.840744
C	-4.290564	1.155905	-0.31745	C	-3.293587	0.572975	-2.753047
H	-3.403199	2.212911	1.318366	C	-2.275651	0.950114	-0.468949
C	7.988963	0.178933	-0.532986	H	-4.072815	1.209348	-3.189806
C	6.97137	-2.172297	-0.70201	H	-3.481416	-0.448971	-3.106196
H	6.384525	-0.459991	-1.843112	H	-2.333559	0.891432	-3.163493
O	-4.971174	3.968094	0.164659	C	-0.875966	1.263608	-0.891923
H	-3.656745	3.593659	-1.406654	H	-2.413009	0.96104	0.611794
H	-2.252904	6.486102	-0.131375	O	-0.453318	2.436494	-0.174171
O	-4.024067	-0.072271	0.359801	H	-0.783064	1.443857	-1.971089
H	-5.321361	1.478493	-0.141168	H	-0.201934	0.434683	-0.632747
H	-4.132886	1.030897	-1.398819	C	0.85334	2.835287	-0.434436
C	7.638137	1.673289	-0.433112	C	1.043833	4.210716	0.207496
C	8.982501	-0.007454	-1.699786	O	1.761385	1.901949	0.137333
C	8.634893	-0.295275	0.787288	H	1.032352	2.897539	-1.525311
C	6.0717	-3.072992	-1.501399	C	2.48788	4.654426	0.019164
C	7.939299	-2.658092	0.100831	O	0.213694	5.195924	-0.391068
H	-5.075943	4.914501	-0.031879	H	0.82836	4.108018	1.281543
C	-4.641129	-1.195282	-0.178955	C	3.131614	2.23637	-0.105439
H	8.547332	2.267802	-0.284231	C	3.452825	3.593033	0.543539
H	7.154603	2.026951	-1.352251	O	2.777172	5.862138	0.7114
H	6.958492	1.880165	0.399785	H	2.672449	4.784223	-1.0604
H	9.898412	0.566053	-1.515616	H	-0.700581	4.882699	-0.293058
H	8.548461	0.353233	-2.640366	C	3.992352	1.122338	0.458577
H	9.26756	-1.054689	-1.839975	H	3.31648	2.309052	-1.189144
C	8.856513	-1.802118	0.874007	O	4.794725	3.935104	0.239206
H	9.598655	0.197172	0.957588	H	3.308068	3.505776	1.633

H	7.995235	-0.016251	1.638126	H	2.124912	6.517257	0.413157
H	5.017018	-2.855765	-1.291105	O	3.764386	-0.052931	-0.319126
H	6.218597	-2.905314	-2.577559	H	5.044455	1.419806	0.410372
H	6.260281	-4.129381	-1.290171	H	3.718568	0.937813	1.507797
H	8.106221	-3.728783	0.196998	H	4.902962	4.862364	0.510725
C	-3.976775	-2.416926	0.457496	C	4.318034	-1.221013	0.192563
O	-6.028684	-1.178989	0.128482	C	3.683114	-2.385589	-0.56873
H	-4.509935	-1.220383	-1.277251	O	5.725324	-1.21653	-0.007575
O	9.741164	-2.278728	1.572466	H	4.102302	-1.311339	1.273844
C	-4.666538	-3.680499	-0.037184	C	4.310872	-3.692467	-0.10469
O	-2.602706	-2.501672	0.105703	O	2.285168	-2.461605	-0.327802
H	-4.10306	-2.333444	1.547576	H	3.894386	-2.237306	-1.638652
C	-6.735385	-2.313605	-0.403579	C	6.369147	-2.397711	0.502542
C	-6.172254	-3.601694	0.210393	C	5.832112	-3.632041	-0.233187
O	-4.191273	-4.849128	0.621046	O	3.866932	-4.807456	-0.869405
H	-4.499173	-3.765152	-1.123773	H	4.059498	-3.840456	0.958614
H	-2.214536	-1.63833	0.327187	H	1.930174	-1.576627	-0.517253
C	-8.207855	-2.07893	-0.087452	C	7.865318	-2.176863	0.313363
H	-6.607071	-2.347159	-1.497076	H	6.157088	-2.496619	1.578881
O	-6.84048	-4.706947	-0.375499	O	6.434238	-4.786589	0.328765
H	-6.339908	-3.575623	1.29983	H	6.082816	-3.541485	-1.303103
H	-3.228605	-4.869044	0.494229	H	2.897581	-4.81808	-0.810444
O	-8.675599	-0.875501	-0.669347	O	8.308412	-1.021988	1.003002
H	-8.801148	-2.896183	-0.504107	H	8.410659	-3.031135	0.72121
H	-8.341668	-2.07871	1.006965	H	8.082246	-2.11255	-0.765773
H	-6.349215	-5.496417	-0.092142	H	5.948448	-5.545105	-0.036769
H	-8.065733	-0.182441	-0.367517	H	7.733484	-0.299078	0.702561

**C3**				**C4**			
C	6.464371	-2.094579	-1.20886	C	6.793471	-2.028422	0.224862
C	5.22797	-2.982035	-1.432714	C	6.33143	-2.662593	1.548038
C	7.724547	-2.970946	-1.373869	C	8.321438	-2.213706	0.106831
H	5.270557	-3.449157	-2.423746	H	6.608266	-3.723076	1.578256
H	5.178453	-3.785043	-0.686826	H	6.803699	-2.169874	2.407168
H	4.295583	-2.411952	-1.368521	H	5.246851	-2.592631	1.679401
C	6.480843	-0.950462	-2.246242	C	6.087487	-2.72217	-0.960946
C	6.451596	-1.482235	0.23345	C	6.45898	-0.498788	0.200666
H	7.764482	-3.393977	-2.384371	H	8.576999	-3.279697	0.102646
H	7.711342	-3.807207	-0.664182	H	8.835379	-1.752444	0.959091
H	8.647921	-2.406262	-1.212231	H	8.724189	-1.770816	-0.8093
C	7.562181	0.098662	-2.008226	C	6.327166	-2.055861	-2.311936
H	6.605455	-1.338142	-3.263315	H	6.390249	-3.771751	-1.044537

H	5.515205	-0.422903	-2.236643	H	4.999537	-2.730502	-0.796325
C	7.538981	-0.426038	0.420467	C	6.696257	0.122679	-1.174428
C	5.128121	-0.856387	0.625148	C	5.037385	-0.171547	0.611456
H	6.647883	-2.300982	0.940801	H	7.128848	-0.00075	0.916659
C	8.00202	0.300724	-0.615973	C	6.596888	-0.606819	-2.303675
O	8.026624	0.758834	-2.92791	O	6.275868	-2.69097	-3.35659
C	8.030271	-0.214335	1.825288	C	7.006339	1.593452	-1.199952
C	4.389459	-1.230364	1.680859	C	4.704799	0.647496	1.621279
H	4.795196	-0.015715	0.016882	H	4.246763	-0.624854	0.014138
H	8.756992	1.070496	-0.471221	H	6.741307	-0.152663	-3.281597
H	7.190622	-0.024972	2.505789	H	6.246085	2.161806	-0.649825
H	8.537657	-1.116325	2.195147	H	7.967045	1.791628	-0.704319
H	8.729651	0.623916	1.89095	H	7.061374	1.980416	-2.221397
C	3.104935	-0.630342	2.104159	C	3.333378	1.030045	2.017688
H	4.769275	-2.028887	2.322092	H	5.504938	1.051448	2.245531
C	2.893129	-0.611177	3.602015	C	3.134846	1.199051	3.507848
C	2.217996	-0.180882	1.1966	C	2.373198	1.20548	1.089336
H	3.714612	-0.08311	4.102737	H	3.927402	1.831985	3.927396
H	2.889051	-1.63488	4.000148	H	3.201033	0.232114	4.023641
H	1.955103	-0.13895	3.900958	H	2.174404	1.650241	3.767204
C	0.889236	0.461756	1.446364	C	0.926766	1.510455	1.313613
H	2.449215	-0.303121	0.140186	H	2.64262	1.106334	0.039182
O	0.830371	1.661738	0.653666	O	0.563063	2.59755	0.444445
H	0.727378	0.715704	2.502124	H	0.703342	1.783114	2.353728
H	0.073261	-0.20362	1.131346	H	0.307639	0.638568	1.058407
C	-0.36977	2.3584	0.744221	C	-0.774521	2.971558	0.511395
C	-0.162763	3.709197	0.05661	C	-0.92031	4.274174	-0.277402
O	-1.397043	1.628712	0.083719	O	-1.585029	1.957602	-0.070205
H	-0.653816	2.511222	1.803333	H	-1.084311	3.12771	1.56289
C	-1.477485	4.476814	0.054996	C	-2.386704	4.681901	-0.30355
O	0.795694	4.509206	0.734102	O	-0.191912	5.339198	0.316979
H	0.146877	3.509548	-0.980125	H	-0.574916	4.080299	-1.303891
C	-2.671561	2.276563	0.146833	C	-2.982102	2.264681	-0.02385
C	-2.593104	3.637706	-0.564329	C	-3.255211	3.542993	-0.833842
O	-1.397753	5.68401	-0.691554	O	-2.61973	5.8096	-1.137545
H	-1.751705	4.696481	1.100469	H	-2.703251	4.904046	0.729325
H	1.621148	3.997456	0.750774	H	0.734498	5.049882	0.358209
C	-3.693833	1.360416	-0.498091	C	-3.741481	1.07307	-0.574723
H	-2.960209	2.441581	1.197249	H	-3.298136	2.431308	1.018391
O	-3.846063	4.289049	-0.437032	O	-4.631929	3.863966	-0.724433
H	-2.345959	3.467506	-1.625217	H	-2.977699	3.360465	-1.885115
H	-0.649121	6.186376	-0.32985	H	-2.024124	6.512141	-0.828914
O	-3.837443	0.203883	0.326653	O	-3.587625	-0.013162	0.338975

H	-4.64704	1.890199	-0.590037	H	-4.797565	1.337705	-0.685734
H	-3.347454	1.066976	-1.499934	H	-3.334362	0.797386	-1.558621
H	-3.704502	5.202883	-0.737286	H	-4.728728	4.757862	-1.094347
C	-4.550443	-0.846557	-0.238971	C	-4.033003	-1.246359	-0.122803
C	-4.296631	-2.077028	0.633013	C	-3.481999	-2.305486	0.832998
O	-5.93768	-0.537111	-0.254179	O	-5.454044	-1.279089	-0.119771
H	-4.206547	-1.036264	-1.273257	H	-3.664752	-1.432228	-1.149343
C	-5.117135	-3.245897	0.106165	C	-4.003019	-3.675293	0.42122
O	-2.927517	-2.456016	0.608007	O	-2.062269	-2.3476	0.797328
H	-4.622216	-1.832278	1.655452	H	-3.846726	-2.063269	1.842738
C	-6.740053	-1.582242	-0.831851	C	-5.988232	-2.528723	-0.591957
C	-6.592234	-2.86168	0.001524	C	-5.52825	-3.664038	0.331491
O	-5.037123	-4.387629	0.951906	O	-3.642612	-4.692399	1.348998
H	-4.753573	-3.498562	-0.903674	H	-3.599878	-3.91506	-0.576624
H	-2.422547	-1.655346	0.829791	H	-1.762184	-1.434789	0.945126
C	-8.165062	-1.04323	-0.876413	C	-7.501407	-2.350788	-0.634156
H	-6.401728	-1.779409	-1.861405	H	-5.623841	-2.722158	-1.613249
O	-7.341086	-3.892185	-0.622002	O	-6.014264	-4.890431	-0.189115
H	-6.968792	-2.665197	1.019014	H	-5.929972	-3.481297	1.341922
H	-4.094031	-4.599943	1.045243	H	-2.674888	-4.668516	1.427258
O	-8.245487	0.140548	-1.649542	O	-7.872725	-1.286757	-1.491809
H	-8.817034	-1.784641	-1.344139	H	-7.961192	-3.261677	-1.024529
H	-8.515724	-0.87779	0.155759	H	-7.870064	-2.191122	0.392649
H	-7.089936	-4.71518	-0.169786	H	-5.566525	-5.590348	0.315438
H	-7.578441	0.742245	-1.280192	H	-7.365209	-0.51565	-1.189783

**C5**			
H	-0.080168	0.264072	0.387496
C	0.65156	1.053048	0.613014
C	1.99377	0.693066	0.06073
O	0.225186	2.267565	-0.028854
H	0.659789	1.199024	1.701261
C	3.100412	0.308849	0.731099
H	2.038778	0.737207	-1.027019
C	-1.043538	2.702916	0.339565
C	3.186799	0.193403	2.234586
C	4.296319	-0.032125	-0.050488
C	-1.222677	4.111334	-0.229975
O	-2.024666	1.82924	-0.205308
H	-1.144398	2.723711	1.441848
H	4.031369	0.777444	2.619772
H	3.354455	-0.847857	2.537995

H	2.282807	0.540987	2.738059
C	5.472282	-0.466641	0.43292
H	4.193668	0.0853	-1.130316
C	-2.632384	4.598653	0.07547
O	-0.316737	5.038618	0.350491
H	-1.08422	4.048403	-1.319696
C	-3.361229	2.202693	0.145056
C	6.67783	-0.788828	-0.421084
H	5.595073	-0.602324	1.505845
C	-3.671588	3.597107	-0.424126
O	-2.919942	5.844873	-0.545315
H	-2.738556	4.688009	1.169593
H	0.576524	4.700003	0.174462
C	-4.30103	1.146447	-0.40383
H	-3.469653	2.234669	1.240972
C	7.854938	0.234026	-0.218182
C	7.120651	-2.247066	-0.254709
H	6.358945	-0.69025	-1.473039
O	-4.975365	3.974297	-0.014434
H	-3.603906	3.552079	-1.523661
H	-2.225471	6.46318	-0.264244
O	-4.068482	-0.069085	0.307855
H	-5.335114	1.479681	-0.272213
H	-4.10492	0.997712	-1.475881
C	7.436807	1.618982	-0.742033
C	8.253691	0.36068	1.267543
C	9.06761	-0.261407	-1.036328
C	6.045375	-3.272532	-0.010156
C	8.410686	-2.619218	-0.377237
H	-5.064651	4.916214	-0.238284
C	-4.685625	-1.196327	-0.222197
H	8.261564	2.332999	-0.632525
H	7.169256	1.579663	-1.805392
H	6.574873	2.011404	-0.192679
H	7.449628	0.809825	1.860267
H	9.132689	1.008132	1.364697
H	8.504475	-0.607948	1.712815
C	9.497802	-1.679746	-0.697095
H	9.930194	0.401794	-0.910826
H	8.810108	-0.250922	-2.107468
H	5.548817	-3.115614	0.955275
H	5.257813	-3.199319	-0.771769
H	6.453329	-4.287056	-0.027542

H	8.706119	-3.659815	-0.261665
C	-4.049227	-2.411658	0.453616
O	-6.079406	-1.159123	0.053252
H	-4.529885	-1.245231	-1.31645
O	10.668345	-2.033801	-0.738014
C	-4.74116	-3.677598	-0.031865
O	-2.668415	-2.517283	0.135523
H	-4.199608	-2.304977	1.53862
C	-6.78643	-2.296709	-0.472049
C	-6.251173	-3.578234	0.179555
O	-4.293726	-4.83789	0.659893
H	-4.550031	-3.785576	-1.11243
H	-2.27717	-1.652478	0.345724
C	-8.26291	-2.040645	-0.193473
H	-6.634372	-2.353316	-1.56153
O	-6.917904	-4.687784	-0.399817
H	-6.443118	-3.528993	1.264184
H	-3.328984	-4.871646	0.553525
O	-8.704957	-0.844337	-0.809102
H	-8.855531	-2.859919	-0.607101
H	-8.420805	-2.017359	0.897488
H	-6.44158	-5.476595	-0.090324
H	-8.094954	-0.151455	-0.507157

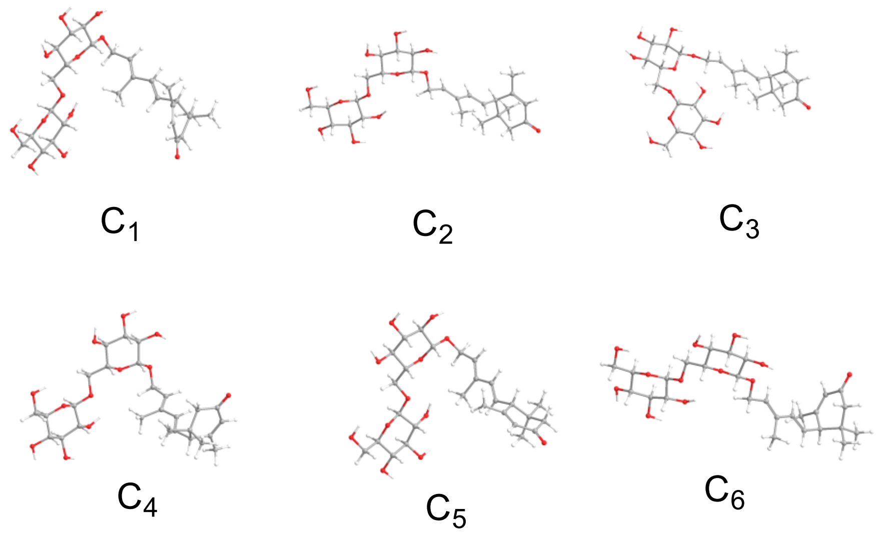

Fig. S37. B3LYP/6-31G(d) optimized lowest energy conformers for **6*R*-1**

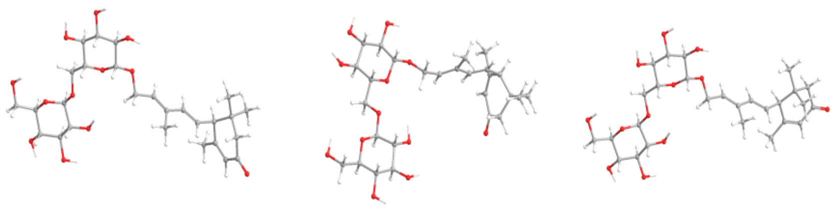

C_1 C_2 C_5

Fig. S38. B3LYP/6-31G(d) optimized lowest energy conformers for **6*S*-1**

ECD data

ECD simulation:

ECD spectrum of each conformation is simulated according to the overlapping Gaussian functions expressed as:

$$\Delta\varepsilon(E)=\frac{1}{2.296\times10^{-39}\sqrt{\pi}\sigma}\sum_{i}^{A}\Delta E_i R_i e^{[-(E-\Delta Ei)^2/\sigma^2]}$$

Where σ is half the bandwidth at 1/e peak height and expressed in energy units. The parameters ΔE_i and R_i are the excitation energies and rotational strengths for the transition *i*, respectively. The above function is converted to Δε, λ (wavelength) correlations as:

$$\Delta\varepsilon(\lambda)=\frac{1}{2.296\times10^{-39}\sqrt{\pi}\sigma}\sum_{i}^{A}\Delta E_i R_i e^{[-(1240/\lambda-\Delta Ei)^2/\sigma^2]}$$

and then simulation were accomplished by using the Excel 2003 and the Origin 7.0 software.

To get the final spectra, all the simulated spectra of conformations of each compound were averaged according to their energy and the Boltzmann distribution theory expressed as:

$$\frac{N_i^*}{N}=\frac{g_i e^{-\varepsilon_i/k_B T}}{\sum g_i e^{-\varepsilon_i/k_B T}}$$

Calculated ECD Data

Calculated ECD Data for 6*R*-1 in gas phase

State	**C1**	**C2**	**C3**

	Excitation energies(eV)	Rotatory Strengths*	Excitation energies(eV)	Rotatory Strengths*	Excitation energies(eV)	Rotatory Strengths*
1	3.622	-9.4438	3.6265	-10.3785	3.6269	-9.7819
2	5.1664	248.6198	5.1565	246.2745	5.1337	211.3141
3	5.7601	-4.72	5.7591	27.5174	5.7513	52.9349
4	5.8127	-226.3222	5.8285	-193.0554	5.8195	-198.7267
5	6.4171	-11.2563	6.5351	-43.2495	6.5445	-32.4383
6	6.4945	25.0252	6.5981	14.1664	6.6228	12.9377
7	6.7193	-25.6452	6.911	4.5416	6.834	6.2738
8	6.8512	-8.694	6.9478	-7.819	6.9083	5.8081
9	6.9122	5.046	7.0147	5.4805	7.0104	7.6329
10	7.0163	1.2624	7.1385	-0.3304	7.0674	-0.6109
11	7.1213	-8.5003	7.1583	-1.7953	7.1455	-8.4764
12	7.1561	3.6078	7.2438	16.1373	7.2586	13.8609
13	7.1779	-0.5007	7.4122	-15.1102	7.3172	0.0651
14	7.2825	10.0487	7.4903	-2.097	7.441	3.887
15	7.4251	22.0777	7.4938	-1.3821	7.4737	0.3586
16	7.5187	16.8579	7.5341	-1.9654	7.4954	-15.5441
17	7.5423	-3.692	7.6501	9.7188	7.6707	21.9182
18	7.6687	-5.769	7.7369	-17.1683	7.6939	17.0212
19	7.6841	-16.047	7.7459	-7.9706	7.7012	10.2512
20	7.6902	-7.2614	7.7681	5.4686	7.7415	-17.8814
21	7.716	8.0142	7.7861	-5.2658	7.7619	-29.9521
22	7.7468	-22.3748	7.7908	-10.1186	7.7625	-12.4918
23	7.7486	6.6833	7.8206	-8.1262	7.8064	-18.6392
24	7.8243	-3.753	7.8878	14.5816	7.8153	15.0767
25	7.8385	-5.1113	7.9135	8.3313	7.8342	-0.3292
26	7.8468	-2.4028	7.9425	3.061	7.8716	2.8951
27	7.8775	3.2265	7.9665	-1.2359	7.8814	10.3301
28	7.9214	6.1073	7.9788	-35.5361	7.9097	-3.8911
29	7.9336	5.5335	8.0021	-5.6179	7.9609	-19.33

30	7.9505	-46.5779	8.0249	17.1908	7.9706	1.67
31	7.9644	6.9157	8.0644	17.4753	7.9934	-6.4595
32	7.9987	-1.8367	8.0679	-15.3813	8.0084	19.1132
33	8.026	12.0328	8.1198	-1.0815	8.0296	4.6809
34	8.068	8.3554	8.1398	3.2544	8.0441	-6.5084
35	8.0957	-1.2599	8.1808	-30.004	8.0875	3.7091
36	8.1145	-0.3265	8.1969	-3.941	8.1235	-11.7514
37	8.1883	5.3489	8.2197	-6.1413	8.1459	15.161
38	8.1986	-5.385	8.2493	34.6284	8.1856	-26.4491
39	8.2169	7.198	8.2577	-6.1168	8.1985	4.7843
40	8.2295	0.8877	8.2813	3.911	8.2152	-12.5502
41	8.2444	14.2859	8.3178	-8.6596	8.2286	-0.2344
42	8.2497	-31.3075	8.3389	-2.6452	8.2378	16.859
43	8.2606	7.6881	8.3561	0.0481	8.3263	7.63
44	8.2991	2.7368	8.3864	-6.8822	8.3448	15.9267
45	8.327	17.7194	8.4374	54.7215	8.3788	-14.1886
46	8.3393	1.7033	8.466	16.3811	8.388	16.0222
47	8.3427	-0.6217	8.4791	-31.711	8.3911	8.0116
48	8.3539	11.4885	8.5108	-4.8475	8.4119	-7.2899
49	8.3769	5.0348	8.5234	-32.7646	8.4418	-6.3975
50	8.43	-4.5377	8.5402	-0.1098	8.4609	16.2252
51	8.4678	-17.9859	8.5517	0.5493	8.4974	-36.6586
52	8.4707	3.1484	8.575	-3.5563	8.5201	1.2833
53	8.4763	3.4236	8.5861	-0.2249	8.5291	22.5762
54	8.4964	-9.9251	8.6056	-3.6888	8.5618	-5.1628
55	8.5135	-4.1041	8.628	-8.6605	8.5827	4.7738
56	8.5593	-32.8921	8.6732	1.5586	8.6129	-3.3106
57	8.5847	-8.0166	8.6811	-1.3105	8.6211	-8.0153
58	8.5883	11.6128	8.7112	-12.2268	8.641	0.2427
59	8.6001	-28.0446	8.7293	-6.9247	8.6455	-7.8636

60	8.6223	-2.2943	8.7511	-5.4361	8.6692	-8.8305

State	C4		C5		C6	
	Excitation energies(eV)	Rotatory Strengths*	Excitation energies(eV)	Rotatory Strengths*	Excitation energies(eV)	Rotatory Strengths*
1	3.63	-2.1003	3.6483	2.2587	3.6333	-2.3434
2	5.3265	68.5129	5.4043	28.0907	5.2887	91.3142
3	5.5007	25.8975	5.7053	-56.344	5.535	302.6817
4	5.8848	-214.8558	5.8618	-11.2407	5.8867	-373.8585
5	6.3401	-4.1384	6.4129	-41.816	6.4546	-7.8358
6	6.5144	16.7039	6.6218	1.4792	6.6235	22.7341
7	6.6546	5.1231	6.7115	-13.828	6.7949	34.276
8	6.776	7.7944	6.8037	-5.0367	6.8862	-9.1813
9	6.7952	20.2903	6.9083	6.2994	7.0469	-9.7562
10	7.0509	-10.3792	7.0537	-8.3682	7.1481	1.0296
11	7.1398	-11.7111	7.1552	-0.7477	7.1922	-0.8261
12	7.1905	-0.9752	7.1961	20.0195	7.3497	2.2341
13	7.2667	1.4574	7.2156	-5.4594	7.3875	2.0933
14	7.3349	1.5075	7.3825	-12.7861	7.4582	3.7357
15	7.3817	12.4117	7.4156	-8.3417	7.474	-22.7457
16	7.4574	-6.2961	7.472	38.8898	7.5247	-3.4528
17	7.4722	-9.6425	7.5413	-0.7425	7.5757	-18.7177
18	7.5159	19.094	7.5614	11.7274	7.717	-12.245
19	7.6636	-2.9175	7.6642	3.0039	7.7298	3.5665
20	7.6962	-3.1707	7.687	-2.6276	7.7522	-10.2394
21	7.7193	3.5011	7.6894	-3.5562	7.7742	-2.0435
22	7.7558	-14.227	7.749	-14.1792	7.8045	-4.6569
23	7.8509	-0.3826	7.7714	-2.6307	7.8285	-12.277
24	7.8613	11.0548	7.8417	-1.9213	7.8795	13.9951
25	7.8858	-9.252	7.8842	0.7251	7.894	-1.7765

26	7.9019	0.4232	7.8964	-0.851	7.9178	-23.1515
27	7.9347	7.7412	7.9372	5.6635	7.923	8.38
28	7.9457	-3.8368	7.9888	12.9496	7.9397	5.1522
29	7.9532	-18.3878	8.0014	-2.4443	7.9956	-5.5161
30	7.9732	25.5101	8.0469	15.938	8.0391	22.0153
31	7.9989	-2.8077	8.0554	17.4374	8.0487	9.1067
32	8.0067	-13.1199	8.0595	-3.4341	8.0684	-6.147
33	8.0611	-7.7046	8.0781	1.3426	8.1361	22.937
34	8.075	0.6649	8.0943	-14.5424	8.1453	-1.6084
35	8.1311	-2.7888	8.1194	1.9785	8.1791	2.4616
36	8.1693	-2.9004	8.1342	-18.0339	8.2338	-52.0552
37	8.1876	2.8674	8.1555	0.6004	8.2605	-2.262
38	8.2144	1.5413	8.2022	3.7533	8.2815	-1.2545
39	8.2258	-10.4777	8.2391	17.1727	8.2979	17.9311
40	8.2631	-2.8989	8.261	-9.508	8.3116	0.6386
41	8.2705	30.1473	8.2739	6.5326	8.3297	13.7195
42	8.2852	-1.5706	8.2817	7.4344	8.3408	7.5423
43	8.2995	-10.6266	8.325	-6.6154	8.3687	-23.0041
44	8.3358	-17.5063	8.3428	0.445	8.3935	28.8522
45	8.3386	-25.804	8.3503	-0.4365	8.4083	-5.1802
46	8.3483	8.6899	8.3765	-8.2086	8.4318	-14.5797
47	8.3599	19.9159	8.3875	0.0789	8.4585	-3.1843
48	8.3946	-8.5062	8.3957	1.2803	8.494	-2.4928
49	8.4198	-0.4369	8.4435	21.5687	8.5248	-4.2809
50	8.4246	-5.1071	8.4675	4.7324	8.5597	-0.2885
51	8.4686	1.808	8.4825	-9.1152	8.59	1.0985
52	8.4733	6.5205	8.4945	3.221	8.6204	-24.0821
53	8.4906	-14.849	8.5065	-11.0083	8.6355	-4.1637
54	8.5038	-6.7832	8.5174	-26.8458	8.6445	-18.1338
55	8.5185	-0.9238	8.539	-0.5238	8.6502	-12.2969

56	8.5235	-0.835	8.5405	7.1902	8.6643	7.2463
57	8.5314	-9.3805	8.5613	11.1601	8.6806	6.8332
58	8.5457	12.927	8.5977	-23.2976	8.6888	-11.3791
59	8.5855	-7.9469	8.5989	-2.7294	8.6977	-24.6832
60	8.5895	-11.4806	8.6171	-12.0646	8.7161	-14.9138

* R(velocity)   10**-40 erg-esu-cm

Calculated ECD Data for 6*S*-1 in gas phase

State	C1		C2		C5	
	Excitation energies(eV)	Rotatory Strengths*	Excitation energies(eV)	Rotatory Strengths*	Excitation energies(eV)	Rotatory Strengths*
1	3.6273	10.2098	3.6319	2.4635	3.6512	-2.445
2	5.1591	-255.5807	5.2902	-94.9621	5.4237	-98.2994
3	5.7587	-46.8657	5.5345	-251.7238	5.6904	136.0594
4	5.8287	220.7934	5.8906	360.4816	5.8563	-35.3308
5	6.5295	43.5497	6.4542	1.407	6.5319	45.4715
6	6.5969	-16.0377	6.6173	-23.3009	6.804	14.0996
7	6.912	-4.9317	6.7994	-35.7296	6.8833	-8.3668
8	6.9364	-10.1724	6.8762	-1.6451	6.9005	-14.3367
9	7.0126	-8.1092	7.0451	11.1316	7.0219	6.0641
10	7.1264	5.9494	7.1573	2.443	7.1508	-9.807
11	7.1714	0.3285	7.1783	4.337	7.2094	2.1476
12	7.2602	-13.4006	7.3435	0.6972	7.2876	2.6044
13	7.4172	1.8466	7.3905	4.2464	7.4095	6.0873
14	7.4657	7.526	7.4671	14.3013	7.4657	-1.411
15	7.5036	5.3698	7.4766	-4.2554	7.5112	-5.9354
16	7.5407	-17.3135	7.518	10.0158	7.5525	7.1239
17	7.6799	-21.2953	7.579	-29.8174	7.5922	-23.0035
18	7.7384	6.131	7.6954	-10.6197	7.6924	-17.293
19	7.7472	-15.1656	7.7477	-13.0905	7.7391	-4.808
20	7.7554	2.291	7.7522	2.1028	7.7468	-11.9065

21	7.7721	-11.4577	7.7665	8.2095	7.7726	-4.202
22	7.7885	-7.666	7.7973	-4.7912	7.7946	-4.0024
23	7.8117	20.1623	7.8645	18.4438	7.8092	-14.8794
24	7.8857	-3.4187	7.8756	-24.0907	7.8342	-4.0106
25	7.9162	-8.52	7.8956	6.0207	7.9335	-1.1073
26	7.9411	0.5482	7.915	27.5255	7.9415	0.915
27	7.9598	-0.8647	7.9433	21.7024	7.9936	-25.0139
28	7.9739	35.3084	7.9458	-25.5534	7.9969	-6.6837
29	8.001	-4.9313	7.9999	-5.3388	8.0216	17.5988
30	8.0268	18.3213	8.0275	23.1472	8.0348	21.9872
31	8.0662	-15.6226	8.0481	-37.9013	8.0633	14.1981
32	8.0679	-25.3862	8.0652	-12.193	8.07	-26.2643
33	8.1152	0.0102	8.1185	-2.0679	8.1418	1.317
34	8.1283	-14.1575	8.1334	-32.0652	8.1897	19.564
35	8.1864	16.5562	8.1801	9.7171	8.2289	-29.532
36	8.2035	-0.3553	8.2108	10.5827	8.2513	-10.0704
37	8.2409	-6.0887	8.2435	-8.5123	8.2608	-2.367
38	8.2572	4.1642	8.2549	36.9584	8.2686	14.6538
39	8.2727	2.6958	8.2852	-6.5954	8.3148	-0.6638
40	8.2827	-8.84	8.2954	-6.8585	8.3332	-35.9358
41	8.3232	-1.7194	8.3037	7.8728	8.3924	16.0796
42	8.345	1.2614	8.3306	-7.0778	8.3989	-3.8191
43	8.3552	10.5336	8.3585	1.098	8.4103	-12.3984
44	8.3875	10.6099	8.3889	-29.4283	8.4456	-1.3491
45	8.4521	-47.1811	8.4068	5.3228	8.4549	10.1175
46	8.4708	-1.8042	8.4318	16.8367	8.4824	-2.7284
47	8.4832	27.2003	8.445	-0.7757	8.5059	-1.9188
48	8.5145	10.0007	8.4816	-1.9999	8.5386	-0.3518
49	8.5206	17.1631	8.5143	-1.9049	8.5509	20.679
50	8.5347	-1.7602	8.5224	0.4266	8.5668	-2.8271

51	8.5518	-9.3328	8.6127	-7.7281	8.5896	-35.9124
52	8.5709	-4.014	8.6289	-12.3628	8.62	-11.4431
53	8.5823	-1.9467	8.6365	9.8615	8.6361	-2.0355
54	8.6125	-3.5675	8.6448	22.3822	8.638	-2.1282
55	8.6305	-12.288	8.6515	-1.9509	8.6646	-5.2496
56	8.6565	5.2768	8.6555	-7.0946	8.6864	-22.5971
57	8.6874	-7.3042	8.6644	-0.8828	8.7007	-4.6619
58	8.7173	1.8934	8.6682	14.8251	8.7096	-6.3709
59	8.7278	7.8405	8.7052	-11.1789	8.7218	-5.1739
60	8.7488	-10.4564	8.7209	0.7574	8.7528	13.5068

* R(velocity) 10**-40 erg-esu-cm
